# Neutropenia as an Adverse Event following Vaccination: Results from Randomized Clinical Trials in Healthy Adults and Systematic Review

**DOI:** 10.1371/journal.pone.0157385

**Published:** 2016-08-04

**Authors:** Vincent Muturi-Kioi, David Lewis, Odile Launay, Geert Leroux-Roels, Alessandra Anemona, Pierre Loulergue, Caroline L. Bodinham, Annelies Aerssens, Nicola Groth, Allan Saul, Audino Podda

**Affiliations:** 1 Novartis Vaccines Institute for Global Health, Siena, Italy; 2 Surrey Clinical Research Centre, University of Surrey, Guildford, United Kingdom; 3 Université Paris Descartes, Sorbonne Paris cité, and Inserm CIC 1417, F-CRIN I-Reivac, Assistance Publique Hôpitaux de Paris, CIC Cochin-Pasteur, Paris, France; 4 Ghent University and University Hospital, Gent, Belgium; 5 Novartis Vaccines & Diagnostics, Siena, Italy; George Washington University School of Medicine and Health Sciences, UNITED STATES

## Abstract

**Background:**

In the context of early vaccine trials aimed at evaluating the safety profile of novel vaccines, abnormal haematological values, such as neutropenia, are often reported. It is therefore important to evaluate how these trials should be planned not to miss potentially important safety signals, but also to understand the implications and the clinical relevance.

**Methodology:**

We report and discuss the results from five clinical trials (two with a new *Shigella* vaccine in the early stage of clinical development and three with licensed vaccines) where the absolute neutrophil counts (ANC) were evaluated before and after vaccination. Additionally, we have performed a systematic review of the literature on cases of neutropenia reported during vaccine trials to discuss our results in a more general context.

**Principal Findings:**

Both in our clinical trials and in the literature review, several cases of neutropenia have been reported, in the first two weeks after vaccination. However, neutropenia was generally transient and had a benign clinical outcome, after vaccination with either multiple novel candidates or well-known licensed vaccines. Additionally, the vaccine recipients with neutropenia frequently had lower baseline ANC than non-neutropenic vaccinees. In many instances neutropenia occurred in subjects of African descent, known to have lower ANC compared to western populations.

**Conclusions:**

It is important to include ANC and other haematological tests in early vaccine trials to identify potential safety signals. Post-vaccination neutropenia is not uncommon, generally transient and clinically benign, but many vaccine trials do not have a sampling schedule that allows its detection. Given ethnic variability in the level of circulating neutrophils, normal ranges taking into account ethnicity should be used for determination of trial inclusion/exclusion criteria and classification of neutropenia related adverse events.

**Trial registration:**

ClinicalTrials.gov NCT02017899, NCT02034500, NCT01771367, NCT01765413, NCT02523287

## Introduction

Understanding vaccine safety requires a comprehensive assessment of all events that occur after the administration of a vaccine. Adverse events following immunization are defined as any untoward medical occurrence which follows vaccine administration and which does not necessarily have a causal relationship with the usage of the vaccine. Characterization of a vaccine’s safety profile takes place throughout its development and during its entire life cycle following the first marketing authorization. As vaccines are usually administered to healthy individuals, the opportunity to collect data on adverse events characterized by laboratory anomalies is usually confined to the clinical development period when laboratory samples can be collected in a protocol-specific manner from study participants.

A previous example where an adverse event, identified by an abnormality in a laboratory parameter, has been associated with administration of a vaccine, is idiopathic thrombocytopenic purpura, linked to the administration of measles-containing vaccines. The identification of this association required several retrospective observational studies analyzing data from millions of young children over the past two decades [1, 2]. Therefore, it is important to collect reactogenicity data that would potentially signal a safety concern, early in development as it allows a more accurate characterization of benefit-risk and ensures that patients and trial subjects are protected. A clear understanding of the potential risks early on will allow for more comprehensive assessments prospectively following licensure and will provide more information to be availed to the public thus increasing trust in the vaccination program.

The aim of this manuscript is to discuss the implications on the design of early phase vaccine trials with regard to subject recruitment, screening and post-vaccination follow-up of enrolled subjects for identifying adverse events characterized by abnormal hematological values, such as abnormally low neutrophil counts (i.e., neutropenia). Neutropenia is normally caused by several conditions including infections, drug treatments, autoimmune diseases, nutritional deficiencies, hematological malignancies, but also by genetic conditions like the benign ethnic neutropenia (BEN). This latter condition, which affects particularly populations of African descent and is considered to be due to a regulatory variant in the Duffy antigen receptor for chemokines gene is not associated with an increased incidence of infection [3–8]. Additionally, we would like to discuss how post registration clinical trials can be useful in identifying, for licensed vaccines, potential biomarkers of reactogenicity, including hematological abnormalities and how these trials can help to put in the right perspective similar findings from early clinical trials with novel vaccines. Finally, we would like to highlight the clinical relevance and the implications of cases of neutropenia temporally associated with vaccination in otherwise healthy individuals. The first section of this article includes a description of neutrophil counts and their kinetics following administration of both a novel candidate vaccine against *Shigella* in two phase I trials and of well-known licensed vaccines in three post-registration clinical trials. The second section includes a review of publications relating to vaccine clinical trials where neutropenia was noted as an adverse event following immunization.

## Materials and Methods

### Clinical Studies with the GVGH GMMA Vaccine against *Shigella sonnei*

**Fig 1:**
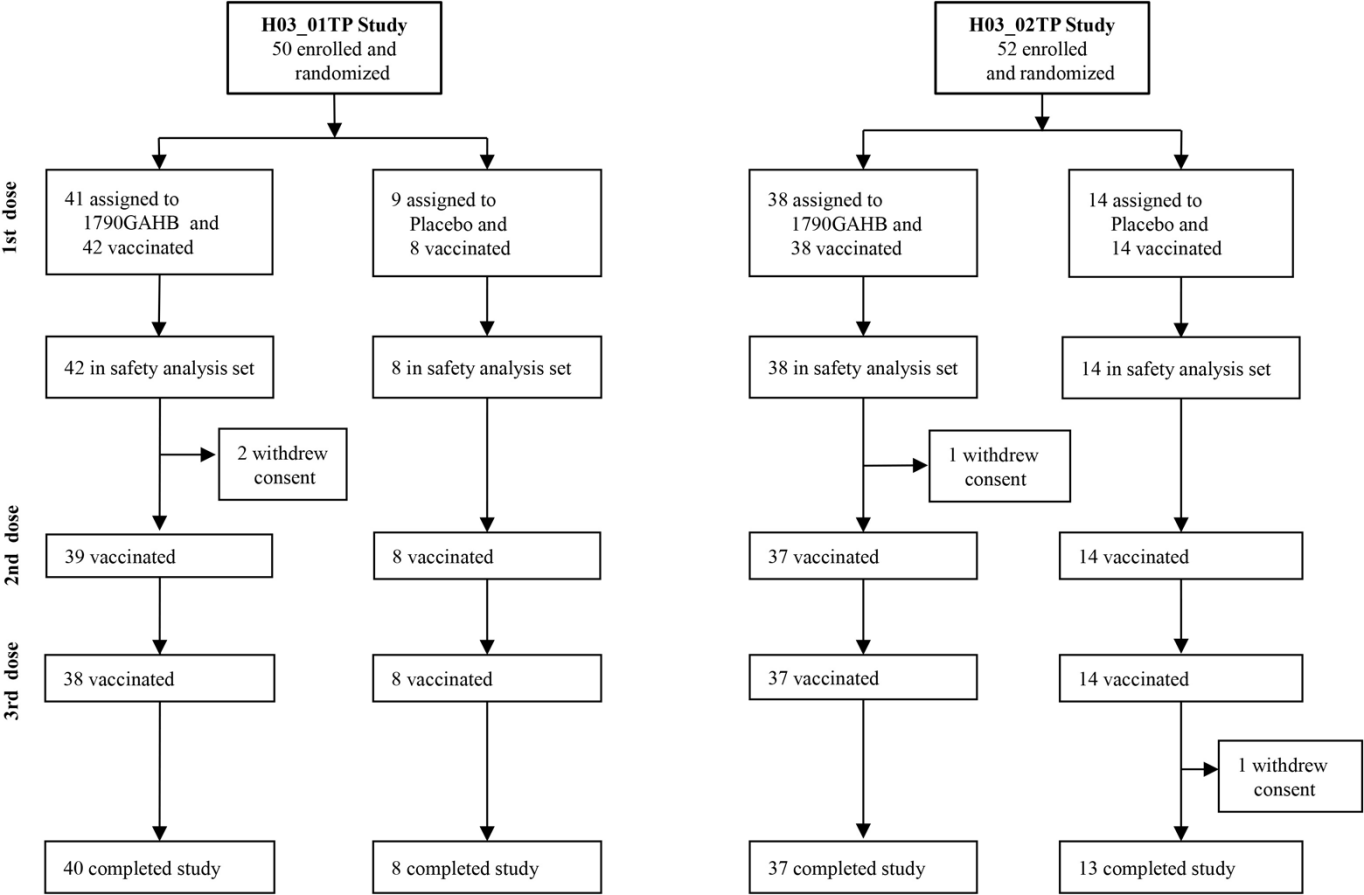
H03_01TP and H03_02TP study flow charts.

Two phase I randomized, placebo controlled, dose-escalation trials assessing safety and immunogenicity of a new vaccine against *Shigella sonnei* (1790GAHB), developed by the GSK Vaccines Institute for Global Health (GVGH) using the Generalized Modules for Membrane Antigens (GMMA) technology [9], were conducted in healthy European adults aged 18 to 45 years in the context of EC funded FP7 projects STOPENTERICS (http://stopenterics.bio-med.ch/cms/default.aspx) and ADITEC (http://www.aditecproject.eu/), respectively. Trial 1 (H03_01TP, registered in ClinicalTrials.gov as NCT02017899 and ethically approved by the “Comité de Protection des Personnes (CPP) Ile de France III”, reference: 2013-0026227-42) was conducted in France and used three intramuscular (IM) vaccine administrations (given one month apart) to induce systemic immunity testing 5 different doses (1, 5, 25, 50, 100 μg of GMMA protein). Trial 2 (H03_02TP, registered as NCT02034500 and ethically approved by UK National Research Ethics Service (NRES) London—Surrey Borders Committee, reference: 13/LO/1633) was conducted in the UK, used the same schedule and tested three doses (0.1, 1, 10 μg of GMMA protein) by intradermal (ID) route and three doses (5, 20, 80 μg of GMMA protein) by intranasal (IN) route to induce systemic and mucosal immunity and, as a link to trial 1, included also a group receiving three intramuscular vaccine administrations with 5 μg/dose (Fig 1). The vaccine was adsorbed onto 0.35 mg, 0.035 mg and 0.28 mg of Al^3+^, as Alhydrogel, per each IM, ID and IN dose, respectively. Same amounts of Alhydrogel in Tris-buffered saline, were used for the respective IM, ID and IN placebo formulations. Overall, in the two trials, 80 subjects received vaccine and 22 placebo. As part of the safety follow up, performed throughout the trial, hematology, blood chemistry and urinalysis tests were performed at different time points before and after each vaccine administration. According to the study protocol for both trials, safety blood samples were obtained before and 7 days after the first dose and 28 days after the second and the third dose. Clinically significant changes of laboratory test results were defined by investigators based on medical judgment, interpretation of deviations from respective institution’s normal values and recommendations from the Center for Biologics Evaluation and Research (CBER) FDA guidance for industry [10]. According to this note for guidance, decreases in absolute neutrophil counts (ANC) are coded as grade 1 to grade 4 (Table 1).

**Table 1:** Absolute Neutrophil Counts Grading.

grade 1	ANC 1500-2000/mm3
grade 2	ANC 1000-1499/mm3
grade 3	ANC 500-999/mm3
grade 4	ANC <500/mm^3^

Subsequent to 2 episodes of grade 3 neutropenia following the first dose of vaccine (one in each of the 2 trials) the sponsor and investigators, in consultation with the Data Safety Monitoring Board (DSMB), and ethical and regulatory authorities for the two trials, implemented a more conservative approach for follow-up of study subjects, including collection of blood samples also at 7 days after the second and the third dose and at the end of the study (i.e., 6 months after the last vaccine dose).

### Clinical studies with three different licensed vaccines

**Fig 2:**
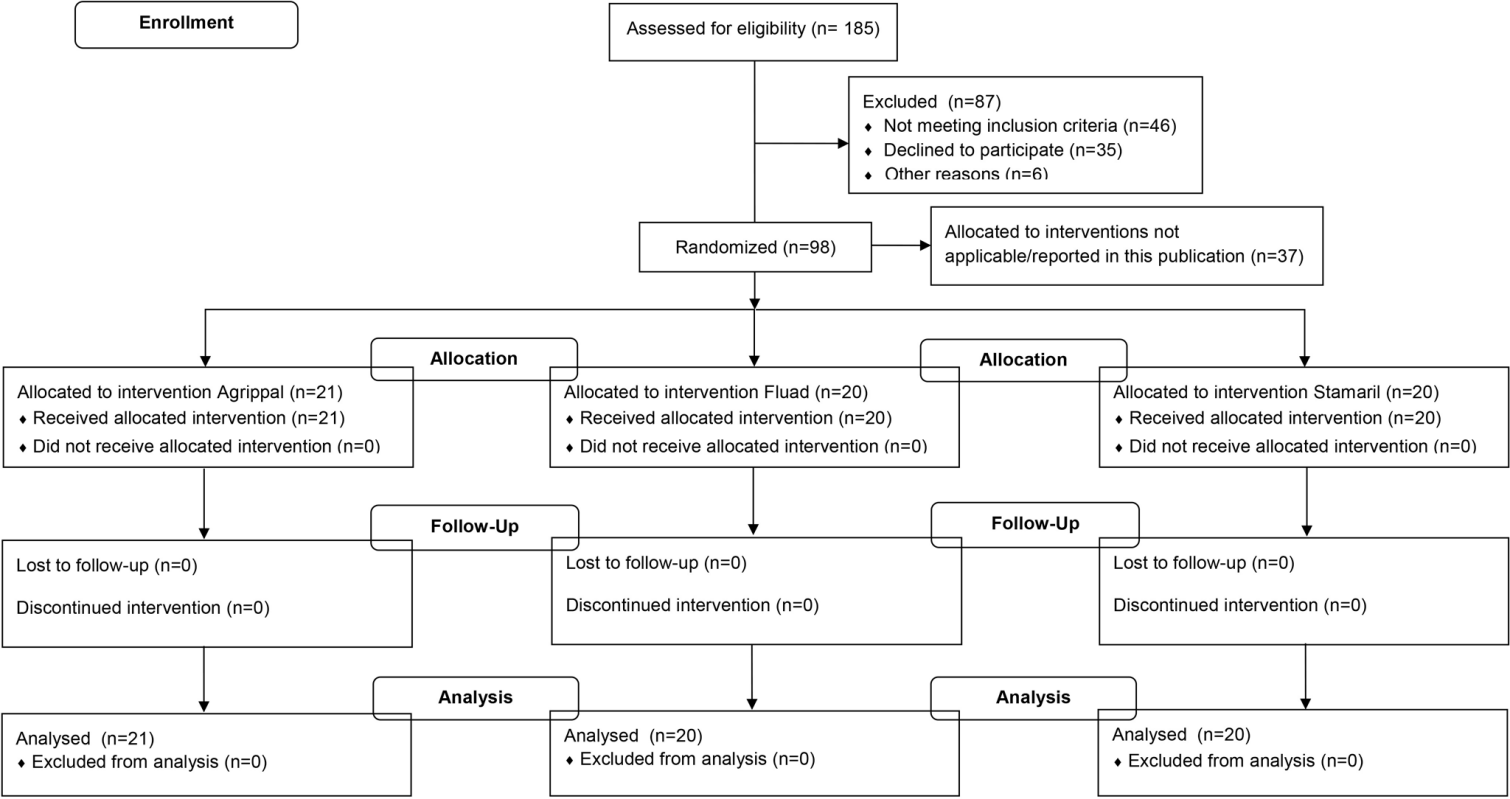
CRC305A and CRC305C study flow charts.

As part of a series of clinical studies to identify biomarkers of vaccine reactogenicity, in the EC funded FP7 project BioVacSafe (http://www.biovacsafe.eu/), healthy adult male and female volunteers were recruited to a single UK clinical site (same site where the 1790GAHB vaccine trial took place) to receive a single vaccination with the recommended adult dose of the following licensed vaccines: Agrippal (Novartis Vaccines, seasonal trivalent influenza surface antigen, inactivated, not adjuvanted, n = 21), Fluad (Novartis Vaccines, seasonal trivalent influenza surface antigen, inactivated, adjuvanted with MF59C.1, n = 20), or Stamaril (Sanofi Pasteur, live, attenuated yellow fever virus 17 D-204 strain, n = 20). Ethical approvals were obtained from the UK NRES London—Surrey Borders Committee (reference 12/LO/1871, 13/LO/0044). Participants receiving Stamaril were naïve to yellow fever as confirmed by serology and immunization history. All participants provided written informed consent prior to enrolment. Studies were registered on ClinicalTrials.gov (NCT01771367, NCT01765413). The participants had an overall age range of 18–45 years (median 28, mean 28), and were deemed healthy by virtue of confirmed medical history, examination and baseline blood tests. They were kept as inpatients for a day before vaccination, and during the first 5 days post vaccination, with controlled diet, exercise and sleep routines. Blood samples were taken before, daily for the first seven days and weekly up to day 28 after vaccination, and analyzed for various safety parameters including automated Full Blood Count with white cell differential (Fig 2). However, for the purpose of this paper, we only report data on the absolute neutrophil count on days 0–7 (and 0–7, 14, 21and 28 for Stamaril), with a laboratory lower limit of normal range = 2000/mm^3^.

**Fig 3:**
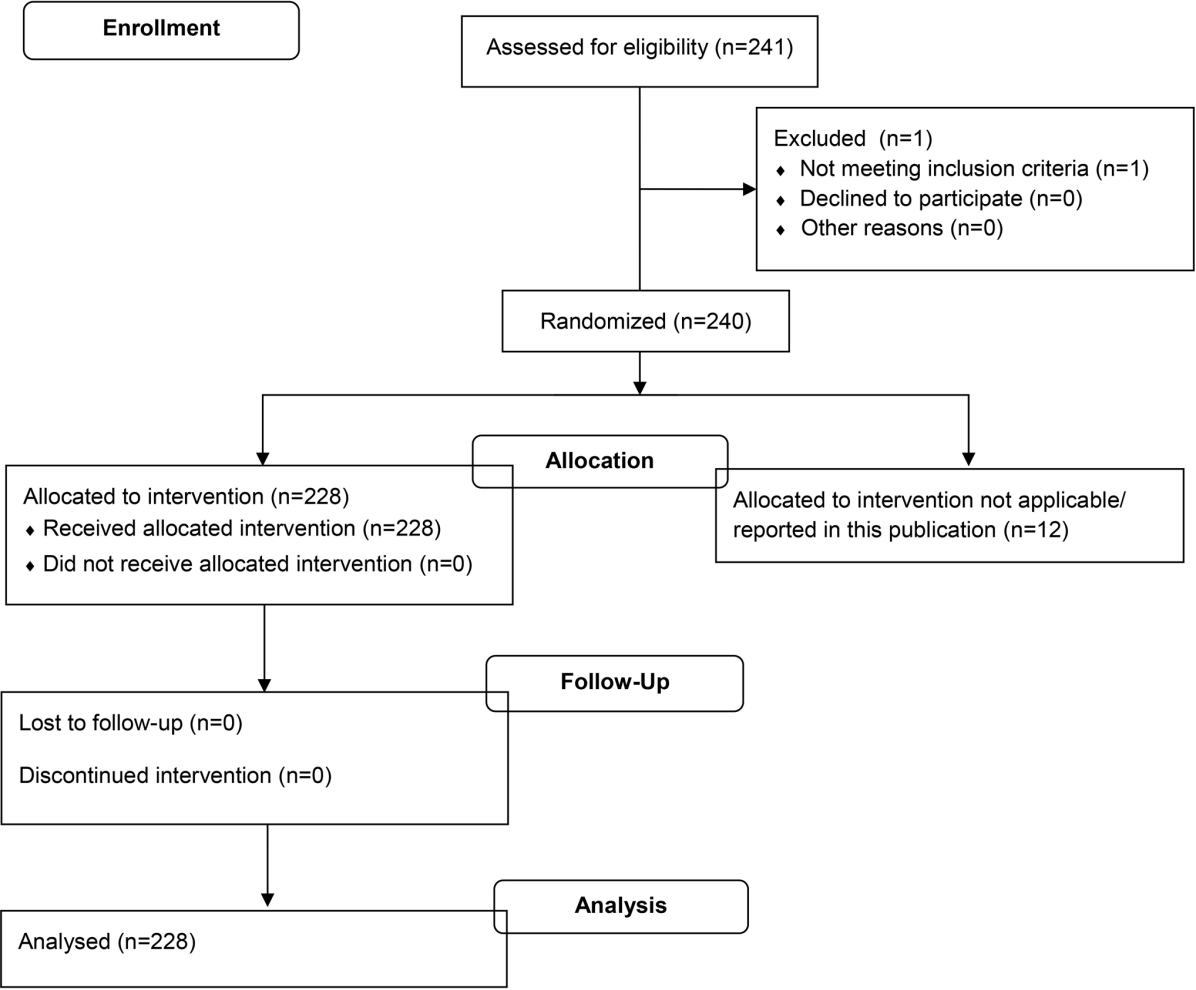
Ghent trial study flow chart.

In a follow-on clinical study within the BioVacSafe project, 228 healthy adult male and female volunteers (age range of 18–45 years, median 24, mean 27) were recruited to a single Belgian clinical site to receive a single vaccination with the adult dose of Fluad (Novartis Vaccines, seasonal trivalent influenza surface antigen, inactivated, adjuvanted with MF59C.1). The study was registered as NCT02523287 and ethically approved by the University of Gent Ethical Committee– 2014/0733). All visits were made as outpatients on days 0, 1, 7 (n = 114) or 0, 1, 3, 7, 21 (n = 114) (Fig 3). Also for this trial, we only report here data on ANC on days 0, 1, 3, 7, with a laboratory lower limit of normal range = 1573/mm^3^.

### Literature Review

With the literature review, we sought to determine how many articles accessible via PubMed and reporting data from phase I and phase II vaccine clinical trials, where hematological testing is more often made, reported the presence of neutropenia as an adverse event following vaccination. We included only articles relating to phase I and phase II vaccine clinical trials enrolling otherwise healthy participants and thus excluded trials that enrolled special populations with underlying health conditions, for example, trials conducted in patients with malignancy. In order to increase the sensitivity of our search, QUOSA software (Elsevier B.V.) was used to download and search each of the articles for the key words related to our search.

An initial search of PubMed was performed on 21^st^ August 2014. The search included all years with the first search string including the terms vaccine in combination with the publication types (clinical trial phase i or clinical trial phase ii) and the Medical Subject Headings (MESH) term humans. This returned 1931 articles. To ensure that the primary search term did not exclude relevant articles, a second search was performed with the alteration to exclude the MESH term humans. This returned 12 articles none of which fit the criteria of a phase I or phase II vaccine trial performed on humans (Table 2).

**Table 2:** First PubMed Search Results.

vaccine	AND	“clinical trial, phase i” [Publication Type] OR“clinical trial phase ii” [Publication Type]	AND	humans[Mesh Terms]	1931 articles
vaccine	AND	“clinical trial, phase i” [Publication Type] OR“clinical trial phase ii” [Publication Type]	NOT	humans[Mesh Terms]	12 articles

Of the 1931 articles that were retrieved, we attempted to download all full text publications. The ability to access full length articles was dependent on the company journal subscription and the availability of free on line publications. A total of 1653 full length articles were accessible, while the remaining 278 articles were accessed as abstracts only.

The QUOSA software was used to search through each individual article and apply an exclusion pattern to eliminate articles that dealt with cancer patients as these would not fit our inclusion criteria of phase I and phase II vaccine clinical trials performed in otherwise healthy subjects. The exclusion pattern read; NOT (cancer OR neoplasm OR tumor OR carcin* OR metastatic OR melanoma OR lymphoma). This returned 771 articles from the original 1931 publications assessed. Of these, 67 included the key words ‘neutropenia OR neutrophil*’ and 30 of these just included the word ‘neutropenia’. All 67 articles were read to search for information related to the safety assessment. Of the 30 with the keyword neutropenia, 25 described safety blood draws with noted neutropenia post vaccination, 2 articles did not describe any safety blood draws for hematology and 3 articles were not vaccine trials. Of the remaining 37 articles with the word neutrophil* but not neutropenia, 5 articles described neutropenia or leukopenia following vaccination, 14 articles described safety blood draws being assessed post vaccination with no abnormalities detected, 15 articles did not provide any information on blood samples for safety post vaccination, and 3 articles were not vaccine trials.

While reviewing one of these 67 articles [11], a reference was found citing another article [12] that described neutropenia as an adverse event following vaccination in a phase I vaccine clinical trial. This brought the total number of articles returned by this search string to 31.

In order to look for articles that reported results from clinical trials but were not classified as such in PubMed, a second search string was used to search for articles on vaccines that referred to phase I, phase 1, phase II or phase 2 anywhere in the PubMed searchable text, but that were not classified as ‘clinical trial, phase i’ or ‘clinical trial, phase ii’ publication types, while at the same time excluding the publication types ‘review’, ‘comment’, and ‘editorial’ (Table 3).

**Table 3:** PubMed Search Strings.

Vaccine AND (‘phase 1’ OR ‘phase 2’ OR ‘phase i’ OR ‘phase ii’) AND humans [MeSH Terms]	NOT	‘clinical trial phase i’ [Publication Type] OR ‘clinical trial phase ii’ [Publication Type]	NOT	review [publication Type] comment [Publication Type] editorial [Publication Type]

This search returned 1005 articles that were downloaded. The QUOSA software was used to search through individual articles and apply inclusion criteria to return articles that described subject enrolment. The search was done for the keywords (volunteer* OR subject* OR recruit* OR patient*). This returned 764 articles, and to these, a similar exclusion pattern as described above was applied to eliminate articles that dealt with cancer. This left 325 articles, and a further search using QUOSA found 13 of these articles contained the keywords neutrophil* or neutropenia. Out of these, 3 articles described phase I or phase II vaccine trials where neutrophils were measured post vaccination; 2 of these articles described trials where neutropenia was detected as an adverse event post vaccination.

The first search string was repeated on 23^rd^ of April 2015 with the intention of finding articles that may have fit our inclusion criteria and been published after the 21^st^ of August 2014, using the terms (vaccine AND ("clinical trial, phase i"[Publication Type] OR "clinical trial, phase ii"[Publication Type])) AND humans[MeSH Terms]. This returned 47 papers which were downloaded. Of these 45 were available as full length articles. These 47 were searched in QUOSA for the word “neutropenia” and this returned 5 articles. A review of the full length articles revealed that two articles reported results from trials of a cancer immunogen and a cancer vaccine and were therefore excluded, and one dealt with a phase I trial for a dengue vaccine candidate during which no cases of neutropenia were reported. These three articles were thus excluded. The other two articles reported the results of phase I and phase II vaccine trials where neutropenia was noted as an adverse event and were therefore included.

**Fig 4:**
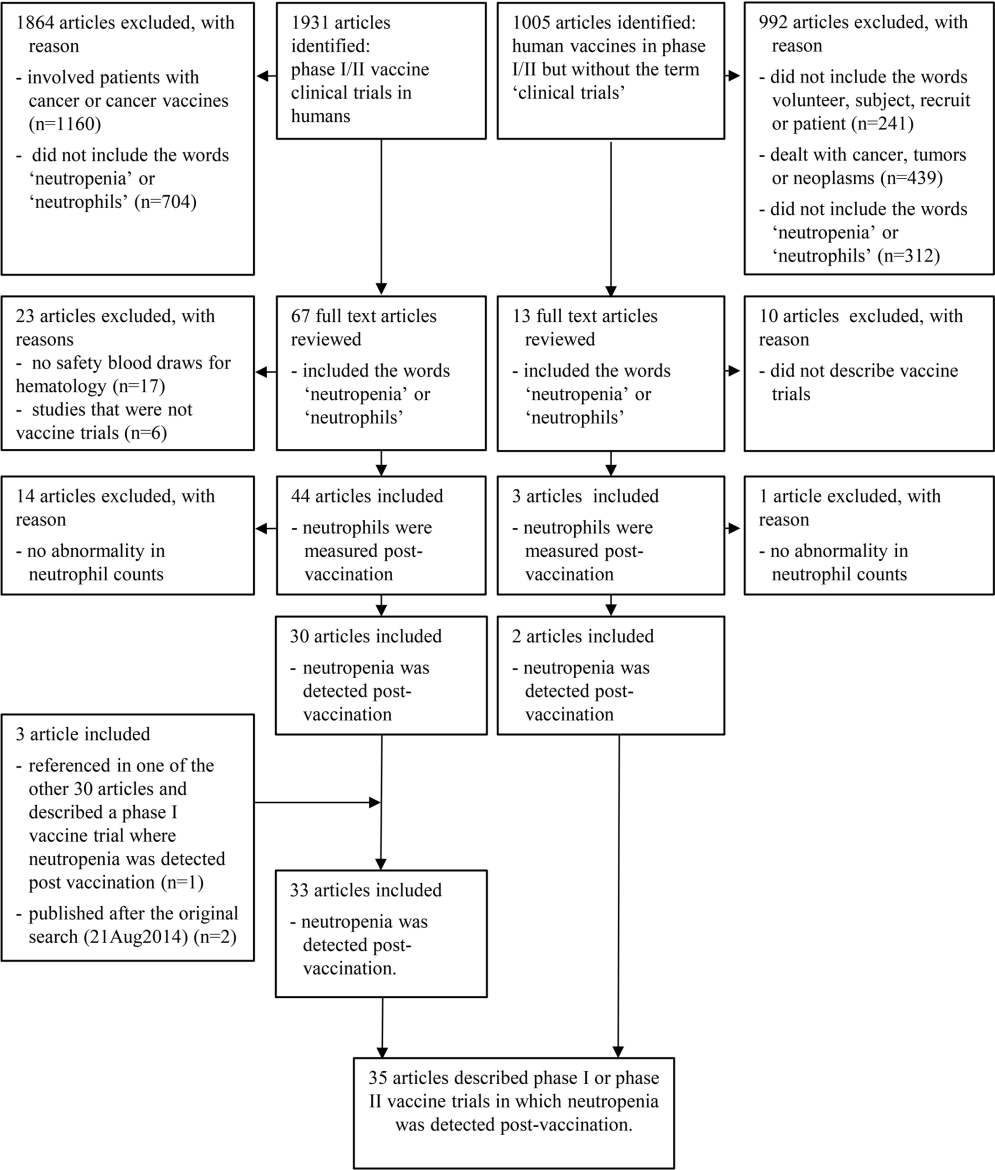
Literature search flow-chart.

These two search strings returned a total of 35 articles where neutropenia was noted as an adverse event following vaccination in phase I and phase II vaccine clinical trials available on PubMed (Fig 4 and Table 4) These 35 articles were read and data extracted for description of: the type of vaccine, presence of an adjuvant, the time taken from vaccination to event detection and the mean day of onset of neutropenia, the mean duration of time to recovery, the mean baseline absolute neutrophil counts, the mean absolute decline in the neutrophil count, the grading of the neutropenia, association with host factors such as race, gender, and age, recurrence of cases upon re-challenge and complications that may have been noted that were associated with the neutropenia. Two of the authors (VMK and AS) checked each of the papers assessed in detail for exclusion or inclusion and for those included, for extracting details of the type of trial etc.

**Table 4:** Vaccines Associated With Neutropenia Cases in Articles Selected in the Literature Search.

Article’s author	Antigen/Pathogen	Type of Vaccine	Adjuvant	Reference ♯
Durbin AP, et al.	DEN1Δ30 / Dengue 1	Live attenuated	None	[11]
Morrison D, et al.	Tetravalent chimeric / Dengue	Live attenuated	None	[12]
Cunningham CK, et al.	GP120 /HIV	rProtein	Alum/ MF59	[13]
Kotloff KL, et al.	Hexavalent rec. peptides /GAS	rSubunit	Alum	[14]
Ellis RD, et al.	AMA1/C1/Malaria	rProtein	Alum+CpG	[15]
Duncan CJ, et al.	AMA1/C1/Malaria	rProtein	Alum+CpG	[16]
Sheldon EA, et al.	rLP2086 / meningococcus B	rProtein	None	[17]
McFarland EJ, et al.	vCP1452 / HIV1 rgp120 / HIV1	Viral vectored rProtein	None Alum	[18]
Dieye TN, et al.	MVA 85A / TB	Viral Vectored	None	[19]
Durbin AP, et al.	rDEN1Δ30 / Dengue 1	Live attenuated	None	[20]
Edelman R, et al.	Tetravalent / Dengue	Live attenuated	None	[21]
Jaoko W, et al.	MVA.HIVA / HIV1 pTHr.HIVA / HIV 1	Viral vectored DNA vectored	None None	[22]
Karron RA, et Al.	H5N1 VN 2004/Aaca / Avian Flu	Live attenuated	None	[23]
Kitchener S, et al.	Tetravalent / Dengue	Live attenuated	None	[24]
McArthur JH, et al.	rDen4Δ30–200,201; Dengue 4	Live attenuated	None	[25]
Ouedraogo A, et al.	Ad35CS01 / Malaria	Viral vectored	None	[26]
Sun W, et al.	Tetravalent / Dengue	Live attenuated	None	[27]
Talaat KR, et al.	H7N3 / Influenza	Live attenuated	None	[28]
Chuang I, et al.	CS-DNA/AMA-Ad / Malaria	DNA prime -Adenovirus boost	None	[29]
Sedegah M, et al.	NMRC-M3V-Ad-PfCA / Malaria	Viral vectored	None	[30]
Watanaveeradej V, et al.	Tetravalent / Dengue	Live attenuated	None	[31]
Chang LJ, et al.	E1, E2, and capsid proteins /Chikungunya	virus-like particle	None	[32]
Njuguna IN, et al.	MVA.HIVA	Recombinant non replicating virus	None	[33]
Durbin AP, et al.	rWN/DEN4Δ30 / Dengue	Live attenuated	None	[34]
Sun W, et al.	Monovalent (1–4) & Tetravalent / dengue	Live attenuated	None	[35]
Capeding RZ, et al.	Tetravalent / Dengue	Live attenuated	None	[36]
Durbin AP, et al.	rDEN2/4Δ30 / Dengue 2	Live attenuated	None	[37]
Vardas E, et al.	tgAAC09- HIV1	Viral vectored	None	[38]
Kanesa-thasan N, et al.	ALVAC / Japanese Encephalitis NYVAC / Japanese Encephalitis	Viral vectored Viral vectored	None None	[39]
Gershon AA, et al.	Varicella-Zoster Oka virus	Live attenuated	None	[40]
Lindow JC, et al.	DEN1Δ30;DEN2/4Δ30; DEN3Δ30/31	Live attenuated	None	[41]
Siberry GK, et al.	Men A, C, Y and W-135-DT	Conjugate	None	[42]
Siberry GK, et al.	Men A, C, Y and W-135-DT	Conjugate	None	[43]
Wright PF, et al.	LGT/DEN4- Tick Borne Encephalitis	Live attenuated	None	[44]
Sanchez V, et al.	VDV3 / Dengue	Live attenuated	None	[45]

## Results

### Neutrophil count kinetics in the *Shigella* vaccine trials

Overall, data from the two phase I trials with the GVGH GMMA *Shigella* vaccine indicate a good safety profile even at doses as high as 100 μg (i.e. 4-fold higher than Outer Membrane Vesicles dose in licensed vaccines) by IM route, 80 μg by IN route and 10 μg by ID route. Only one case of moderate fever was observed in H03_01TP and two in H03_02TP. In this manuscript we discuss results relating to neutrophil counts in the above studies.

**Fig 5:**
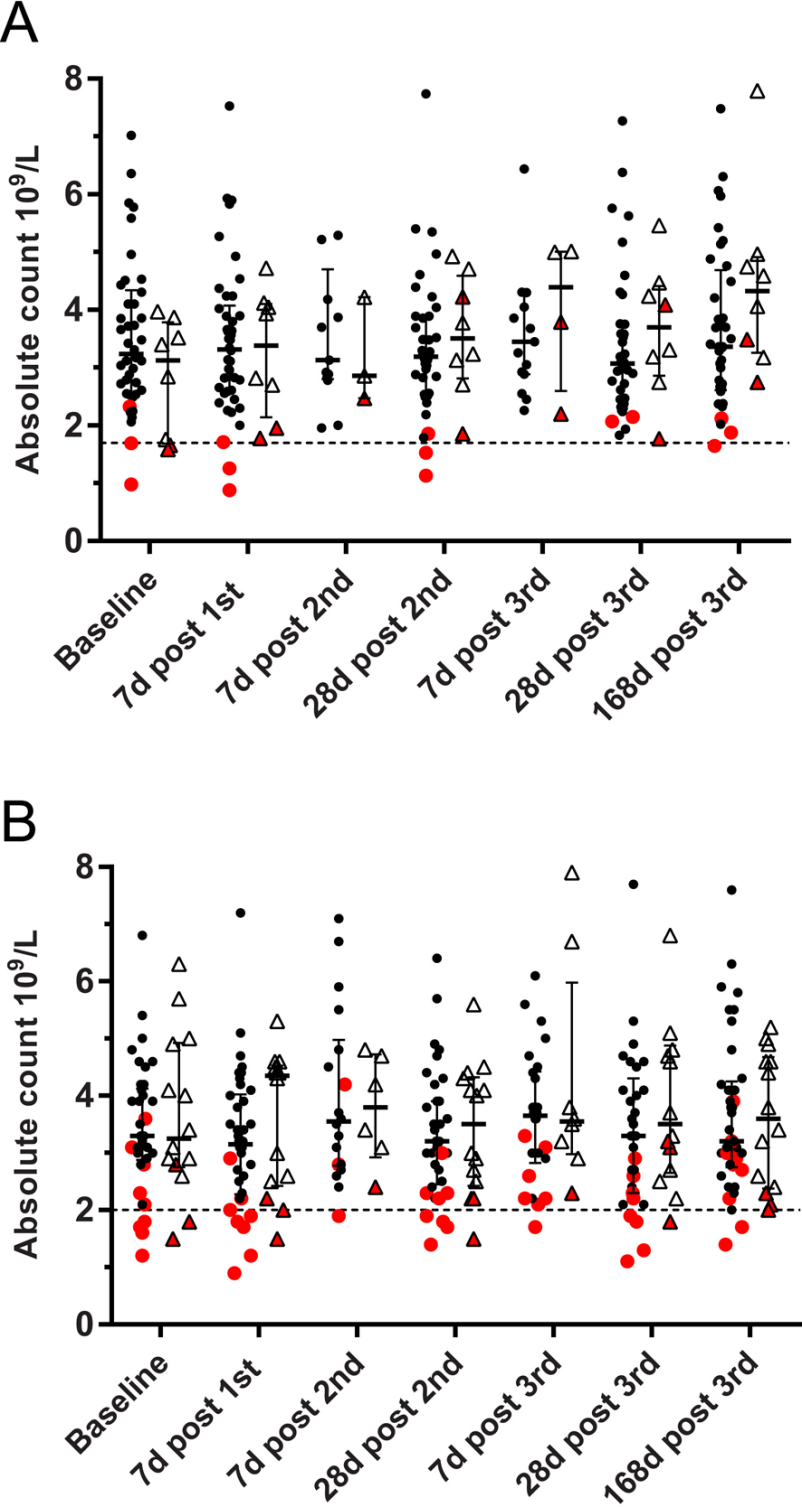
Absolute neutrophil count at different time points after vaccination with three doses of either 1790GAHB or placebo at 0, 1, and 2 months. (A) H03_01TP study. (B) H03_02TP study. Red dots indicate subjects who had an absolute neutrophil count less than laboratory normal range on at least one time point after vaccination. Baseline data are collected during the screening of volunteers. Bars indicate median and interquartile range. At each time point, dots relating to ANC in vaccinees are on the left hand side and dots relating to ANC in placebo recipients are on the right hand side.

As shown in Fig 5, several instances of ANC values below 2000/mm^3^ occurred both in the vaccine and in the placebo groups of the two trials. It should be noted though that the lower limit of the normal range at the two sites was different (1700/mm^3^ in France, H03_01TP study, and 2000/mm^3^ in the UK, H03_02TP study). Altogether, in the two trials, we have seen 2 cases of grade 3 neutropenia (one in each trial) and 6 cases of grade 2 neutropenia (1 case in H03_01TP and 5 cases in H03_02TP). All cases of grade 2 and grade 3 neutropenia were reported in vaccinees (N = 80) but not in placebo recipients (N = 22). Baseline median ANC in vaccinated subjects with cases of neutropenia below 1500/mm^3^ at any time during the trial was 2200/mm^3^ and was significantly lower (p = 0.0026, Mann-Whitney Test) than that in vaccinated subjects showing no neutropenia below 1500/mm^3^ throughout the study (i.e., 3355/mm^3^).

The two cases of CBER grade 3 neutropenia were reported at day 7 after the first vaccination with either 50 μg vaccine by IM (H03_01TP) or 20 μg vaccine by IN (H03_02TP) (Table 5). Both subjects were of African descent, were clinically well at the time of the observation and did not show any clinically significant local or systemic reaction after vaccination. An additional blood draw, obtained on day 20 and on day 14 showed an ANC of 1360/mm^3^ and of 2600/mm^3^ compared to 880/mm^3^ and 900/mm^3^ at day 7, respectively (Table 5). These two events were classified by investigators of both trials as severe adverse events, but in the absence of any seriousness criteria they were not considered Serious Adverse Events (SAE).

**Table 5:** Grade 3 Cases of Neutropenia Occurred in two Trials After Administration of the First Dose of the Shigella GMMA Vaccine.

	Case 1 (H03_01TP study)	Case 2 (H03_02TP study)
Group	50 μg/IM route	20 μg/IN route
Age (yrs)	37	21
Gender	Female	Female
Ethnicity	Black/African descent	Black/African descent
Neutrophils count		
	@ day-21: 2330/mm^3^	@ day-14: 1800/mm^3^
	@ day 7: 880/mm^3^ (grade 3)	@ day 7: 900/mm^3^ (grade 3)
	@ day 20: 1360/mm^3^ (grade 2)	@ day 14: 2600 /mm^3^

Of the 6 cases of grade 2 neutropenia, 3 occurred in subjects of African descent and 3 in Caucasians. Two of these subjects had one episode of neutropenia either at 1 month or at 6 months after the last vaccination while all previous results were within the normal range. Another of these subjects was already neutropenic at baseline (i.e., 980/mm^3^) and had a value of 1260/mm^3^ after the first dose. Overall in the two trials, 10 subjects were of African descent and their baseline median ANC (i.e., 2200/mm^3^) was significantly lower (Mann-Whitney p = 0.0007) compared to that of the 92 subjects who were not of African descent (i.e., 3555/mm^3^).

The cases of neutropenia were thoroughly discussed with investigators and the sponsor’s pharmacovigilance group, enrollment of new subjects was postponed, and study DSMB asked for assessment. On a cautionary basis, the DSMB recommended obtaining additional blood samples to monitor the ANC after vaccination and administration of additional vaccine doses only to subjects with ANC ≥1800/mm^3^ (i.e., the WHO threshold for neutropenia). Finally, they recommended discontinuation from any further vaccination in case of ANC <500/mm^3^ (i.e., grade 4 AE). These recommendations were endorsed by the sponsor and the respective ethical and regulatory authorities.

### Neutrophil counts in trials with licensed vaccines

**Fig 6:**
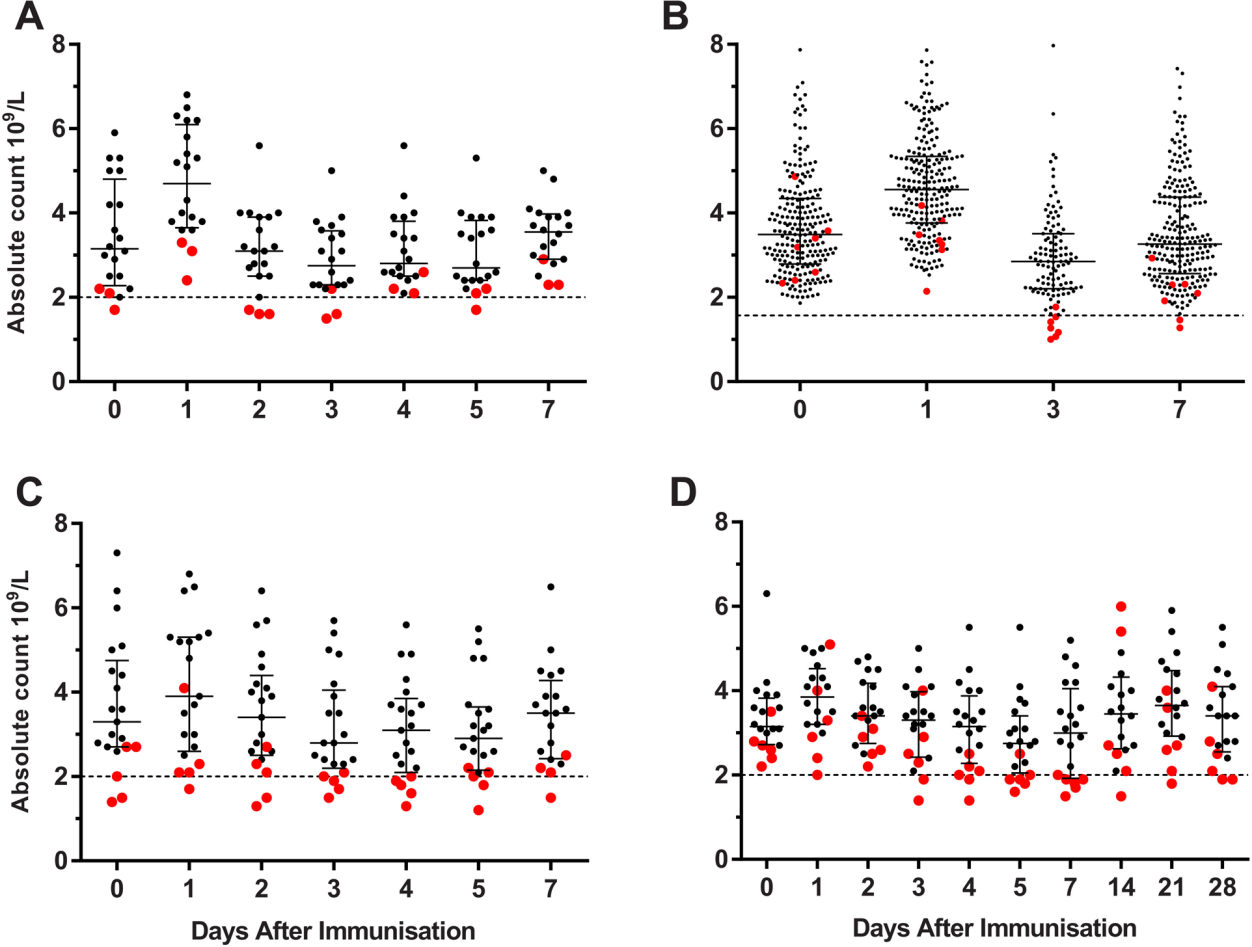
Absolute neutrophil count at different time points after vaccination on day 0. (A & B) adjuvanted seasonal TIV. (C) seasonal TIV. (D) attenuated live yellow fever vaccine. (A, C & D) conducted as inpatients. (B) conducted as outpatients. Red dots indicate subjects who had an absolute neutrophil count less than laboratory normal range on at least one time point after vaccination. Bars indicate median and interquartile range.

As shown in Fig 6, absolute neutropenia was not uncommon after immunization with live, adjuvanted and unadjuvanted licensed vaccines, but was generally mild (grade 1 and 2) and always asymptomatic. Participants with post-immunization neutropenia frequently had low pre-immunization counts or were at or below the interquartile range lower bounds prior to immunization—especially in the studies where participants were kept as inpatients and under controlled conditions.

### Literature review

The candidate vaccines, associated with neutropenia in the selected papers, included 14 live attenuated virus vaccines, 6 recombinant protein and peptide vaccines, 7 viral vectored vaccines, 1 conjugate vaccine, 1 DNA prime-Adenovirus boost vaccine, 1 canarypox virus-vectored prime- recombinant protein boost vaccine, 1 DNA vectored vaccine and 1 virus like particle (VLP) vaccine. Of the recombinant protein and peptide vaccines, 2 were formulated with aluminum adjuvants [13, 14], 1 with both alum and CPG, a synthetic oligodeoxynucleotide that acts as a TLR 9 agonist, [15, 16], 1 with MF59 [13], an oil in water adjuvant, and 1 was administered without an adjuvant [17]. A recombinant protein administered as a boost following priming with a virus vectored vaccine was also administered with an alum adjuvant [18]. Of all these vaccine candidates containing adjuvants, neutropenia and leukopenia were only linked to the TLR 9 agonist CPG, with the authors noting in both articles that a transient drop in leukocyte counts was expected following administration of this adjuvant and that this was probably a result of redistribution of white blood cells as a part of the immune response [15] [16]. More specifically, Ellis et al. noted a decrease in the neutrophil counts in all subjects receiving the vaccine candidate with the CPG adjuvant [15].

With regard to temporal associations, a total of 19 articles described the time to detection of neutropenia following vaccine administration [11, 12, 14, 15, 19–33]. This ranged from detection on the day of vaccine administration (day 0) in one article [31] to detection 4 weeks post vaccination in 3 articles [14, 22, 32]. The two instances of neutropenia reported at day 0 occurred before vaccination and normalized 2 and 10 days later, respectively [31]. The time to detection of neutropenia was dependent on the schedule of blood draws defined in the respective protocols. The majority of reported cases (14 of the 19 articles) were detected in the first 2 weeks after vaccination. The time from diagnosis to resolution of the neutropenia was reported in 11 articles [11, 14, 19, 23, 25, 28, 30, 31, 34–36]. In four instances the vaccine was not considered as the cause of neutropenia; more specifically, Dieye et al [19] reported a single case deemed to be unrelated to the vaccine candidate; Durbin et al [34] noted no statistical difference between the number of vaccinees and placebo recipients that suffered neutropenia with one case linked to infection with Influenza B; Kotloff et al [14] and Taalat et al [28] both diagnosed benign ethnic neutropenia (BEN) in patients with a history of low neutrophil counts prior to vaccination. The other 7 articles reported cases of neutropenia that were probably related to vaccine administration and that resolved between 2 and 21 days after the diagnosis of neutropenia had been made. In all cases, resolution of neutropenia was achieved without any complication.

Three articles described the baseline neutrophil counts, for all subject groups, and their mean absolute decline following vaccine administration. These articles described the safety profiles of 2 Dengue virus vaccine candidates (one live attenuated against Dengue 1 and one chimeric against Dengue 2) and were authored by Durbin et al between 2006 and 2011 [11, 20, 37]. All three articles noted that vaccinees developing neutropenia post vaccination had a lower baseline ANC than vaccinees that did not develop neutropenia, and this difference was statistically significant. With regard to the vaccine rDEN1Δ30 [11, 20], two trials revealed that vaccine recipients with a baseline ANC below 3500/mm^3^ and 3000/mm^3^ respectively were more likely than other vaccine recipients to suffer neutropenia post vaccination. As a matter of fact, vaccinated volunteers who became neutropenic had a statistically significant lower mean baseline ANC value (2475/mm^3^) than those who did not become neutropenic (3675/mm^3^) and those who received placebo (3687/mm^3^). Additionally, there was no difference in the mean absolute decline in the neutrophil count between vaccinees that developed neutropenia and those that did not, although this figure was significantly higher for vaccine recipients versus placebo recipients. Finally, all subjects who developed neutropenia in these trials recovered fully and receipt of the vaccine was not a statistically significant predictor of neutropenia, although, as noted by the authors, this was probably due to the small sample size [20]. Njuguna et al [33] noted a low mean baseline ANC of 1300/mm^3^ among the infants enrolled into their study. Although neutropenia was one of the most commonly reported adverse events in this trial, there was no statistically significant difference in the incidence of neutropenia among vaccinees versus the control group and no association was made by the authors between the vaccine and the neutropenia cases.

Severity of the neutropenia was reported in 30 of the 35 articles retrieved by our search. This was either classified as mild, moderate or severe or graded by number, that is, grade 1 to 4. The parameters used for classification differed among trials or was not reported, making it difficult to compare the severity of neutropenia observed across trials. Importantly, none of the articles reported any complications associated with the episodes of neutropenia and no cases of febrile neutropenia were recorded. Two articles reported only severe cases of neutropenia [17, 38]. In one of them, neutropenia was among the most commonly recorded laboratory adverse event abnormalities, however during their trial, they did not report the number of mild or moderate cases that they captured and did not report the parameters of their classification. Only one severe case (360/mm^3^) was reported in temporal association with an intercurrent episode of HIV infection acquired by the subject during the study period due to exposure in the community. (32). As a matter of fact, HIV is commonly associated with cytopenias of all major blood cell lines, including neutrophils.Ethnicity of subjects suffering from neutropenia following immunization was reported in 10 of the 35 articles retrieved by our search. Cunningham et al [13] noted that infants of African descent had lower absolute neutrophil counts at 6, 18 and 24 months compared to infants with other ethnic backgrounds. A statistically significant association between ethnicity and neutropenia was reported in this article, with black infants more likely to develop neutropenia after vaccination. In contrast, although 60% of enrolled participants in a Dengue vaccine trial were of African descent [11], there was no statistical association between neutropenia and ethnicity despite the fact that a high proportion of vaccinees (45%) developed neutropenia and an association was noted between this event and the vaccine. Four articles described cases of subjects of African descent diagnosed to have benign ethnic neutropenia who developed neutropenia following administration of the vaccine candidates [14, 28, 29, 39]. One subject continued to have recurrent episodes of neutropenia throughout the duration of the trial and his condition was noted as ongoing at the end of the trial. None of these subjects suffered any complications with their conditions.

With regard to patient factors, aside from ethnicity, Kitchener et al [24] described one case of neutropenia in a lady with irregular menses. Conversely, no association was described with regard to age or gender of the vaccinees.

Two articles described episodes of neutropenia following re-challenge with a second dose of the vaccine candidates. Durbin et al [11] noted that following the second dose of the Dengue 1 vaccine, 9% of the vaccinees developed neutropenia compared with 45% of the same vaccinees who developed neutropenia after the first dose. In addition to this, they classified all the cases following the second dose as mild compared to 22% of cases after the first dose that were classified as severe and 13% that were classified as moderate. Ellis et al [15] noted that all subjects receiving the CPG containing malaria vaccine candidate had a transient decrease in their neutrophil count after the first dose and 92% of them were noted to have a similar decline in neutrophil counts after the second dose.

Other factors noted to be associated with neutropenia in these vaccine trials included the use of cotrimoxazole and zidovudine for prophylaxis by infants born to HIV positive women as reported by Cunningham et al [13]. Edelman et al [21] noted an association between the level of reactogenicity of the candidate Dengue vaccine and the likelihood of developing neutropenia. Two articles reported cases of neutropenia occurring with viral infections. Watanaveeradej et al [31] noted two cases of neutropenia associated with dengue viremia, with one of these cases resulting from a confirmed wild type infection. Durbin and colleagues [34] reported a case of severe neutropenia associated with an Influenza B infection.

## Discussion

A review of published medical literature available on PubMed revealed that neutropenia had been captured as an adverse event in >30 phase I and II vaccine trials. Factors that were noted by the investigators to be associated with neutropenia include low baseline neutrophil counts prior to vaccination, ethnicity- with subjects of African descent being noted to be more likely to develop neutropenia, although this finding was not confirmed in one trial [5], use of the CPG adjuvant, concurrent use of antiretrovirals and antibiotics, concurrent viral infections. Cases where vaccination was associated with neutropenia were noted to have recurrence of neutropenia after re-challenge with subsequent doses, although at a lower frequency and severity compared to the first vaccine administration [11].

Limitations of the literature and potential biases: this study only searched references from PubMed, which were either classified as phase 1 or Phase 2 publication or contained a description as a phase 1/I/2/II clinical trials in the Medline searchable text (i.e. title, abstract or MESH headings). Furthermore, only those papers that were available to the authors as full text were considered. As such, this survey is not a comprehensive listing of all trials where neutropenia may have occurred and been reported.

On the other hand, the combination of a relatively non-specific first search, followed by full text searches using the QUOSA software led to many more papers of interest than was possible using PubMed alone. For example, using “AND neutrop*” as a search term with the original search to 21^st^ August 2015 returned 22 papers. Only 5 of these were included in our final set of papers and of the 22, there were no papers missed in the original procedure.

There is a significant potential bias in using these data to estimate the frequency with which neutropenia occurs in vaccine trials since the majority of phase I and phase II vaccine trial reports we accessed did not report neutrophil counts (704 of the 771 papers in the initial survey that did not involve cancer studies). Neutropenia was detected in at least one subject in nearly a half (30 of 67) of the papers identified in this initial survey that did describe neutrophil counts. This is likely to be an over-estimate of the frequency since in some cases, these studies may have included neutrophil counts because earlier studies suggested this may have been a risk factor (e.g. Dengue papers), or there may be studies were neutrophils were measured but this was not reported in the paper and this is less likely to occur if neutropenia was observed. Thus we caution use of these data to estimate the actual frequency of neutropenia in Phase 1 and Phase 2 vaccine trials in healthy subjects. Nevertheless, the results from the literature survey suggest that decrease in neutrophil counts with transient neutropenia is a common post-vaccination occurrence with a benign clinical outcome. The results from the trials reported in the current manuscript do confirm that neutropenia occurring after vaccination is normally transient, clinically asymptomatic and do not reveal any association with age and gender of vaccinees.

The ability to diagnose neutropenia as an adverse event is heavily dependent on the design of the clinical trial and the timing of safety blood draws after vaccination. The majority of cases reported in the literature were detected in the two weeks following vaccination and therefore clinical trial designs would only detect these events, which are generally asymptomatic, if the safety blood draws were carried out to coincide with this period.

No standard definition for neutropenia was used in the articles from our literature search and no standard definition or severity criteria as an adverse event following immunization, has been defined. In the future, investigators reporting safety results from vaccine trials might benefit from a standard definition, similar to those provided by the Brighton Collaboration for other adverse events following immunization, to allow for the use of a single severity score and allow for the comparison of events across trials.

None of the trials reported cases of febrile neutropenia or any complications associated with neutropenia. A majority of cases recovered without sequelae, with a single patient, diagnosed with benign ethnic neutropenia, being noted to have an ongoing condition at the end of the trial [14].

The results from the trials reported in this manuscript, which include clinical testing of a novel vaccine against Shigellosis, developed with the GMMA manufacturing technology, but also of licensed vaccines with a well-known good safety profile, support the key findings of the literature review. All reported cases of neutropenia, including the grade 2 and grade 3 cases associated with the *Shigella* vaccination and the several cases reported after immunization with the licensed vaccines were transient and clinically asymptomatic. Additionally, for the *Shigella* vaccine, most of the cases occurred in subjects who had low neutrophil counts at baseline and in subjects of African descent, known to be more likely to have lower absolute neutrophils counts (i.e., benign ethnic neutropenia) compared to other ethnicities [3–6].

For this latter reason, the use of clinical laboratory reference intervals derived from non-African populations to evaluate the safety profile of drugs or vaccines in clinical trials performed in Africa or in persons of African descent is suboptimal, excludes potential trial volunteers and makes safety assessments less tailored to the population of interest. To address these limitations and ultimately increase the scientific validity of African clinical trials, efforts have been made to establish clinical laboratory reference intervals for various hematology, immunology and biochemistry values among healthy African adults, typical of those who might join a clinical trial [46]. More specifically for ANC, the referenced cross sectional study, conducted at seven clinical centers in Rwanda, Uganda, Kenya and Zambia, established a consensus African study interval of 1000–5300/mm^3^ compared to a US based interval of 1800–7700 /mm^3^. Should the latter interval be used in a clinical trial as an exclusion criterion, approximately 10% of healthy volunteers of African descent would have to be excluded from the trial.

As suggested both by our scientific literature search and by the results of the clinical trials, conducted with vaccines under development or already licensed, neutropenia is a common asymptomatic and clinically benign post vaccination laboratory abnormality. Monitoring neutropenia and other potential abnormalities in the early phases of clinical development of new vaccines is important for a more complete characterization of the safety profile of these vaccines, however, in consideration of reported ethnical diversities, it should be recommended that whenever local reference intervals, established through validated assays, are available, they should be used as inclusion/exclusion criteria or as a tool for determination of thresholds to be adopted for classification of adverse events in a clinical trial.

## Supporting Information

S1 Checklist PRISMA Checklist. (PDF)

S2 Checklist CONSORT Checklist. (PDF)

S1 Dataset H03_01TP data. (XLSX)

S2 Dataset H03_02TP data. (XLSX)

S3 Dataset Data shown in Fig 6. (XLSX)

S1 Protocol H03_01TP study protocol. (PDF)

S2 Protocol H03_02TP study protocol. (PDF)

S3 Protocol CRC305C study protocol. (PDF)

S4 Protocol CRC305A study protocol. (PDF)

S5 Protocol Ghent study protocol. (PDF)
