# Supplementary material for: Neutropenia as an Adverse Event following Vaccination: Results from Randomized Clinical Trials in Healthy Adults and Systematic Review
Source: PLoS One. 2016 Aug 4;11(8):e0157385. doi: 10.1371/journal.pone.0157385 (PMC4974007; doi:10.1371/journal.pone.0157385)
Supplement: S2 Protocol — (PDF) [file pone.0157385.s007.pdf]

CLINICAL STUDY PROTOCOL H03_02TP

Version 1.0 of 04 October 2013

Amendment 1 of 28 November 2013
Version 2.0 of 02 December 2013

Amendment 2 of 13 January 2014
Version 3.0 of 28 January 2014

Amendment 3 of 30 apr 2014
Version 4.0 of 13 may 2014

Amendment 4 of 10 July 2014
Version 5.0 of 15 July 2014

Amendment 5 of 28 July 2014
Version 6.0 of 28 July 2014

Amendment 6 of 13 August 2014
Version 7.0 of 02 September 2014

EUDRACT No. 2013-003374-27

A Phase 1, randomized, placebo controlled, single center, dose escalation study to evaluate the safety and immunogenicity of 3 vaccinations with *Shigella sonnei* vaccine (1790GAHB) administered either by intradermal, intranasal or intramuscular route in healthy adults.

Property of Novartis Vaccines Institute for Global Health

Confidential

May not be used, divulged, published or otherwise disclosed without written consent of Novartis Vaccines Institute for Global Health

PROTOCOL SYNOPSIS H03_02TP VERSION 7.0

**Name of Sponsor**<br>Novartis Vaccines Institute for Global Health	**Protocol number:**<br>**H03_02TP**	**Health authority trial registration number(s):**<br>EUDRACT No. 2013-003374-27
**Title of Study:** A Phase 1, randomized, placebo controlled, single center, dose escalation study to evaluate the safety and immunogenicity of 3 vaccinations of *Shigella sonnei* vaccine (1790GAHB) administered either by intradermal, intranasal, or intramuscular route in healthy adults.		
**Study Period:** Each subject will be followed-up for approximately 6 months after the last vaccination (following the screening period, total study duration will be approximately 9 months for each subject).	**Clinical Phase:** Phase 1	
**Rationale:** Shigellosis remains a major health problem in developing countries with approximately 100 million cases per year mostly in children ≤5 years. No vaccine is currently available against shigellosis. Natural infection generates a protective immune response directed to the serotype-specific O antigen (OAg) of the lipopolysaccharide (LPS). Among the Shigella serotypes that are epidemiologically more relevant, *S. sonnei* and *S. flexneri* type 2 where selected for a prototype OAg conjugate vaccine that achieved 70% efficacy in children ≥3 years. However, no efficacy was observed in younger children and, thus, new approaches are needed. In addition, to achieve broad-spectrum protection against the 16 serotypes that are currently considered to be globally important, a multivalent OAg-vaccine will be most probably needed.<br><br>The proposed trial is aimed to address the safety and immunogenicity of a candidate vaccine against *S. sonnei*. The study is also the proof of concept for a new platform technology called Generalized Modules for Membrane Antigens (GMMA, also known as outer membrane vesicles), which may be also applicable for other vaccines against Gram-negative pathogens.<br><br>The candidate 1790GAHB vaccine is based on outer membrane particles that are naturally released from the *S. sonnei* during growth. The natural arrangement of the outer membrane is preserved during the release of GMMA and therefore GMMA allow an optimal exposure of the antigens of the outer membrane for recognition by the host immune system. NVGH has developed a cost-effective process to purify GMMA in large quantities from high density cultures of bacteria genetically modified to increase GMMA production and generate a LPS with low endotoxicity, suitable for use in humans. *S. sonnei* was chosen for developing a prototype Shigella vaccine based on the GMMA technology (1790GAHB) since *S. sonnei* is among the most common serotypes causing dysentery in humans. At the same time, the proposed Phase 1 trial will represent the proof of concept for the GMMA technology which might be used also for the		

**Name of Sponsor** Novartis Vaccines Institute for Global Health	**Protocol number: H03_02TP**	**Health authority trial registration number(s):** EUDRACT No. 2013-003374-27

development of other vaccines.

The proposed trial is aimed to investigate the safety and immunogenicity of 1790GAHB vaccine when administered at different dosages by different routes in healthy adults. In the murine challenge model intranasal (IN) vaccination was more effective than subcutaneous (SC) vaccination suggesting that a mucosal vaccination route might be beneficial. This is also in accordance with the current opinion that the most effective vaccination against enteric pathogens requires a mucosal vaccination route. Additionally, non-GLP studies in rabbits at NVGH support the use of OAg-GMMA by IN and intradermal (ID) vaccination routes, which are also suggested by the small size and high surface to volume ratio of GMMA. Finally, to have an IM evaluation in the same study where ID and IN are evaluated, a group of subjects will be vaccinated by the IM route using a dose of 5 µg. Thus, the proposed trial will investigate 3 different vaccination routes in humans.

The 1790GAHB vaccine proved to be highly immunogenic in mice where a SC dose as low as 1 µg was immunogenic. It is known from other vaccines (e.g. influenza vaccine) that the ID immunization triggers similar immune response as IM immunization at significantly lower dosages (with the Flu vaccine 20% of the IM dose when applied ID is as immunogenic as the full IM dose). Since this is the first time in man trial with a new vaccine, it was decided to start from 10% of the lowest IM dose (1 µg in the H03_01TP study) for the ID vaccination and therefore 0.1 µg has been selected as starting dose for ID administration. This dose, applied SC in mice, was still very immunogenic. For the IN administration in contrast, higher amounts of antigen than for IM are usually needed to trigger a good immune response. Therefore 5 µg has been selected as starting dose for IN administration. The highest dose proposed for IN administration is determined by the antigen concentration of the clinical lot (200 µg / mL) and by the maximum volume (200 µL) which can be efficiently administered into each nostril, resulting in a total dosage of 80 µg.

In the first part of this study and in another parallel study (H03_01TP) with the same vaccine there have been few subjects who have experienced a transient decrease of circulating neutrophils. This decrease was below the normal reference range, and in two cases (one in each study) it was classified as a severe adverse event, but not as a serious adverse event. Although this finding was not associated with any clinical illness, as recommended by the trial DSMB, it prompted the introduction of some urgent safety measures in this protocol to protect the safety of study subjects.

Objectives:

Primary Objective: to evaluate the safety profile of different dosages of NVGH 1790GAHB vaccine in adults, when administered intradermally, intranasally or intramuscularly

**Name of Sponsor** Novartis Vaccines Institute for Global Health	**Protocol number: H03_02TP**	**Health authority trial registration number(s):** EUDRACT No. 2013-003374-27
**Secondary Objective:** To evaluate the immunogenicity profile of different dosages of investigational NVGH 1790GAHB vaccine in adults when administered intradermally, intranasally, or intramuscularly at 28 days after 1st vaccination, 28 days after 2nd vaccination and 28 and 168 days after 3rd vaccination by measuring the anti-LPS serum IgG.<br><br>Exploratory Objectives:<br><br>To evaluate the mucosal immunity induced by different dosages of investigational 1790GAHB vaccine in adults when administered intradermally, intranasally, or intramuscularly in stool samples obtained at 28 days after 1st vaccination, 28 days after 2nd vaccination and 28 and 168 days after 3rd vaccination, by measuring the anti-LPS fecal secretory IgA (sIgA).<br><br>To determine in a subset of subjects (each group of cohort C) the frequency of circulating memory B cells specific for the OAg of the vaccine and its carrier (GMMA) before vaccination, at 28 days after 1st vaccination, at 28 days after 2nd vaccination and at 28 and 168 days after 3rd vaccination.<br><br>To determine in a subset of subjects (each group of cohort C) the frequency of circulating vaccine specific antibody secreting cells B cells (plasma blasts) at day 7 after the first vaccine dose.		
**Methodology:** This randomized, placebo controlled, single center, Phase 1 clinical trial is designed to evaluate the safety and immunogenicity of different dosages, administered via different vaccination routes, of the NVGH 1790GAHB vaccine in adults (18 to 45 years of age at enrollment). As no vaccine is currently available against shigellosis, the safety profile of 1790GAHB vaccine will be evaluated in comparison to that of a placebo, constituted by an aluminum hydroxide (Alhydrogel) suspension with the same concentration as for study vaccine dosages, in Tris buffered saline. The study will be observer-blind with respect to the assignment to treatment or placebo group while information concerning dosage applied to each group and route of administration will not be blinded due to the dose escalating design and the vaccinations procedures.<br><br>During the screening period before 1st vaccination (day -28 to randomization according to the study Times and Events Table), subjects providing informed consent will be screened for general health status and all results of the screening tests will be available before randomization. Approx. 25 mL blood draw will be obtained (for hematology, renal, bone and liver panels and HLA testing) as part of the screening. Subjects being screened for enrollment into cohort C after 10th July 2014 (date of amendment 4) with an absolute neutrophil count (ANC) less than $1.8x10^9$/L at screening will not be enrolled in the study. A specific test to exclude positivity for antibodies at baseline against *S. sonnei* will be performed as part of the screening on serum from approximately 2 ml of		

**Name of Sponsor** Novartis Vaccines Institute for Global Health	**Protocol number: H03_02TP**	**Health authority trial registration number(s):** EUDRACT No. 2013-003374-27

blood. Urine tests will also be performed. For all women of childbearing potential (defined as a pre-menopausal female capable of becoming pregnant. This does not include females who meet any of the following conditions: (1) menopause at least 2 years earlier, (2) tubal ligation at least 1 year earlier, or (3) total hysterectomy) the screening will include the evaluation of human chorionic gonadotropin (hCG) in blood to exclude pregnancy. A dipstick for the evaluation of human chorionic gonadotropin (hCG) in urines will then be performed at Visit 1 before randomization and will be repeated before each vaccination. No pharmacokinetic tests will be performed as evaluation of pharmacokinetic properties is only required for vaccines where new delivery systems are employed or when the vaccine contains novel adjuvants or excipients [EMEA/CHMP/VWP/164653/2005]. One additional blood draw of 10 mL for hematology, renal, bone and liver panels (see Table 2) will be obtained and urinalysis will be repeated at 7 days after 1st vaccination (Visit 2), 28 days after 2nd vaccination (Visit 4) and 28 days after 3rd vaccination (Visit 5). Blood for a complete blood count (CBC) will also be obtained 7 days after the 2nd and 3rd vaccinations (administered at visits 3 and 4 respectively), as well as 168 days after the 3rd vaccination to assess the ANC. All individuals with a neutropenia (ANC $<1.8x10^9$/L) will have the test repeated on a weekly basis until the neutropenia resolves (ANC $\geq 1.8x10^9$/L). If the neutrophil count does not rise to a value $\geq 1.8x10^9$/L by day 21 after vaccine administration, the subject will be discontinued from further vaccinations. In case the ANC is less than $0.5x10^9$/L after vaccination (AE Grade 4), the subject will have the test repeated on a weekly basis until the neutropenia resolves and will be discontinued from further vaccination if this takes place after the 1st or 2nd vaccination.

For all women of childbearing potential, the evaluation of human chorionic gonadotropin (hCG) in blood will be repeated at the end of the study. Clinically significant modifications in hematology, blood chemistry and urinalysis test values will be assessed by medical judgment based on interpretation of deviations from institution's normal values and recommendations from CBER FDA Guidance for industry: Toxicity Grading Scale for Healthy Adult and Adolescent Volunteers Enrolled in Preventive Vaccine Clinical Trials).

Subjects, who meet all inclusion criteria and none of the exclusion criteria, will be eligible for enrollment. Female subjects of child-bearing potential must use acceptable birth control measures (defined as hormonal [e.g., oral, injection, transdermal patch, implant, cervical ring], barrier [e.g., condom with spermicide or diaphragm with spermicide], intrauterine device [e.g., IUD], or monogamous relationship with partner who has been vasectomized for 6 months or more prior to the subject's study entry) during entire study participation. With a dose escalating approach, three antigen concentrations will be tested for both the IN and the ID vaccination routes. For the IM vaccination route, a single antigen concentration of 5 μg will be tested:

**Name of Sponsor** Novartis Vaccines Institute for Global Health	**Protocol number: H03_02TP**	**Health authority trial registration number(s):** EUDRACT No. 2013-003374-27

Route	Investigational Vaccine	No. of Subjects receiving treatment	No. of Subjects receiving placebo
**ID**	1790GAHB (0.1 μg*)	4	2
	1790GAHB (1 μg*)	6	2
	1790GAHB (10 μg*)	6	2
**IN**	1790GAHB (5 μg*)	4	2
	1790GAHB (20 μg*)	6	2
	1790GAHB (80 μg*)	6	2
**IM**	1790GAHB (5 μg*)	6	2
	Total	52	

*protein content

During the trial a total of 52 subjects will receive either vaccine or placebo. At day 1, eligible subjects will be randomly assigned, to receive three vaccinations, 4 weeks apart, with either 1790GAHB vaccine (at the lower dose for the IN and ID administration) or placebo. For the IM vaccinations, subjects will be randomly assigned to receive either 1790GAHB vaccine or placebo and will not be included into the dose escalation.
Groups will be recruited in 3 cohorts in the following sequence:

Cohort A) 0.1 μg ID, 5 μg IN
Cohort B) 1 μg ID and 20 μg IN
Cohort C) 10 μg ID and 80 μg IN and 5 μg IM

Specifically for IN and ID administration routes, an independent Data Safety Monitoring Board (DSMB) will receive a summary of all safety data (solicited local and systemic reactions, unsolicited adverse events and SAE) and listings of clinically significant modifications in hematology, blood chemistry and urinalysis test values obtained during one week follow-up post-first vaccination with the lower dosage. Based on evaluation of the safety data, the DSMB will make a recommendation, as to whether the next cohort should be vaccinated with the higher antigen concentration or not. Same approach will be followed until all cohorts have been enrolled. Independent dose escalation recommendations should be made for IN and ID vaccination routes.

Each randomized subject will have 20 mL of blood drawn and stool samples collected before and 28 days after 1st vaccination, 28 days after 2nd vaccination, and 28 and 168 days after 3rd vaccination for immunological studies (see Table 1 - Time and Events Table). Additionally, all subjects from Cohort C (N=24) will have an additional 32 mL of blood drawn before, 7 and 28 days after 1st vaccination, 28 days after 2nd vaccination, and 28 and 168 days after 3rd vaccination for cellular mediated immunity testing (exploratory objectives).

Subjects will be observed at the site for approximately 2 hours after each vaccination.

**Name of Sponsor** Novartis Vaccines Institute for Global Health	**Protocol number: H03_02TP**	**Health authority trial registration number(s):** EUDRACT No. 2013-003374-27

Diary cards will be used to collect all solicited, unsolicited adverse events, and medications/vaccinations given during 7 days (inclusive) following each vaccination. A reminder phone call will be performed by the site staff to the subjects 2 and 6 days following each vaccination to remind that the diary card should be completed (no update on the status of the subject's health will be solicited during these phone calls that are not intended for safety data collection). Seven days following 1st vaccination, a clinical visit will be performed at the study site and all information recorded in the diary card will be reported on e-CRF and source data in order to document all safety data occurred during the one week follow-up post-first vaccination. The source data will be reconciled with information in diary card brought in by the subject at Visit 3. After day 7 following 1st vaccination, only unsolicited adverse events, solicited reactions that continue beyond day 7, and related medications will be collected in the diary card until the time of return to the clinic for the 2nd vaccination. The same process will apply following 2nd and 3rd vaccination. All serious adverse events (SAEs), all medications given to treat SAEs, all new onset of chronic disease, all AEs leading to vaccine/study withdrawal, and all adverse events of special interest (AESIs) will be collected for the entire study. These data will be captured through the diary card, by interview of the subject and by review of available medical records.

A summary of the main medical and safety data to be collected into the e-CRF during the study is provided in Table 3 - Medical and Safety Assessments to be reported into CRF.

Number of Subjects planned: Screening phase will last until 52 subjects are randomized. Therefore overall a total of 52 subjects are planned for enrollment into the study to have 52 vaccinated. Subjects withdrawn after 1st vaccination or lost to follow up will not be replaced. Sample size is not driven by statistical calculation.

Subject Population: The study population will consist of healthy male and female adult volunteers aged 18 to 45 years.

Subject Characteristics and Criteria for Inclusion and Exclusion:

Inclusion Criteria

In order to participate in this study, all subjects must meet ALL of the inclusion criteria described.

1. Males and females of age ≥18 years to ≤45 years.
2. Individuals who, after the nature of the study have been explained to them, have given written consent according to local regulatory requirements.
3. Individuals in good health as determined by the outcome of medical history, physical examination, hematology, renal, bone and liver panels (including negative for agglutination testing of S. sonnei), urinalysis and clinical judgment of the

**Name of Sponsor** Novartis Vaccines Institute for Global Health	**Protocol number: H03_02TP**	**Health authority trial registration number(s):** EUDRACT No. 2013-003374-27

investigator.

4. If women of childbearing potential, have a negative pregnancy test prior to study vaccination and willingness to use acceptable contraceptive measures for the entire study duration.
5. Individuals available for follow-up for the duration of the study.
6. Individuals registered with a general practitioner.

Exclusion Criteria
In order to participate in this study, all subjects must meet NONE of the exclusion criteria described.

1. Individuals with a history of recurrent wheezing, asthma, respiratory allergies, allergic rhinitis, nasal surgery or significant nasal abnormalities (e.g. polyps), and Bell's palsy. Presence of nasal piercings. Symptoms of upper respiratory tract infection within 3 days of intended study vaccination is a temporary exclusion criterion.
2. Individuals unwilling to abstain from medications or other agents that are applied via the nasal route from 24 hours prior to each nasal dosing through to the safety assessment 1 week later.
3. Individuals with behavioral or cognitive impairment or psychiatric disease that, in the opinion of the investigator, may interfere with the subject's ability to participate in the study.
4. Individuals with any progressive or severe neurological disorder, seizure disorder or Guillain-Barré syndrome.
5. Individuals who are not able to understand and to follow all required study procedures for the whole period of the study.
6. Individuals with history of any illness that, in the opinion of the investigator, might interfere with the results of the study or pose additional risk to the subjects due to participation in the study.
7. Individuals with human leukocyte antigen (HLA) -B27 positive and/or with history of reactive arthritis
8. Individuals with known HIV, HBV and HCV infection or HIV related disease, with history of an autoimmune disorder or any other known or suspected impairment /alteration of the immune system, or under immunosuppressive therapy including use of systemic corticosteroids or chronic use of inhaled high-potency corticosteroids within the previous 30 days, or were in chemotherapy treatment within the past 6 months.
9. Individuals with a known bleeding diathesis, or any condition that may be associated

**Name of Sponsor** Novartis Vaccines Institute for Global Health	**Protocol number: H03_02TP**	**Health authority trial registration number(s):** EUDRACT No. 2013-003374-27

with a prolonged bleeding time.

10. Individuals with any serious chronic or progressive disease according to judgment of the investigator (e.g., neoplasm, insulin dependent diabetes, cardiac, renal or hepatic disease).
11. Individuals who have any malignancy or lymphoproliferative disorder.
12. Individuals with history of allergy to vaccine components.
13. Individuals participating in any clinical trial with another investigational product 90 days prior to first study visit or intent to participate in another clinical study at any time during the conduct of this study.
14. Individuals who received any other vaccines within 4 weeks prior to enrollment in this study or who are planning to receive any vaccine within the entire study duration except influenza vaccination, which is not allowed within the period included between 4 weeks before 1st vaccination and 4 weeks after 3rd vaccination
15. Individuals who have received blood, blood products and/or plasma derivatives including parenteral immunoglobulin preparations in the past 12 weeks before randomization.
16. Individuals who are part of study personnel or close family members to the personnel conducting this study or employees of the clinical trial site institution.
17. Individuals with body temperature > 38.0 degrees Celsius within 3 days of intended study vaccination.
18. BMI > 30 kg/m2.
19. Individuals with history of substance or alcohol abuse within the past 2 years.
20. Women who are pregnant or breast-feeding or of childbearing age who have not used or do not plan to use acceptable birth control measures, for the duration of the study.
21. Females with history of stillbirth, neonatal loss (history of planned abortion in not an exclusion), or previous infant with anomaly.
22. Individuals who have a previously ascertained or suspected disease caused by S. sonnei or positive *S. sonnei* serology at screening
23. Individuals who have had household contact with/and or intimate exposure to an individual with laboratory confirmed *S. sonnei*
24. Any condition, which, in the opinion of the investigator may pose an increased and unreasonable safety risk to the subject if participating to the present study.
25. Individuals to be enrolled into Cohort C after 10th July 2014 (date of amendment 4) with a neutrophil count lower than 1.8×10^9/L at screening.

**Name of Sponsor** Novartis Vaccines Institute for Global Health	**Protocol number: H03_02TP**	**Health authority trial registration number(s):** EUDRACT No. 2013-003374-27

Vaccines:

NVGH S. sonnei (1790GAHB) vaccine

The test treatment is the NVGH *S. sonnei* vaccine. The vaccine consists of *S. sonnei* 1790-GMMA (200 µg/mL, measured by protein content) adsorbed to Alhydrogel, (0.7 mg Al^{3+}/mL) in Tris-buffered saline. The vaccine is available in single dose vials with 0.7 mL of injectable solution containing 140 µg of GMMA (as protein content) adsorbed onto 0.49 mg of Al^{3+}. The vaccine is available as a liquid formulation and does not contain any preservative.

The vaccine is used at different antigen dosages (that will be obtained by bed-side mixing) and different volumes, depending on the vaccination route, as follows:

Intradermal administration

For the intradermal injections, a syringe will be used. The volume administered will be 0.05 mL.

ID 0.1 µg: Following dilution, each dose contains 0.1 µg of GMMA total protein and 0.035 mg of Al^{3+}.

ID 1 µg: Following dilution, each dose contains 1 µg of GMMA total protein and 0.035 mg of Al^{3+}.

ID 10 µg: Each dose contains 10 µg of GMMA total protein and 0.035 mg of Al^{3+}.

Intranasal administration

For the IN injections, a mucosal atomization device will be used for dose spraying (manufactured by Wolfe Tory Medical, Inc. Utah and distributed by Cavendish Scott Ltd, England). The total volume administered, preferentially half in each nostril, will be 0.4 mL.

IN 5 µg: Following dilution, each dose contains 5 µg of GMMA total protein and 0.28 mg of Al^{3+}.

IN 20 µg: Following dilution, each dose contains 20 µg of GMMA total protein and 0.28 mg of Al^{3+}.

IN 80 µg: Each dose contains 80 µg of GMMA total protein and 0.28 mg of Al^{3+}.

Intramuscular administration

IM 5 µg: Each 0.5 mL dose contains 5 µg of GMMA total protein and 0.35 mg of Al^{3+}.

Three vaccinations will be administered, 28 days apart. IM and ID vaccinations will be administered preferentially in the deltoid of the non-dominant arm and IN vaccinations in both nostrils. Bed-side mixing instructions will be provided to the investigator and will be located in the investigator site file. Refer to Protocol Section 5.3 for additional

**Name of Sponsor** Novartis Vaccines Institute for Global Health	**Protocol number: H03_02TP**	**Health authority trial registration number(s):** EUDRACT No. 2013-003374-27

instructions.

Control treatment (Placebo)
The control treatment is a placebo. The placebo is composed of Alhdyrogel (0.7 mg Al^{3+}/mL) in Tris–buffered saline. The placebo is available in single dose vials with 0.7 ml of injectable solution containing 0.49 mg of Al^{3+}. Three placebo doses, 28 days apart, will be administered as follows:

1. 0.05 mL intradermally, containing 0.035 mg of Al^{3+}
2. 0.4 mL intranasally, containing 0.28 mg of Al3+
3. 0.5 mL intramuscularly, containing 0.35 mg of Al^{3+}

No concomitant vaccines or treatments will be used as part of study procedures.

Immunogenicity Endpoints:
The measures of the primary immunogenicity outcome, (i.e., the anti-LPS *S. sonnei* serum IgG), will include:

a. IgG Geometric mean concentrations (GMCs) pre-vaccination (day 1), 28 days after 1st vaccination, 28 days after 2nd vaccination, 28 and 168 days after 3rd vaccination as determined by Enzyme-linked Immunosorbent Assay (ELISA), and applicable geometric mean ratios between post- and pre-vaccination samples.
b. Number of subjects with seroresponse for anti- LPS *S. sonnei* at 28 days after 1st vaccination, 28 days after 2nd vaccination, and 28 and 168 days after 3rd vaccination

Seroresponse is aimed to define a significant increase in post vaccination samples based on the biological performance of this specific serology assay and it is defined as:

If half of the baseline value is greater than 25 EU then an increase of at least 50% in the post-vaccination sample as compared to baseline [i.e. ((Post-vac minus baseline)/baseline)100% ≥ 50%]

If half of the baseline value is less or equal to 25 EU then an increase of at least 25 EU in the post-vaccination sample as compared to baseline [i.e. (post-vac minus baseline) ≥ 25 EU]

c. Number of subjects with high seroresponse for anti-LPS *S. sonnei* at 28 days after 1st vaccination, 28 days after 2nd vaccination, and 28 and 168 days after 3rd vaccination

High seroresponse is defined as a post vaccination titer ≥ X anti-LPS serum IgG units in the Novartis ELISA that correspond to a titer of 1:800 in the ELISA method used by Cohen et al. (1989 J. Clin. Microbiol. 27:162). To determine the value for 'X' the Novartis anti-LPS ELISA will be calibrated against the Cohen ELISA.

Other assays might be done to further characterize the immune response to the study vaccine.

**Name of Sponsor** Novartis Vaccines Institute for Global Health	**Protocol number: H03_02TP**	**Health authority trial registration number(s):** EUDRACT No. 2013-003374-27

The serologic assays on clinical samples will be performed at Novartis Vaccines, Clinical Serology Laboratory, Marburg, Germany or a delegated laboratory.

Safety Endpoints:

The measures of safety will include:

- Numbers of subjects with deviations from normal values of hematology, renal, bone and liver panels and urinalysis after vaccination.

- Number of subjects with solicited local and systemic adverse reactions during 7 days following each vaccination. Solicited local reactions following IM vaccination include erythema, induration and pain at injection site; solicited local reactions following ID vaccination include erythema, induration, pain at injection site and swelling; solicited local reactions following IN vaccination include facial edema, nasal pain and rhinorrhea. Solicited systemic reactions include headache, arthralgia, chills, fatigue, malaise, myalgia, and fever as measured orally.

- Number of subjects with reported unsolicited adverse events during 28 days following each vaccination.

- Number of subjects with reported SAEs throughout the study duration

- Number of subjects with reported reactive arthritis (AEs of special interest (AESIs). ReA is defined as non-purulent joint inflammation that develops in response to an infection in another part of the body. Since the inflammation is triggered by a previous condition, it is termed “reactive”. Intestinal pathogens that have been associated with ReA include Campylobacter, Salmonella, Yersinia, *Clostridium difficile*, and Shigella. If reactive arthritis is caused by an auto immune response, there is at least a possibility that it could be initiated by vaccination of susceptible people (i.e. HLA-B27 positive individuals) with the 1790GAHB vaccine.

Safety data will be summarized by vaccination, route of administration and dosages/placebo groups.

Exploratory Immunogenicity Endpoints:

The measures of the exploratory immunogenicity outcome, (i.e., the anti-LPS fecal sIgA), will include:

Fecal sIgA GMCs pre-vaccination (day 1), 28 days after 1st vaccination, 28 days after 2nd vaccination, 28 and 168 days after 3rd vaccination, as determined by ELISA, and applicable geometric mean ratios between post- and pre-vaccination samples

**Name of Sponsor** Novartis Vaccines Institute for Global Health	**Protocol number: H03_02TP**	**Health authority trial registration number(s):** EUDRACT No. 2013-003374-27
Fecal sIgA will be assessed in the stool specimens of at least one cohort.<br><br>Frequencies and fold increases of memory B cells specific for the OAg of the vaccine and its carrier (GMMA) and antibody secreting cells B cells (plasma blasts) at relevant time points, will be analyzed descriptively.		
**Statistical Analysis of Primary Objective(s):** This Phase I safety and immunogenicity trial is aimed to descriptively evaluate the safety and immunogenicity profiles of the study vaccines. No specific hypotheses are tested in this trial.		
**Interim Analysis:** After all subjects in each cohort have completed enrolment and vaccinations and post vaccination sera and stools results (one month after dose 1, 2 and 3) are available, a group- unblinded preliminary immunogenicity analysis and a blinded interim safety analysis may be performed. Individual subject results from preliminary analyses will not be made available to site and sponsor personnel until the end of the study. Further details regarding the interim analysis are contained in section 7.5 of the protocol.		
**Stopping rules:** The occurrence of two cases of Grade 3 or Grade 4 neutropenia, or of febrile neutropenia, in one study cohort will result in study hold, unblinding of data, discussion of results with safety management team (SMT) and the product stewardship board (PSB), and final decision made in consultation with DSMB and authorities.		
**Data Monitoring Committee:** An independent, DSMB will be established to recommend if to proceed with the clinical testing of progressively higher dosages for IN and ID administration routes.<br><br>In case no related/suspected SAE and no at least possibly related solicited and unsolicited severe/grade 3 AE occurred during the one week follow-up post-first vaccination with lower dose, DSMB will recommend that the second IN and ID cohorts will be vaccinated either with *S. sonnei* 1790GAHB next dose or placebo. With the same approach subsequent IN and ID cohorts will be vaccinated with progressively higher antigen dosages.<br><br>In case of occurrence of related/suspected SAE and/or at least possibly related solicited and unsolicited severe/grade 3 AEs, DSMB will evaluate all safety data and will provide recommendation on whether the study should be halted or continued as planned.<br><br>The composition of DSMB and the details of all relevant procedures will be documented in the DSMB Charter.		

Table 1. Times and Events Table

Study Periods	**Screening**	**Treatment**										**Follow-up**
**Visit Type**	**Clinic**	**Clinic**	**Reminder Call**	**Clinic**	**Clinic**	**Clinic**	**Reminder Call**	**Clinic**	**Clinic**	**Reminder Call**	**Clinic**	**Clinic**
**Visit Number**	n/a	1	n/a	2	3	3.1	n/a	4	4.1	n/a	5	6
**Study Day***	**n/a**	**1**	**V1+2<br>V1+6**	**V1+7**	**V1+28**	**V3+7**	**V3+2<br>V3+6**	**V3+28**	**V4+7**	**V4+2<br>V4+6**	**V4+28**	**V4+168**
**Study Visit Time Window (min -max)**	**-28/ DAY 1**	**0**	**+2**	**+1**	**-3/+4**	**+1**	**+2**	**-3/+4**	**+1**	**+2**	**-3/+4**	**-15/+15**
**Informed Consent[a]**	X											
**Randomisation**		X										
**Medical History**	X											
**Limited Physical Exam/ Symptom-Directed Physical Exam[b]**	X	X			X			X				
**Exclusion/Inclusion Criteria[c]**	X	X		X	X			X			X	X
**Pregnancy Test (childbearing potential women only) [approx. 4 mL blood] [d]**	X (blood)	X (urine)			X (urine)			X (urine)				X (blood)
**Urine test**	X			X				X			X	
**HLA Laboratory Blood draw [approx. 4 mL whole blood][d]**	X											
**Safety Laboratory Blood Draw [approx. 10 mL whole blood][d]**	X			X				X			X	
**HIV, HBV and HCV Blood draw [approx. 4 mL whole blood][d]**	X											
**Serology Blood draw [approx: 20 mL whole blood][d]**		X			X			X			X	X
**Stools samples collected**		X			X			X			X	X
**Study Vaccine Administered**		X			X			X				

Study Periods	Screening	Treatment										Follow-up
**Visit Type**	**Clinic**	**Clinic**	**Reminder Call**	**Clinic**	**Clinic**	**Clinic**	**Reminder Call**	**Clinic**	**Clinic**	**Reminder Call**	**Clinic**	**Clinic**
**Visit Number**	n/a	1	n/a	2	3	3.1	n/a	4	4.1	n/a	5	6
**Study Day***	**n/a**	**1**	**V1+2 V1+6**	**V1+7**	**V1+28**	**V3+7**	**V3+2 V3+6**	**V3+28**	**V4+7**	**V4+2 V4+6**	**V4+28**	**V4+168**
**Study Visit Time Window (min -max)**	**-28/ DAY 1**	**0**	**+2**	**+1**	**-3/+4**	**+1**	**+2**	**-3/+4**	**+1**	**+2**	**-3/+4**	**-15/+15**
**Post Injection Assessment** [e]		X			X			X				
**Indicators of Reactogenicity**[f]		X		X				X			X	
**Training [Diary Card]** [g]		X		X	X			X				
**Telephone contact for Reminder to Complete Diary Card**			X				X			X		
**Diary Card Reviewed and collected**[h]				X	X			X			X	
**Assess AEs**[i]		X		X	X			X			X	
**Assess SAEs and AEs Leading to Withdrawal From Study**[j]		X		X	X			X			X	X
**Concomitant Medications/vaccines**[k]	X	X		X	X			X			X	X
**Blood sample for cellular mediated immunity testing**[m]		X		X	X			X			X	X
**Agglutination test**[;l]	X											
**Study Termination**												X
**Complete blood count to assess neutrophil count; to be repeated on a weekly basis until resolution if ANC <1.8x10^9/L.**				X		X			X			X

*** Study days should be calculated based on the actual date of the previous visit (as to comply with requested Study Visit Time Window)**

a. Informed Consent to be obtained before any study procedure
b. Physical examination must be performed by a qualified health professional in accordance with local regulations and licensing requirements designated within the Site Responsibility Delegation Log. See section 6.2 for components of physical examination by visit.

c. Local and Systemic Adverse events, Body Temperature. Compliance with Exclusion/Inclusion criteria should be verified during entire study duration
d. Approximately blood drawn refers to the volume drawn at each specified visit. See section 3.5.1 for greater detail regarding blood sampling volumes
e. A post-injection local and system adverse event and body temperature measurement will be performed approximately after 30 and 120 minutes after each vaccination during the clinic visit.
f. Beginning in the evening (approximately 6 hours) following study vaccine administration, and daily thereafter through the following 6 days, solicited local and systemic adverse events including other reactions (i.e. body temperature measurements and use of analgesics/antipyretics) will be reported daily by the subject on a diary card.
g. Subjects will receive instruction on diary card completion. A diary card will be dispensed at these visits. See section 3.2.5.3 for more detail.
h. Review of safety data captured on Diary Cards will be completed at these visits. Subjects will be asked to return to the study clinic with the Diary Card completed. See section 3.2.5.5 for greater detail about diary card review.
i. All unsolicited adverse events will be captured through 28 days following each vaccination. Please see sections 3.2.5.5 and 6.6 for greater detail regarding methods for unsolicited safety data collection.
j. SAEs and AEs leading to study or vaccine withdrawal will be collected through entire study duration. Please see section 6.6 for greater detail regarding methods for SAE and AEs leading to study or vaccine withdrawal collection.
k. Collect concomitant medications and vaccination history according to the study procedures outlined throughout section 3.2.5 and 5.4. Any subject who terminates the study during the 28 days post-Vaccination period (prior to following study visit) is recommended to undergo study-related procedures required at the next clinic visitl.
l. Agglutination test for S. sonnei, to be done, at screening only, on serum from approximately 2ml bloodm Blood sample for cellular mediated immunity testing to be collected in cohort C only

Table 2. Hematological, Haematochemical Blood Tests and Urinalysis Table

**HEMATOLOGY**	
White Blood Cells (WBC)	10^9/l
Red Blood Cells (RBC)	10^{12}/l
Haemoglobin	g/l
Haematocrit	PCV
Platelets	10^9/l
Eosinophils	10^9/l
Basophils	10^9/l
Neutrophils	10^9/l
Monocytes	10^9/l
Lymphocytes	10^9/l
Prothrombin time*	secs
**CLINICAL CHEMISTRY**	
Total bilirubin	µmol/l
Aspartic Aminotransferase (ASAT/GOT)	IU/l
Alanine Aminotransferase (ALAT/GPT)	IU/l
γ-Glutamyl Transpeptidase (γ-GT)	IU/l
Lactic Dehydrogenase (LDH)	IU/l
Alkaline Phosphatase (AP)	IU/l
Total Proteins*	g/l
Glucose (fasting)	mmol/l
BUN	mmol/l
Creatinine	µmol/l
Sodium	mmol/l
Potassium	mmol/l
**VIROLOGY**	
HbsAg*	
Hepatitis C antibodies*	
HIV antibodies*	
**PROTOCOL SPECIFIC TEST**	
HLA–B27 test*	
Agglutination of *S. sonnei**	

*performed at the screening only – not repeated on laboratory evaluation performed at Visit 2, Visit 4 and Visit 5

**Urinalysis DIPSTICK**
Glucose
Proteins
pH
Ketones
Nitrites
Blood
**MICROSCOPIC TEST on urine**<br>(performed only if dipstick shows clinically significant deviations from normal values)
Leucocytes (WBC)

**PREGNANCY TEST**
**URINE Pregnancy test** - Human chorionic gonadotropin (hCG)**
**BLOOD TEST** - Human chorionic gonadotropin (hCG)***
Erythrocytes (RBC)
Epithelial Cells
Casts
Crystals
Bacteria

**performed at Visit 1, Visit 3 and Visit 4

***performed at the screening and Visit 6

Table 3: Medical and Safety Assessments to be reported into CRF

**Screening (-28 days before Visit 1)**	**Medical History:**<br>Any significant past diagnosis including illness, injuries, hospitalizations, major surgeries, or other significant medical conditions which may impair the assessment of immunogenicity or safety of the study vaccine.
	**Physical Examination (including vital signs)::**<br>Including the assessment of respiratory system (respiratory rate) and cardiovascular system (systolic/diastolic blood pressureheart rate and pulse rate).
	**Medications:**<br>All medications, vaccines and blood products taken or received by the subject within 28 days prior to the enrollment.
	**Pregnancy test:**<br>For all women of child-bearing potential, evaluation of human chorionic gonadotropin (hCG) in blood to exclude pregnancy
	**Laboratory Assessments:**<br>According to study protocol, all data resulting from the hematological, haematochemical blood tests and urinalysis.
**Before Randomization (V1) and before each vaccination (V1, V3, V4)**	**Physical Examination (including vital signs):**<br>Including the assessment of respiratory system (respiratory rate) and cardiovascular system (systolic/diastolic blood pressure heart rate and pulse rate).<br>Review and verification of results of screening laboratory assessments.
	**Medications:**<br>All concomitant medications (including vaccines) taken/administered to the subject (excluding vitamins and minerals).<br>Antipyretics and/or analgesics taken during 24 hours prior to each vaccination and the reason for their administration.
	**Pregnancy test:**<br>For all women of child-bearing potential, a urine Pregnancy test for the evaluation of the human chorionic gonadotropin (hCG) to exclude pregnancy.
**All study (including follow-up)**	**Adverse Events and Serious Adverse Event:**<br>AEs leading to study withdrawal, all SAEs and all Adverse Events of special Interest (reactive arthritis according the study protocol).<br>AEs will be monitored until resolution or stabilization. If the AE becomes chronic, it will be monitored until a cause is identified. If an AE is unresolved at the conclusion of the study, a clinical assessment will be made by the investigator and medical monitor whether continued follow-up of the AE is warranted.
	**Laboratory Assessments:**<br>According to study protocol, all data resulting from the hematological, haematochemical blood tests and urinalysis.
	**Medications:**<br>Any medication or other therapeutic measure used to treat the (S)AE
	**New diagnosis of disease**<br>All new onsets of chronic disease (NOCD)
**Approximately 30 minutes and**	**Vital signs:**<br>Assessment of respiratory system (respiratory rate) and cardiovascular system (systolic/diastolic blood pressure, heart rate and pulse rate).

**2 hours after each vaccination**	**Immediate reactions:**<br>Signs and symptoms of anaphylaxis
	**Local reactions:**<br>Erythema, Induration, Pain at the injection site (IM route)<br>Erythema, induration, pain and swelling at injection site (ID route)<br>Facial edema, nasal pain and rhinorrhea (IN route)
	**Systemic reactions:**<br>Headache, Arthralgia, Chills, Fatigue, Malaise, Myalgia
	**Body temperature:**<br>Measured orally (fever is defined as oral temperature ≥ 38°C)
	**Medications:**<br>Use/administration of antipyretics/ analgesics
**During 28 days after each vaccination**	**All Adverse Events:**<br>If a solicited local or systemic reaction continues beyond day 7 after vaccination, it will be also recorded as an Adverse Event.<br>All AEs will be monitored until resolution or stabilization. If the AE becomes chronic, it will be monitored until a cause is identified. If an AE is unresolved at the conclusion of the study, a clinical assessment will be made by the investigator and medical monitor whether continued follow-up of the AE is warranted.
	**Medications:**<br>All medications used to treat AEs and all vaccinations.

TABLE OF CONTENTS

PROTOCOL SYNOPSIS H03_02TP VERSION 7.0 2
TABLE OF CONTENTS 21
LIST OF ABBREVIATIONS 25
1.0 BACKGROUND AND RATIONALE 27
2.0 OBJECTIVES 30
2.1 Primary Objective(s) 30
2.2 Secondary Objectives 30
3.0 STUDY DESIGN AND INVESTIGATIONAL PLAN 31
3.1 Overview of Study Design 31
3.1.1 Study Period 33
3.2 Study Visit Procedures 33
3.2.1 Informed Consent/Assent 35
3.2.2 Screening Procedures 35
3.2.3 Enrollment 36
3.2.4 Randomization 37
3.2.5 Visit Procedures 37
3.2.5.1 Pre-vaccination Procedures 37
3.2.5.2 Vaccination Procedures 38
3.2.5.3 Post-vaccination Procedures 38
3.2.5.4 Clinic Visits After 1st Vaccination 39
3.2.5.5 Reminder Telephone Calls 40
3.2.5.6 Safety Calls 40
3.2.5.7 “For cause” Visits 40
3.2.5.8 Termination Visits 40
3.3 Blinding Procedures 41
3.4 Data Collection 41
3.4.1 Data Collected From Subjects 41
3.4.2 Electronic Case Report Forms 42
3.5 Laboratory Assessments 43
3.5.1 Processing, Labeling and Storage of Serum Samples for Serology 43
3.5.2 Processing, Labeling and Storage of Stools Samples for Serology 43
3.5.3 Pregnancy Testing 43

3.5.4 Safety Laboratory Assessments 44
3.5.5 Cell Mediated Immunity Assessments 45
3.5.6 Culture/PCR/Genotyping Assessments 45
3.6 Stopping/Pausing Guidelines 45
3.7 Premature Withdrawal and Early Study Termination 45
3.8 Early Termination Visit 48
4.0 SELECTION OF STUDY POPULATION 49
4.1 Inclusion Criteria 49
4.2 Exclusion Criteria 49
4.3 Criteria for Delay of Vaccination and/or Blood Sampling 51
4.4 Criteria for Repeat Vaccination in the Study 51
5.0 TREATMENT OF SUBJECTS 52
5.1 Study Vaccine(s) 52
5.2 Non-Study Vaccines 53
5.3 Vaccines Preparation and Administration 53
5.4 Prior and Concomitant Medications and Vaccines 54
5.5 Vaccine Supply, Labeling, Storage, and Tracking 54
6.0 MEASUREMENTS 57
6.1 Appropriateness of Measurements 57
6.2 Demographics, Medical History and Physical Examination 57
6.3 Immunogenicity Measurements 58
6.4 Efficacy Measurements 58
6.5 Solicited Safety Measurements 58
6.6 Unsolicited Safety Measurements 60
6.6.1 Adverse Events 60
6.6.1.1 Adverse Events of Special Interest 61
6.6.2 Serious Adverse Events 62
6.6.3 Methods for Assessing and Recording AEs and SAEs 63
6.6.4 Pregnancies 64
6.7 Safety Laboratory Measurements 64
6.8 Other Measurements 65
6.9 Data Safety and Monitoring Board 65
7.0 ENDPOINTS AND STATISTICAL ANALYSES 66

7.1 Endpoints 66
7.1.1 Primary Endpoint(s) 66
7.1.2 Secondary Immunogenicity Endpoints 66
7.1.3 Secondary Efficacy Endpoints 67
7.1.4 Safety Endpoints 67
7.1.5 Other Endpoints 67
7.1.6 Exploratory Endpoints 67
7.2 Success Criteria 67
7.2.1 Success Criteria for Primary Objectives 67
7.2.2 Success Criteria for Secondary Immunogenicity Objectives 67
7.2.3 Success Criteria for Secondary Efficacy Objectives 67
7.2.4 Success Criteria for Safety Objectives 67
7.3 Analysis Sets 68
7.3.1 All Enrolled Set 68
7.3.2 Exposed Set 68
7.3.3 Full Analysis Set (FAS) Efficacy/Immunogenicity Set 68
7.3.4 Per Protocol (PP) Population, Efficacy/Immunogenicity Set 68
7.3.5 Safety Set 68
7.3.6 Other Analysis Sets 69
7.3.7 Subgroups 69
7.3.8 Protocol Deviations 69
7.4 Analysis Plan 70
7.4.1 Analysis of Demographic and Baseline Characteristics 70
7.4.2 Analysis of Primary Objectives 70
7.4.2.1 Statistical Hypotheses for Primary Objectives 70
7.4.2.2 Analysis Populations for Primary Objectives 70
7.4.2.3 Statistical Methods for Primary Objectives 70
7.4.2.4 Sample Size and Power Considerations of Primary Objectives 71
7.4.2.5 Analysis of Safety Objectives 71
7.4.2.5.1 Analysis of Extent of Exposure 71
7.4.2.5.2 Analysis of Solicited Local and Systemic Adverse Events and Other Reactions 71
7.4.2.5.3 Analysis of Spontaneously Reported Adverse Events 72
7.4.2.5.4 Analysis of Safety Laboratory Values 72

7.4.3 Analysis of Key Secondary Immunogenicity Objectives 72
7.4.4 Analysis of Key Secondary Efficacy Objectives 72
7.4.5 Analysis of Key Secondary Other Objectives 73
7.4.6 Analysis of Non-Key Objectives 73
7.5 Planned Interim Analysis 73
8.0 SOURCE DOCUMENTATION, STUDY MONITORING, AND AUDITING ..74
8.1 Source Documentation 74
8.2 Study Monitoring and Source Data Verification 75
9.0 DATA MANAGEMENT 76
9.1 Data Entry and Management 76
9.2 Data Clarification 77
9.3 Data Coding Procedures 77
9.4 Data Protection 77
10.0 RECORD RETENTION 78
11.0 USE OF INFORMATION AND PUBLICATION 79
12.0 ETHICS 80
12.1 Regulatory and Ethical Compliance 80
12.2 Informed Consent Procedures 80
12.3 Responsibilities of the Investigator and IRB/EC 80
12.4 Protocol Adherence 82
12.5 Protocol Amendments 82
13.0 REFERENCE LIST 83

LIST OF ABBREVIATIONS

AE	Adverse Event
AESI	Adverse Events of Special Interest
AP	(Statistical) Analysis Plan
ANC	Absolute Neutrophil Count
BCDM	Biostatistics and Clinical Data Management
BMI	Body mass index
CBC	Complete Blood Count
CHMP	Committee for Medicinal Products for Human Use
CI	Confidence Interval
CRF	Case Report Form
CRO	Contract Research Organization
DSMB	Data Safety Monitoring Board
EC	Ethics Committee
eCRF	Electronic Case Report Form
EDC	Electronic Data Capture
eDiary	Electronic Diary
EDT	Electronic Data Transfer
ELISA	Enzyme-linked Immunosorbent Assay
EMA	European Medicines Agency
FAS	Full Analysis Set
FDA	Food and Drug Administration
GCP	Good Clinical Practices
GMC	Geometric Mean Concentration
GMMA	Generalized Modules for Membrane Antigens
GMR	Geometric Mean Ratio
GMT	Geometric Mean Titer
HEE	Hidden Entry Envelopes
HLA	Human Leukocyte Antigen (complex)
HLA-B27	Protein B-27 of the HLA
IB	Investigator’s Brochure
ICF	Informed Consent Form
ICH	International Conference on Harmonisation of Technical Requirements for Registration of Pharmaceuticals for Human Use
ID	Intradermal
IM	Intramuscular
IN	Intranasal
IRB	Institutional Review Board
ITT	Intention-To-Treat
IUD	Intrauterine Device
MITT	Modified Intention-To-Treat
LSLV	Last Subject Last Visit
MedDRA	Medical Dictionary for Regulatory Activities
NOCD	New onsets of chronic diseases
NVGH	Novartis Vaccines Institute for Global Health
OMV	Outer Membrane Vesicles

PP	Per Protocol
PSB	Product Stewardship Board
SAE	Serious Adverse Event
SMT	Safety Management Team
ReA	Reactive arthritis
SC	Subcutaneous
SDA	Source Data Agreement
SOC	System Organ Class
SOP	Standard Operating Procedure
SPC	Summary of Product Characteristics
WHO	World Health Organization

1.0 BACKGROUND AND RATIONALE

Shigella spp. are Gram-negative bacteria that infect the intestinal epithelium and are major causes of diarrhea, including dysentery. *Shigella* is transmitted by the fecal-oral route and taken up by contaminated food or water. It is endemic throughout the world but the main burden of disease is in developing countries. In 2009, the World Health Organizations (WHO) estimated approximately 125 million cases of shigellosis per year in Asia alone [1]. Ninety-nine percent of all cases occur in developing countries and approximately 70% in children younger than 5 years of age [2]. Current estimates of mortality vary between 108,000 worldwide (http://www.who.int/vaccine_research/diseases/diarrhoeal/en/index6.html) and 14,000 in Asia where previously 80% of all deaths were estimated to occur. Sixteen serotypes (all 14 *S. flexneri*, *S. sonnei*, and *S. dysenteriae* type I) are considered to be of global importance [3] with *Shigella sonnei* being the most common serotype worldwide.
In target populations, treatment options for shigellosis are limited. Shigellosis can be treated with appropriate antibiotics. However, antibiotic resistance is increasing and many *Shigella* isolates are resistant to two or more of the common antibiotics ampicillin, chloramphenicol, nalidixic acid, co-trimoxasole. Resistance to third generation antibiotics, especially ciprofloxacin, has been reported to be emerging [3].Still effective antibiotics include ceftriaxone that is administered intramuscularly or intravenously and thus is not easily accessible for people in impoverished communities.

No vaccine is available. Natural infection (experimental infection or vaccination with attenuated *Shigella*), leads to good protective immune, but despite the generally high genetic conservation between serotypes, the protection is highly specific for the infecting serotype. This suggests that the dominant protective antigen is the O antigen (OAg) of the lipopolysaccharide (LPS). There have been many attempts to make a Shigella vaccine, using inactivated whole cell bacteria either orally or parentally (low efficacy, high reactogenicity for parenteral), attenuated live oral vaccines (no vaccine has yet obtained a useful balance between attenuation and efficacy), recombinant surface proteins (several projects at an early stage), O antigen conjugates [4]. The latter have been the most successful to date with a parenteral *S. sonnei and S. flexeneri type* 2 OAg conjugates tested in field trials in Israel that achieved 74% efficacy in adults [5] with one immunization and 71% efficacy in children 3 years of age and older with 2 vaccinations [6,7]. No significant efficacy was achieved in younger children in accordance with a very low immunogenicity in the young children. As expected for an OAg vaccine, this was highly serotype specific and the *S. sonnei* OAg afforded no protection against infection with *S. flexneri* nor did a *S. flexneri* 2a based OAg conjugate protect against *S. sonnei*. In addition, to achieve broad-spectrum protection against the 16 serotypes that are currently considered to be globally important, a multivalent OAg-vaccine will be needed. Therefore, new vaccine development approaches are needed.

Novartis Vaccines Institute for Global Health (NVGH) development strategy is based on a parenteral vaccine targeting the OAg but using a new platform technology called Generalized Modules for Membrane Antigens (GMMA) as a novel delivery system which may be applicable also for other vaccines against Gram-negative pathogens. GMMA are naturally shed from the surface of Gram-negative bacteria and consist of outer membrane

proteins, outer membrane lipids, including phospholipids and LPS, and enclosed periplasmic proteins [8]. In the previous literature, GMMA are called outer membrane vesicles (OMV). However, this term has also been used for vesicles derived by detergent-extraction of homogenized bacteria are used as vaccines, e.g. to control *Neisseria meningitidis* type B infections in New Zealand (MeNZB®). In order to differentiate the substantially different types of particles, the name 'GMMA' was introduced for the blebs released from the cell surface [9].

The candidate NVGH 1790GAHB vaccine is based on outer membrane particles that are naturally released from the *S. sonnei* during growth. The natural arrangement of the outer membrane is preserved during the release of GMMA and therefore GMMA allow an optimal exposure of the antigens of the outer membrane for recognition by the host immune system. NVGH has developed an economic process to purify GMMA in large quantities from high density cultures of bacteria genetically modified to increase GMMA production and generate a LPS with low endotoxicity, suitable for use in humans. *S. sonnei* was chosen as the proof of concept for the GMMA technology and as a prototype *Shigella* GMMA vaccine since *S. sonnei* is among the most common serotypes causing dysentery in humans.

The proposed trial is aimed to investigate the safety and immunogenicity of 1790GAHB vaccine when administered at different dosages by different routes in healthy adults. In the murine challenge model intranasal (IN) vaccination was more effective than subcutaneous (SC) vaccination suggesting that a mucosal vaccination route is beneficial. This is also in accordance with the current opinion that the most effective vaccination against enteric pathogens requires a mucosal vaccination route. Additionally, non-GLP studies in rabbits at NVGH support the use of OAg-GMMA by IN and intradermal (ID) vaccination routes, which are also suggested by the small size and high surface to volume ratio of GMMA. Finally, to have a IM evaluation in the same study where ID and IN are evaluated a group of subjects will be vaccinated by the IM route using a dose of 5 μg. Thus, the proposed trial will investigate 3 different vaccination routes in humans.

The study is also the proof of concept for the GMMA technology which may be used also for the development of other vaccines. The 1790GAHB vaccine proved to be highly immunogenic in mice where a SC dose as low as 1 μg was immunogenic. It is known from other vaccines (e.g. influenza vaccine) that the ID immunization triggers similar immune response as IM immunization at significantly lower dosages (with the Flu vaccine 20% of the IM dose when applied ID is as immunogenic as the full IM dose). Since this is the first time in man trial with a new vaccine, it was decided to start from 10% of the lowest IM dose (1 μg in the H03_01TP study) for the ID vaccination and therefore 0.1 μg has been selected as starting dose for ID administration. This dose, applied SC in mice, was still very immunogenic. For the IN administration in contrast, higher amounts of antigen than for IM are usually needed to trigger a good immune response. Therefore 5 μg has been selected as starting dose for IN administration. The highest dose proposed for IN administration is determined by the antigen concentration of the clinical lot (200 μg / mL) and by the maximum volume (200 μL) which can be efficiently administered in each nostril, resulting in a total dosage of 80 μg. While the assumption is that the lower dosages will be immunogenic, we also aim to test higher dosages that would allow the combination of multiple GMMA in a multivalent *Shigella*

vaccine. The 3-dose regimen tested in the study has been chosen to fully characterize the immunogenicity profile of the vaccine.

A comprehensive review of NVGH 1790GAHB vaccine is contained in the Investigator's Brochure (IB) supplied by NVGH; this document should be reviewed prior to initiating the study.

In the first part of this study and in another parallel study (H03_01TP) with the same vaccine there have been few subjects who have experienced a transient decrease of circulating neutrophils. This decrease was below the normal reference range, and in two cases (one in each study) it was classified as a severe adverse event, but not as a serious adverse event. Although this finding was not associated with any clinical illness, as recommended by the trial DSMB, it prompted the introduction of some urgent safety measures in this protocol to protect the safety of study subjects.

The trial will be conducted in compliance with the protocol, GCP and applicable regulatory requirement(s).

2.0 OBJECTIVES

2.1 Primary Objective(s)

Primary Safety Objective

To evaluate the safety profile of different dosages of NVGH 1790GAHB vaccine in healthy adults, when administered intradermally, intranasally or intramuscularly

2.2 Secondary Objectives

Secondary Immunogenicity Objectives

To evaluate the immunogenicity profile of different dosages of investigational 1790GAHB vaccine in healthy adults when administered intradermally, intranasally, or intramuscularly at 28 days after 1st vaccination, 28 days after 2nd vaccination and 28 and 168 days after 3rd vaccination by measuring the anti-LPS *S. sonnei* serum IgG.

Exploratory Objectives

To evaluate the mucosal immunity, induced by different dosages of investigational 1790GAHB vaccine in adults when administered intradermally, intranasally, or intramuscularly, in stool samples obtained at 28 days after 1st vaccination, 28 days after 2nd vaccination and 28 days and 168 days after 3rd vaccination, by measuring the anti-LPS fecal secretory IgA (sIgA).

To determine in as subset of subjects (each group of cohort C) the frequency of circulating memory B cells specific for the OAg of the vaccine and its carrier (GMMA) before vaccination, at 28 days after 1st vaccination, at 28 days after 2nd vaccination and at 28 and 168 days after 3rd vaccination.

To determine in a subset of subjects (each group of cohort C) the frequency of circulating vaccine specific antibody secreting cells B cells (plasma blasts) at day 7 after the 1st vaccination

3.0 STUDY DESIGN AND INVESTIGATIONAL PLAN

3.1 Overview of Study Design

This is a phase 1, randomized, placebo controlled single center study in adult women and men volunteers evaluating the safety and immunogenicity of three vaccinations with progressively of different dosages, administered via different vaccination routes, of the NVGH vaccine against *Shigella sonnei* infections (1790GAHB). As no vaccine is currently available against shigellosis, the safety profile of the 1790GAHB vaccine will be evaluated in comparison to that of a placebo, constituted by an aluminum hydroxide suspension with the same concentration as for study vaccine dosages. All subjects and blinded study personnel will be blinded throughout the study with respect to the assignment to treatment or placebo group, while information concerning dosage applied and route of administration will be available to all individuals involved.

This clinical trial has been designed to minimize pain, discomfort, fear and any other foreseeable risks. During the screening period, subjects giving informed consent will be screened for general health status. As part of the screening, subjects will be tested for the gene coding for the human leukocyte antigen (HLA) B27. Positive subjects, along with the one with history of reactive arthritis, will be excluded. This is done as if reactive arthritis is caused by an auto immune response; there is at least a possibility that it could be initiated by vaccination of susceptible people (i.e. HLA-B27 positive individuals) with 1790GAHB vaccine (see section 6.6.1.1). No pharmacokinetic tests will be performed as evaluation of pharmacokinetic properties is not required for vaccines unless new delivery systems are employed or when the vaccine contains novel adjuvants or excipients [10].

Subjects who meet all inclusion criteria and none of the exclusion criteria, with screening tests within acceptable values (as any minor abnormality considered not clinically significant) and women of child bearing potential with negative pregnancy test will be eligible for enrollment. Subjects being screened for enrollment into Cohort C after 10th July 2014 (date of amendment 4) with an absolute neutrophil count (ANC) less than $1.8x10^9$ at screening will not be eligible for enrollment. A total of 52 eligible subjects will be randomly assigned, to receive three vaccinations, 4 weeks apart, with either 1790GAHB vaccine (at the lower dose for the IN and ID administration) or placebo. For the IM vaccinations, subjects will be randomly assigned to receive either 1790GAHB vaccine or placebo and will not be included into the dose escalation.

Therefore with a dose escalating approach, three antigen concentrations will be tested for both the IN and the ID vaccination routes. For the IM vaccination route, a single antigen concentration of 5 μg will be tested.

Route	Investigational Vaccine	No. of Subjects receiving treatment	No. of Subjects receiving placebo
**ID**	1790GAHB (0.1 μg*)	4	2
	1790GAHB (1 μg*)	6	2
	1790GAHB (10 μg*)	6	2
**IN**	1790GAHB (5 μg*)	4	2
	1790GAHB (20 μg*)	6	2
	1790GAHB (80 μg*)	6	2

**IM**	1790GAHB (5 µg*)	6	2
	Total	52	

*protein content

Specifically for IN and ID administration routes, a DSMB (Data Safety Monitoring Board) will be in place to receive a summary of all safety data obtained during one week follow-up post-first vaccination with the lower dose. Based on evaluation of the safety data, the DSMB will make a recommendation, as to whether the next cohort should be vaccinated with higher antigen concentration or not.

Groups will be recruited in 3 cohorts in the following sequence:

Cohort A = 0.1 µg ID, 5 µg IN
Cohort B = 1 µg ID and 20 µg IN
Cohort C = 10 µg ID and 80 µg IN and 5 µg IM

Within each cohort, subjects will be randomized to receive three vaccinations, four weeks apart, of either 1790GAHB vaccine or placebo administered intranasally or intradermally (cohort A), intranasally or intradermally (cohort B) intramuscularly, intranasally or intradermally (cohort C).

For the first cohort (cohort A), there will be exactly 12 subjects enrolled. The following rules will be applied for administering the investigational vaccine to the next volunteer: randomization will be such that for the first two subjects to be randomized in cohort A, one will receive placebo and the other 1790GAHB vaccine given by the intradermal, or intranasal route, without affecting the blinding. If there is no occurrence of SAE related/suspected to vaccination at 48 hours after the 1st vaccination of the first 2 subjects, the remaining subjects (10 in total) may be vaccinated, with no more than 6 vaccinations in a day.

For the cohort B there will be exactly 16 subjects enrolled. The same rules, as for cohort A, will be applied for administering the investigational vaccine to the first two subjects in cohort B (first two subjects randomized to receive placebo or 1790GAHB vaccine either intradermally or intranasally, with subsequent 14 subjects vaccinated following satisfactory safety review at 48 hours).

For the cohort C there will be exactly 24 subjects enrolled. The same rules, as for cohort A, will be applied for administering the investigational vaccine to the first two subjects in cohort C (first two subjects randomized to receive placebo or 1790GAHB vaccine either intramuscularly, intradermally or intranasally, with subsequent 22 subjects vaccinated following satisfactory safety review at 48 hours).

Independent dose escalation recommendations will be made for IN and ID vaccination routes. At the end of the 7 days observation period following 1st vaccination, a summary of all safety data (solicited local and systemic reactions, unsolicited adverse events and SAE) and listings of clinically significant modifications in hematology, blood chemistry and urinalysis test values obtained will be provided to the DSMB. Based on evaluation of the safety data, the DSMB will make a recommendation, as to whether the next cohort should be vaccinated with higher antigen concentration or not. Enrollment and

vaccination for the subsequent cohort (cohort B) will be started immediately after DSMB confirmation.

Subjects will be observed at the clinic for approximately 2 hours after each vaccination. A Diary card will be used to collect solicited, unsolicited adverse events, and medication/vaccinations given during 7 days (inclusive) following 1st vaccination. Seven days following 1st vaccination, a clinical visit will be performed to verify and collect all safety data occurred during the one week follow-up post-first vaccination. After day 7 following 1st vaccination, only unsolicited adverse events, solicited reactions that continue beyond day 7, and related medications will be collected in the diary card until the time of return to the clinic for the 2nd vaccination. Following 2nd and 3rd vaccination one Diary Card will be used to collect solicited, unsolicited adverse events, and medication/vaccinations given during 7 days (inclusive) following vaccination and unsolicited adverse events, solicited reactions that continue beyond day 7, and related medications until the time of return to the clinic for visit 4 and 5 following administration of the 2nd and 3rd vaccinations respectively. Blood for a complete blood count (CBC) will also be obtained 7 days after the 2nd and 3rd vaccinations (administered at visits 3 and 4 respectively), as well as 168 days after the 3rd vaccination, to assess the ANC. All individuals with a neutropenia (ANC $<1.8x10^9$/L) will have the test repeated on a weekly basis until the neutropenia resolves (ANC $\geq 1.8x10^9$/L). If the neutrophil count does not rise to $1.8x10^9$/L by day 21 after vaccine administration, the subject will be discontinued from further vaccination. In case the ANC is less than $0.5x10^9$/L after vaccination (AE Grade 4), the subject will have the test repeated on a weekly basis until the neutropenia resolves and will be discontinued from further vaccination if this takes place after the 1st or 2nd vaccination.

All subjects will be followed up for 6 months after 3rd vaccination. All serious adverse events (SAEs), all medications given to treat SAEs, all new onset of chronic disease, all AEs leading to vaccine/study withdrawal, and reactive arthritis (adverse event of special interest (AESI)) will be collected for the entire study.

3.1.1 Study Period

Expected duration of the study for an individual subject is 9 months. Each subject will be followed-up for 6 months after the 3rd vaccination.

3.2 Study Visit Procedures

The sections that follow provide an overview of the procedures that are to be followed in enrolling, evaluating, and following subjects who participate in this clinical trial.

Table 3.2-1: Study Visit Procedures

Type of Visit	Procedures
Screening Visit	Informed consent (refer to sections 3.2.1 and 12.2), demography, prior/concomitant medications, medical history, review of systems, physical examination**(including vital signs):**, review with any women of childbearing potential their commitment to practice birth control, pregnancy testing for any women of childbearing potential, vital signs, Urinalysis height/weight measurement, safety laboratory screening assessments (refer to Table 2), blood draw for HLA-B27 evaluation, agglutination test of *S. sonnei*, review of eligibility criteria (refer to sections 4.1 and 4.2 for the list).
Visit 1 – Vaccination Visit	Confirmation of review of eligibility criteria (refer to sections 4.1 and 4.2 for the list), review of systems, physical examination **(including vital signs):**pregnancy testing for any women of childbearing potential, enrollment (refer to section 3.2.3), randomization (refer to section 3.2.4), blood sampling, stool sampling, vaccination, training on measurements and diary card, observation of subject, scheduling of future clinic visits, discharge instructions
Visit 2 - Visit After Vaccination	Diary card data review and collection, laboratory screening assessments (refer to Table 2), training on measurements and diary card, scheduling of future clinic visits, discharge instructions<br>If the absolute neutrophil count (ANC) is found to be below $1.8x10^9$/L, then the test will be repeated on a weekly basis until resolution (ANC $\geq 1.8x10^9$/L). The subject will be discontinued from further vaccination if the count is still less than $1.8x10^9$/L on day 21 following vaccination.<br>If the ANC is less than $0.5x10^9$/L after vaccination, the test for ANC will be repeated on a weekly basis until resolution (ANC $\geq 1.8x10^9$/L) and the subject will be discontinued from further vaccination
Visits 3 – Visit After Vaccination and Vaccination Visit	Review of systems, physical examination **(including vital signs):**), diary card data review and collection, confirmation of eligibility criteria (refer to sections 4.1 and 4.2 for the list), pregnancy testing for any women of childbearing potential, blood sampling, stool sampling, vaccination, training on measurements and diary card, observation of subject, scheduling of future clinic visits, discharge instructions
Visits 4 – Visit After Vaccination and Vaccination Visit	Review of systems, physical examination **(including vital signs):** Diary card data review and collection, confirmation of eligibility criteria (refer to sections 4.1 and 4.2 for the list), pregnancy testing for any women of childbearing potential, laboratory screening assessments (refer to Table 2),blood sampling, stool sampling, vaccination, training on measurements and diary card, observation of subject, scheduling of future clinic visits, discharge instructions
Visit 5 - Visit After Vaccination	Diary card data review and collection, laboratory screening assessments (refer to Table 2), blood sampling, stool sampling, scheduling of future clinic visits, discharge instructions
Visit 6 - Termination Visit	See text below and refer to section 3.8 for early termination visit procedures
Visit for lab assessment of absolute neutrophil count (ANC)<br>(visi 3.1 and 4.1)	At day 7 following administration of the 2nd and 3rd vaccinations (at visit 3 and 4 respectively), as well as 168 days following administration of the 3rd vaccination (at visit 6), a blood sample will be collected to test for a complete blood count. If the ANC is found to be below $1.8x10^9$/L, then the test will be repeated on a weekly basis until resolution (ANC $\geq 1.8x10^9$/L). The subject will be discontinued from further vaccination if the count is still less than $1.8x10^9$/L on day 21 following vaccination.<br>If the ANC is less than $0.5x10^9$/L after vaccination, the test for ANC will be repeated on a weekly basis until resolution (ANC $\geq 1.8x10^9$/L) and the subject will be discontinued from further vaccination

3.2.1 Informed Consent/Assent

"Informed consent" is the voluntary agreement of an individual or his/her legal guardian to participate in research. Consent must be given with free will of choice, and without undue inducement. The individual must have sufficient knowledge and understanding of the nature of the proposed research, the anticipated risks and potential benefits, and the requirements of the research to be able to make an informed decision.

Informed consent following local IRB/EC guidance **must** be obtained before conducting any study-specific procedure (i.e., all of the procedures described in the protocol). The process of obtaining informed consent should be documented in the subject source documents in addition to maintaining a copy of the signed and dated informed consent.

If a subject is unable to read, an impartial witness should be present during the entire informed consent discussion. An impartial witness is defined as a person who is independent from trial conduct, who cannot be unfairly influenced by those involved with the trial, who attends the informed consent process if the subject or the subject's legally acceptable representative cannot read, and who reads the informed consent form and any other written information supplied to the subject. After the written informed consent form and any other written information to be provided to subjects, is read and explained to the subject has verbally consented to the subject's participation in the trial and, if capable of doing so, has signed and personally dated the informed consent form, the witness should sign and personally date the consent form. By signing the consent form, the witness attests that the information in the consent form and any other written information was accurately explained to, and apparently understood by, the subject and that informed consent was freely given by the subject.

3.2.2 Screening Procedures

During the screening period before Visit 1 (day -28), after an individual has consented to participate in the study and informed consent is signed (see sections 3.2.1 and 12.2), that individual will be given a unique 5-digit screening number (assigned sequentially by the site from 80001), which is documented in the Screening Log.

Subjects will be screened to determine eligibility based on the inclusion and exclusion criteria as provided in section 4:

i. Collect demography, review of medical history (see section 6.2), prior and concomitant medication and blood products received (refer to section 5.4 for further details)

ii. Complete physical examination to verify subject's vital signs and the general health status, including: check of general appearance, height/weight, review of respiratory system (respiratory rate) and cardiovascular system (systolic/diastolic blood pressure, heart rate pulse rate), oral temperature as described in section 6.2. A structured interview that queries the subject as to any complaints the subject has experienced across each organ system should be used.

iii. Obtain approximately 14 mL blood for screening laboratory assessments: hematological and hematochemical tests, including virological tests for hepatitis B and C and HIV antibody screens (refer to list available in synopsis Table 2) Collect urine sample for urinalysis dipstick. In case the deviations of dipstick results are considered clinically significant, obtain a urine sample for the microscopic tests (refer to list available in synopsis Table 2)

iv. Perform the specific test to evaluate agglutination of *S. sonnei* as described in section 3.5.4 on serum from approximately 2ml blood

v. Review with any women of childbearing potential their commitment to practice birth control. Women of childbearing potential are defined as a post onset of menarche and pre-menopausal female capable of becoming pregnant. This does not include females who meet any of the following conditions: (1) menopause at least 2 years earlier, (2) tubal ligation at least 1 year earlier, (3) total hysterectomy or (4) post bilateral oophorectomy. Suitable methods of birth control include hormonal contraceptive (such as oral, injection, transdermal patch, implant, cervical ring); barrier methods (condom or diaphragm with spermicide); intrauterine device (IUD).

vi. Obtain approximately 4 ml blood for the HLA-B27 test (see section 6.6.1.1)

Approximately 4 mL blood draw for the evaluation of human chorionic gonadotropin (hCG) will be performed at screening and a urine dipstick will be performed at Visit 1 before enrollment (refer to section 3.5.2 for guidance regarding the procedure) to exclude pregnancy to all women of childbearing potential.

In the event that the individual is determined ineligible for study participation, he/she is considered a “screen failure”. The reason for screen failure must be documented in the Screening Log. If the individual is determined to be eligible for the study, he/she should be assigned an enrollment number and enrolled into the study at Visit 1, as described in section 3.2.3.

3.2.3 Enrollment

At Visit 1, once the following are performed:

1) Verification of results of screening laboratory assessments, medical history since screening visit (to ensure the subject is healthy and determined to be eligible for study participation).

2) Complete physical examination to verify subject’s vital signs and the general health status, including: check of general appearance, review of respiratory system (respiratory rate) and cardiovascular system (systolic/diastolic blood pressure, heart rate, pulse rate), oral temperature as described in section 6.2.

3) Review of eligibility criteria (refer to sections 4.1 and 4.2 for the list)

4) A Urine pregnancy test for the evaluation of human chorionic gonadotropin (hCG) to exclude pregnancy (refer to section 3.5.2 for guidance regarding the procedure) to all women of childbearing potential.

the subject will be enrolled using the screening number assigned by the investigator and then randomized using Hidden Entry Envelopes (HEEs) containing vaccines group information.

At randomization, the subject will be assigned a subject code and ID. The subject code consists of the 3 letters subject's initials (the 1st letter of the surname name followed by the 1st letter of the 1st name and the 1st letter of 2nd name). In case the subject has only 2 initials a dash or a "X" will be recorded. The Subject ID consists of a 5-digit number resulting from the combination of the site number (10), the stratification identifier corresponding to the cohort (with cohort A= 1 up to cohort B=3), and the subject's order of randomization within each cohort (starting from 01). Once assigned to a subject, the subject ID cannot be reused.

3.2.4 Randomization

Enrolled subjects will be randomly assigned to one of 3 sequential cohorts (the one actively enrolling). Within each cohort (A, B and C), subjects will be randomized in a pre-specified ratio of 2:1 (0.1 µg ID and 5 µg IN) and 3:1 (all other dosages) to receive either 1790GAHB vaccine or placebo administered intranasally or intradermally (cohort A), intranasally or intradermally (cohort B), intramuscularly, intranasally or intradermally (cohort C).

Designated unblinded personnel at the NVx Biostatistics and Clinical Data Management (BCDM) department will be responsible to the production of the HEEs using a validated system. HEE will allow revealing the vaccination group assignment to dosage and treatment or placebo groups according to the subject numbers on a per-subject basis. The HEEs will be delivered to the study site prior to the initiation of the study; they must be stored in a secure place and opened one by one, only after the subject has been found eligible to be randomized.

Screening phase will be performed until a total of 52 subjects will be enrolled into the study. If for any reason, after signing the informed consent form (ICF), the subject (who has passed screening) fails to be randomized, the reason for not being randomized should be recorded in source documents.

Additional subjects may be randomized into the study at the discretion of the sponsor in the case of any subject who is randomized but does not receive any study vaccine. Subjects withdrawn after 1st vaccination or lost to follow up will not be replaced.

3.2.5 Visit Procedures

3.2.5.1 Pre-vaccination Procedures

Review safety information from previous visit to ensure subject is healthy and meet eligibility. Perform a complete physical examination to verify subject's vital signs and the

general health status, including: check of general appearance, review of respiratory system (respiratory rate) and cardiovascular system (systolic/diastolic blood pressure, heart rate, pulse rate), temperature as described in section 6.2. Prior to each study vaccination (Visit 1, Visit 3 and Visit 4), approximately 20 mL blood will be drawn and stools samples collected from the subject for the *Shigella sonnei* serology testing and pregnancy testing performed for all women of childbearing potential. In cohort C only, an additional blood sample will be collected for cellular mediated immunity testing.

3.2.5.2 Vaccination Procedures

Three vaccinations will be administered with a 4 weeks interval on study day 1, 28 and 56. However, as study days should be calculated based on the actual date of the previous visit, the actual day of vaccination may slightly change.

After confirming eligibility and enrolling subject into the study on Day 1, perform vaccination of the subject according to the assigned study vaccine and route and according to the procedures described in section 5.3 and observing the blinding procedures described in section 3.3. At later clinic visits that involve vaccination (Visit 3 and 4), confirm that the subject does not meet any criteria for delaying or cancelling additional study vaccinations, as described in section 4.3 and section 4.4 of the protocol.

3.2.5.3 Post-vaccination Procedures

After each vaccination, the subject will be observed for approximately 2 hours including observation for unsolicited adverse events, solicited adverse events, and body temperature measurement. Take the opportunity to remind the subject how to measure solicited reactions and body temperature as part of this observation period. Record all safety data collected in the subject’s source documents. At approximately 30 and 120 minutes of the observation period, perform a review of respiratory system (respiratory rate) and cardiovascular system (systolic/diastolic blood pressure, heart rate pulse rate).

Subject should be carefully trained on how to measure local reactions and body temperature, how and how often to complete the diary card. Training should be directed at the individual(s) who will perform the measurements of reactions and those who will enter the information into the diary card. This individual may not be the subject, but if a person other than the subject enters information into the diary card, this person’s identity must be documented in the study file and this person must receive training on the diary card. Training of the subject on how to measure an injection site reaction should be performed while the subject is under observation after vaccination. The subject must understand that timely completion of the diary card on a daily basis is a critical component to study participation. The subject should also be instructed to write clearly and to complete the diary card in pen. Any corrections to the diary card that are performed by the person completing the diary card should include a single strikethrough line with a brief explanation for any change. No changes can be made to the diary card when it is returned to the clinic.

Starting on the day of vaccination, the subject will check in the evening for specific types of reactions at the vaccination site (solicited local adverse events), any specific generalized symptoms (solicited systemic adverse events), body temperature (taken

preferably orally) any other symptoms or change in the subject's health status, and any medications taken (excluding vitamins and minerals).

Body temperature measurement is to be performed using the thermometer provided by the site. If the subject feels unusually hot or cold during the day, the subject should check body temperature. If the subject has fever, the highest body temperature observed that day should be recorded on the Diary Card. The measurement of diameter of solicited local adverse events is to be performed using the ruler provided by the site. The collection of body temperature, solicited local adverse events, solicited systemic adverse events will continue for a total of 7 days on the Diary Card. The collection of unsolicited adverse events and medications will continue for 28 days on the Diary Card.

At the end of the observation period, the site should schedule the next study visit with the subject. The subject will receive a volunteer study card to be used as a written reminder of the next planned study activity and to provide study staff contact details. The subject will be reminded to complete the Diary card daily and to contact the site if there are any questions and to contact the site immediately (or as soon as the subject is medically stable) if the subject has a medical condition that leads to a hospitalization or an emergency room visit.

All subjects presenting with a history of fever or any other sign or symptom judged by the investigator to be as a result of an infection within 21 days of vaccination will have a blood sample collected for a complete blood count to establish the absolute neutrophil count. If there is a neutropenia, the subject will be managed appropriately according to local and international requirements.The subject will be withdrawn from further vaccination if the ANC is still $<1.8x10^9$/L at day 21 post vaccination. If the case meets the definition of a febrile neutropenia, with an ANC $<1.0x10^9$/L then this would meet the criteria a study hold (see section 3.6).

3.2.5.4 Clinic Visits After 1st Vaccination

Clinic visits that do NOT include vaccine administration will be performed on Visit 2 (7 days after Visit 1), Visit 5 (28 days after Visit 4) and Visit 6 (168 days after Visit 4).

Clinic visits that DO include vaccine administration will be performed on Visit 3 and Visit 4.

At the clinic visits 2, 4 and 5, approximately 18 mL blood for screening laboratory assessments (hematological and hematochemical tests, refer to list available in synopsis Table 2) will be obtained and the diary card will be reviewed. Please see section 3.4.1 for additional guidance on diary card review. The subject will be interviewed to determine if any unsolicited adverse events occurred and if any concomitant medications or vaccines were taken/received in the time since the last clinic visit. The healthcare professional reviewing these data will discuss the symptoms (if any) reported by the subject and will determine if any additional diagnoses and/or adverse events are present.

Clinic visits occurring on Visit 3 (28 days after Visit 1), Visit 4 (28 days after Visit 3), Visit 5 (28 days after Visit 4) and Visit 6 (168 days after Visit 4) will also include approximately 20 mL blood draw for the *Shigella sonnei* serology testing. Clinic visit occurring on Visit 4, Visit 5 and Visit 6 will also include a stool sampling for the *Shigella*

sonnei serology testing. In cohort C only, an additional blood sample will be collected for cellular mediated immunity testing.

Blood samples for a complete blood count will be obtained 7 days after the 2nd and 3rd vaccinations (administered at visits 3 and 4 respectively), as well as 168 days after the 3rd vaccination (at Visit 6) to assess the ANC.

The site should schedule the next study activities with the subject. The subject will receive a written reminder of the next planned study activity and will be reminded to contact the site if there are any questions and to contact the site immediately (or as soon as the subject is medically stable) if the subject has a medical condition that leads to a hospitalization or an emergency room visit.

3.2.5.5 Reminder Telephone Calls

Reminder calls will be performed 2 and 6 days following each vaccination. The purpose of this contact with the subject is only to remind the subject about completion of the diary card and it is not intended to be for collection of safety data. If the subject wishes to describe safety information, this information should only be collected by a trained healthcare professional at the site, and the safety data described must be written down in source documents. The subject should be reminded to write the information down in the diary card and to contact the site via the telephone number provided in the informed consent to discuss medical questions.

3.2.5.6 Safety Calls

Not applicable.

3.2.5.7 "For cause" Visits

Not applicable.

3.2.5.8 Termination Visits

The termination visit will occur 6 months (calculated as 168 days) following 3rd vaccination. A blood sample for complete blood count will be obtained. For visit procedures to be performed for a subject whose planned study participation ends prematurely, please see section 3.8.

At the clinic visit, the following procedures will be performed: interview of subject to collect safety information (SAEs) not already notified and related concomitant medications, blood and stools sampling for *S. sonnei* serology testing. After thanking the subject for the study participation, the site will review the plan of when information relating to the subject's participation in the study may be available (e.g., study results, treatment assignments). It will also be discussed how information relating to the subject's participation in the study will be shared with the subject's healthcare provider, if the subject chooses to share this information.

The site will complete the termination CRF page and this will mark the completion of the subject's participation in the study.

3.3 Blinding Procedures

The identity of the study vaccine and placebo cannot be concealed as presentation and dilutions steps to be performed are different for vaccine and placebo.

The study will be observer-blind with respect to the assignment to treatment or placebo group while information concerning dosage applied to each group and route of administration will be available due to the study design. During the study, designated unblinded trained and qualified site staff (please see section 5.3) will be responsible for preparing and diluting the study vaccines or placebo out of view of the subject and an unblinded nurse(s) or physician(s) will be responsible for administering the study vaccines to the subjects. The unblinded staff will be instructed not to reveal the identity of the study vaccines either to the subject or the other investigative site personnel involved in the monitoring of conduct of the trial. The designated unblinded staff , nurse(s) or physician(s) will not take part in evaluating the subject(s) for safety or collect study data after the administration of the study vaccine.

Study vaccines allocations will not be freely available to the investigator or personnel monitoring the trial until after the completion of the trial and final data review. Adherence to the randomization list will be verified by a designated and unblinded Study Monitor, independent of the staff involved in the regular monitoring of the study, by checking the randomization list against the vaccination records maintained at the study site.

For each cohort, after all subjects have completed enrolment and all vaccinations and post vaccination results (one month after dose 1, 2 and 3) are available, a group- unblinded preliminary immunogenicity analysis and a blinded interim safety analysis may be performed. Individual subject results from preliminary / interim analyses will not be made available to site and sponsor personnel until the end of the study. Further details regarding the interim analysis are contained in section 7.5 of the protocol.

<u>Emergency unblinding</u>

This should be undertaken ONLY when it is essential for effective treatment of the subject. There are limited circumstances in a vaccine study where unblinding would change the course of medical treatment or care of a subject. If possible, sponsor should be contacted to discuss unblinding. In case of an emergency unblinding, the investigator will access the appropriate set of HEE to retrieve the vaccine code for the subject at risk. Date, time, and reason for unblinding must be noted. The unblinded vaccine code should not be recorded on the CRF. The investigator must also immediately inform NVGH local monitor that the code has been broken.

3.4 Data Collection

3.4.1 Data Collected From Subjects

All data collected from subjects and provided to the sponsor for analysis must be stripped of any identifiers that reveal the identity of that individual (beyond the use of subject ID, as described in section 3.2.3).

Diary cards will be the only source document allowed for solicited systemic and local adverse events (including body temperature measurements). During the study, 4 Diary Cards will be used to collect solicited local and systemic reactions (including body temperature measurements) and adverse events as follows:

1. From Visit 1 to Visit 2
2. From Visit 2 to Visit 3
3. From Visit 3 to Visit 4
4. From Visit 4 to Visit 5

The following additional rules apply to documentation of safety information collected by diary card:

1. No corrections or additions to the diary card will be allowed after it is delivered to the site.
2. Any blank or illegible fields on the diary card must be described as missing in the CRF.
3. The site must enter all readable entries in the diary card into the CRF, including those values that may be biologically implausible (e.g. body temperature: 400°C).
4. Any illegible or implausible data should be reviewed with the subject. If an underlying solicited or unsolicited adverse event is described. For example, if the subject with a body temperature of 400°C describes that the body temperature was actually 40°C on the day in which body temperature: 400°C was written into the diary card, this fever of 40°C should be described in the study file and reported as an unsolicited adverse event **in the adverse event CRF**.
5. Any newly described safety information (including a solicited reaction) must **NOT** be written into the diary card and must be described in the study file as a verbally reported event. Any adverse reaction reported in this fashion must be described as an unsolicited reaction and therefore entered on the adverse event CRF.

3.4.2 Electronic Case Report Forms

An electronic data capture (EDC) system (e.g., Inform™) will be used to expedite the entry of data. The investigator will enter data in English into the web enable EDC system in a timely manner; the data will be stored in Novartis Vaccines and Diagnostics' (NVx) clinical database management system. eCRF data will be reviewed routinely by NVx BCDM Group and NVGH clinical monitors or representatives.

The information from the diary will be entered in the eCRF. All data not recorded directly on the eCRFs must be verified by checking eCRF entries against source documents in order to ensure that the data have been completely and accurately reported as required by the study protocol.

Source data verification will be performed and recorded following NVGH internal SOP. The subject must also allow access to his/her medical records. Each subject will be informed of this prior to the start of the study.

3.5 Laboratory Assessments

3.5.1 Processing, Labeling and Storage of Serum Samples for Serology

A maximum of approximately 20 mL sample of blood will be drawn from all subjects at Visits 1, 3, 4, 5 and 6. The blood volume will not exceed 20 mL at each time point in order to provide the necessary serum volume (approximately half of the blood draw volume) for the serology assays. Blood samples must be collected in the appropriate manner, using exclusively materials and guidelines supplied by the sponsor. The investigator must ensure that his/her personnel and the laboratory(ies) under his/her supervision comply with this requirement.

Serum samples will be stored frozen below -20ºC. Shipment to the laboratories for analysis will be performed according to sites guidelines provided by the sponsor.

Complete instructions for processing, labeling, storage and shipping of samples are included in the Laboratory Manual provided by sponsor and available in the Investigator Site File.

Samples will be retained in accordance with regulatory guidance for retention of essential study documents as described in section 10, provided that the integrity of the sample permits.

3.5.2 Processing, Labeling and Storage of Stools Samples for Serology

A stools sample will be obtained from all subjects at Visits 1, 3, 4, 5 and 6. Stools samples must be collected in the appropriate manner, using exclusively materials and guidelines supplied by the sponsor. The investigator must ensure that his/her personnel and the laboratory(ies) under his/her supervision comply with this requirement.

Subject will be instructed to obtain stool samples at home starting from the day before until the morning of the site visit. Stools samples will be kept at -20°C (or refrigerated at 4°C if not possible to freeze) before being frozen at -80ºC at the study site.
Shipment to the laboratories for analysis will be performed according to sites guidelines provided by the sponsor.

Complete instructions for processing, labeling, storage and shipping of stools samples are included in the Serology Manual provided by sponsor and available in the Investigator Site File.

Samples will be retained in accordance with regulatory guidance for retention of essential study documents as described in section 10, provided that the integrity of the sample permits

3.5.3 Pregnancy Testing

For all women of child-bearing potential (see definition in section 3.2.2), the following pregnancy tests to exclude pregnancy will be performed:

- A blood draw for the evaluation of human chorionic gonadotropin (hCG) at screening and at study conclusion (Visit 6).

- A urine pregnancy test for the evaluation of human chorionic gonadotropin (hCG) at Visit 1 before randomization and before each vaccination (Visit 3 and 4).

3.5.4 Safety Laboratory Assessments

During the screening period (day -28), a maximum of approximately 25 mL sample of blood will be drawn for pregnancy testing (approximately 4 mL) hematological and hematochemical tests (approximately 10 mL) , virology (approximately 4 mL) and HLA testing (approximately 4 mL) (refer to Table 2). Urine dipstick (and urinalysis as applicable) tests will also be performed.

Subjects being screened for enrollment into Cohort C after 10th July 2014 (date of amendment 4) with a neutrophil count below $1.8x10^9$ at screening will be excluded.

A specific agglutination test of *S. sonnei* will be performed as part of the screening to exclude subjects with high antibody titers at baseline. In case the serum agglutinates a standard amount of inactivated bacteria, the subject will be excluded. Complete instructions for agglutination test and material to be used will be provided by sponsor and available at the study site.

One additional blood draw of approximately 10 mL for hematological and hematochemical tests will be obtained and urine dipstick urinalysis will be repeated at 7 days after 1st vaccination (Visit 2), 28 days after 2nd vaccination (Visit 4) and 28 days after 3rd vaccination (Visit 5). Clinically significant modifications in hematology, blood chemistry and urinalysis test values will be assessed by medical judgment based on interpretation of deviations from institution's normal values (see Table 2) and recommendations from CBER FDA GUIDANCE FOR INDUSTRY: Toxicity Grading Scale for Healthy Adult and Adolescent Volunteers Enrolled in Preventive Vaccine Clinical Trials.

Seven days after the 2nd and 3rd vaccinations (administered at visits 3 and 4 respectively),and 168 days after dose 3 (at Visit 6) samples of up to 3mL for complete blood counts will be collected. All individuals with a neutropenia (ANC $<1.8x10^9$/L) will have the test repeated on a weekly basis until the neutropenia resolves (ANC$\geq1.8x10^9$/L). If the neutrophil count does not rise to $1.8x10^9$/L by day 21 after vaccine administration, the subject will be discontinued from further vaccination. In case the ANC is less than $0.5x10^9$/L after vaccination (AE Grade 4), the subject will have the test repeated on a weekly basis until the neutropenia resolves and will be discontinued from further vaccination if this takes place after the 1st or 2nd vaccination.

Safety laboratory assessments will be performed at the site laboratory, and results of these tests will be recorded in the source documents and in the e-CRFs.

3.5.5 Cell Mediated Immunity Assessments

Cell Mediated Immunity will be tested in a subset of study subjects (see exploratory objectives). More specifically, a sample of approximately 32 mL of blood will be drawn from all subjects of Cohort C (N=24) at Visits 1, 2, 3, 4, 5 and 6.

The instructions for handling the sample to be used to test the cellular mediated immunity are included in the site Peripheral Blood Mononuclear Cells processing procedure.

Shipment to the laboratories for analysis will be performed according to sites guidelines provided by the sponsor.

3.5.6 Culture/PCR/Genotyping Assessments

Not Applicable.

3.6 Stopping/Pausing Guidelines

This is a dose escalation study. The decision to proceed with the clinical testing of the progressively higher ID or IN dosage will be made by the sponsor following advice by the DSMB (Data Safety Monitoring Board) as described in section 6.9.

In case related/suspected SAE will occur, enrollment of new subjects and further vaccinations for all routes will be on-hold while waiting for DSMB recommendation is received by the sponsor (see section 6.9).

The occurrence of two cases of Grade 3 or Grade 4 neutropenia, or of febrile neutropenia, in one study cohort will result in study hold, unblinding of data, discussion of results with safety management team (SMT) and the product stewardship board (PSB), and final decision made in consultation with DSMB and authorities. Grade 3 neutropenia refers to an ANC of $0.5\text{-}1.0x10^9$ cells/L, while grade 4 is defined as an ANC $<0.5x10^9$/L. Febrile neutropenia is defined as ANC $<1.0x10^9$/L associated with fever.

Independent of the DSMB, NVGH, as a sponsor, retains the right to halt the study at any time if there is a safety concern. If the study is prematurely terminated, the sponsor will promptly inform Regulatory Authorities and Ethic Committees on the decision of stopping the trial and no further enrollment or study vaccinations will occur until written authorization is provided by the sponsor in conjunction with a recommendation to proceed by the DSMB and in consultation with Regulatory Authorities and Ethic Committees, as appropriate.

3.7 Premature Withdrawal and Early Study Termination

A subject may discontinue study participation at any time prior to the last planned study visit. This is referred to as **premature withdrawal from the study** (see below for a description of withdrawal from study vaccine for subjects which refers to those subjects who do not receive additional vaccine doses but continue in the study for safety follow-up and/or other procedures). The reasons for premature withdrawal from the study include:

- Adverse event

- Death
- Withdrawal of consent
- Lost to follow-up
- Administrative reason
- Protocol deviation
- Other

NOTE: Before entering any alternate category as the reason for the subject's discontinuation from the study, the investigator should make every effort to investigate whether or not safety concerns (adverse event or death) may have been related to the subject's discontinuation from the study. If a safety concern has been associated with the subject's discontinuation, this must be described on the Termination CRF page, even if it is not the primary reason for the subject's discontinuation.

For any subject withdrawing from study participation prior to the planned Termination visit, it is important to determine if an AE was associated with the reason for discontinuing the study. This AE must be identified on the AE CRF page by indicating "Withdrawn from study due to AE".

For any subject withdrawn from study participation due to death, this should be noted on the Termination CRF page and the associated SAE that led to the death must be reported.

The subject can withdraw consent for participation in the study at any time without penalty or loss of benefit to which the subject is otherwise entitled. Reason for early termination should be deemed as "withdrawal of consent" if the subject withdraws from participation due to a non-medical reason (i.e., reason other than AE). If the subject intends to withdraw consent from the study, the investigator should clarify if the subject will withdraw completely from the study or if the subject will continue study participation for safety or a subset of other study procedures. If complete withdrawal from the study by the subject is specified, no further study interventions will be performed with the subject.

The date of termination is the date of the last contact (clinic visit or telephone) in which the subject's health status was assessed or, in cases where the subject does not agree to any further safety follow-up; it is the date consent is withdrawn.

For subjects who fail to show up for scheduled visits (clinic or telephone contacts), study staff are encouraged to make at least three documented attempts to contact the subject by telephone and at least one documented written attempt to contact the subject and encourage the completion of study termination procedures. These efforts to contact the subject should be recorded in the source documents. The termination date for the subject to be captured on the Termination CRF page is the date of the last successful visit (clinic or telephone) with the subject.

For subjects who are withdrawn from the study due to sponsor decision (e.g., meeting pre specified withdrawal criteria or termination of study by the sponsor), this reason should be noted in the Termination CRF page and any ongoing AEs at the time of study withdrawal must be followed until resolution/stabilization.

In general, subjects associated with protocol deviations may remain in the study unless continuation in the study jeopardizes the subject's health, safety, or rights. For subjects who are withdrawn from the study due to receipt of an excluded medication/vaccination or due to significant protocol non-compliance, this reason should be noted in the Termination CRF page. Any ongoing AEs at the time of study withdrawal must be followed until resolution/stabilization. This would not include any subject who became pregnant during study conduct despite contraception. See below for greater detail.

If a subject is withdrawn prematurely from the study for a reason other than those outlined above, this reason must be documented in the Termination CRF page.

In studies that involve more than 1 consecutive dose of study vaccine, a separate event is "**withdrawal of study vaccination**". This event may occur if subjects are expected to receive more than 1 consecutive dose of vaccine as part of study participation. The act of withholding additional study vaccinations is referred to as withdrawal of study vaccination. Subjects may be withdrawn from study vaccination for several reasons including but not limited to: AE related to earlier vaccinations, failure to meet criteria for revaccination (see section 4.4), or pregnancy. **Subjects who are withdrawn from study vaccination should be encouraged to continue in the study for safety follow-up and other procedures as appropriate until the scheduled termination visit.** If the subject is withdrawn from study vaccination(s) due to adverse event, this event must be linked to the withdrawal from vaccination on the AE CRF page.

The sponsor or the investigator (following consultation with the sponsor) has the right to discontinue this study at any time. If the clinical study is prematurely terminated, the investigator is to promptly inform the study subjects and local EC/IRB and should assure appropriate therapy and follow up for the subjects. All procedures and requirements pertaining to the archiving of study documents should be followed. All other study materials (study medication/vaccines, etc.) must be returned to the sponsor.

Any subject who, despite the requirement for adequate contraception, becomes pregnant during the trial will not receive further vaccination but should be encouraged to continue participation in the study. The site should complete a Pregnancy Report CRF (initial report) as soon as possible (see section 6.6.4 further details). If the subject withdraws from the study for any of the above categories except death, the site will obtain permission from the subject to continue to remain in contact with her until the outcome of the pregnancy is known, even if the outcome is not known until after the subject reaches the end of the routine study period.

Subjects will have a blood sample drawn for a complete blood count 7 days after vaccination to establish the absolute neutrophil count. All subjects with an ANC $<1.8 \times 10^9$/L will have the test repeated on a weekly basis until the neutropenia resolves (ANC $\geq 1.8 \times 10^9$/L). If the ANC is $<1.8 \times 10^9$/L at day 21 post vaccination, the subject will be withdrawn from further vaccination. If the subject has an ANC $<0.5 \times 10^9$/L after vaccination (Grade 4 neutropenia), the subject will be withdrawn from further vaccination and be followed up with weekly blood counts until the neutropenia resolves.

Withdrawn subjects will not be replaced.

When a subject is withdrawn or withdraws from the study, the procedures described in section 3.8 Early Termination Visit should be completed if possible.

3.8 Early Termination Visit

When a subject is withdrawn or withdraws from the study, the investigator will notify the sponsor and, when possible, will perform the procedures listed below:

- Collect diary card (as applicable)
- Review the subject's solicited and unsolicited safety data (if collection of these was in progress at the time of study termination)
- Collect vital sign measurements, including respiratory rate, blood pressure, pulse rate, and temperature (orally)
- Perform a pregnancy test for female subjects of childbearing potential
- Collect a blood sample for a complete blood count to assess the absolute neutrophil count

The data for the early termination visit should be recorded in the eCRF for the next clinic visit, a designated early termination visit eCRF, or another form.

4.0 SELECTION OF STUDY POPULATION

4.1 Inclusion Criteria

In order to participate in this study, all subjects must meet ALL of the inclusion criteria described.

1. Males and females of age ≥18 years to ≤45 years.
2. Individuals who, after the nature of the study have been explained to them, have given written consent according to local regulatory requirements.
3. Individuals in good health as determined by the outcome of medical history, physical examination, hematology, renal, bone and liver panels (including negative for agglutination testing of *S. sonnei*), urinalysis and clinical judgment of the investigator.
4. If women of childbearing potential, have a negative pregnancy test prior to study vaccination and willingness to use acceptable contraceptive measures for the entire study duration.
5. Individuals available for follow-up for the duration of the study.
6. Individuals registered with a general practitioner.

4.2 Exclusion Criteria

In order to participate in this study, all subjects must meet NONE of the exclusion criteria described.

1. Individuals with a history of recurrent wheezing, asthma, respiratory allergies, allergic rhinitis, nasal surgery or significant nasal abnormalities (e.g. polyps), and Bell's palsy. Presence of nasal piercings . Symptoms of upper respiratory tract infection within 3 days of intended study vaccination is a temporary exclusion criterion.
2. Individuals unwilling to abstain from medications or other agents that are applied via the nasal route from 24 hours prior to each nasal dosing through to the safety assessment 1 week later.
3. Individuals with behavioral or cognitive impairment or psychiatric disease that, in the opinion of the investigator, may interfere with the subject's ability to participate in the study.
4. Individuals with any progressive or severe neurological disorder, seizure disorder or Guillain-Barré syndrome.
5. Individuals who are not able to understand and to follow all required study procedures for the whole period of the study.
6. Individuals with history of any illness that, in the opinion of the investigator, might interfere with the results of the study or pose additional risk to the subjects due to participation in the study.
7. Individuals with human leukocyte antigen (HLA) -B27 positive and/or with history of reactive arthritis

8. Individuals with known HIV, HBV or HCV infection or HIV related disease, with history of an autoimmune disorder or any other known or suspected impairment /alteration of the immune system, or under immunosuppressive therapy including use of systemic corticosteroids or chronic use of inhaled high-potency corticosteroids within the previous 30 days, or were in chemotherapy treatment within the past 6 months.
9. Individuals with a known bleeding diathesis, or any condition that may be associated with a prolonged bleeding time.
10. Individuals with any serious chronic or progressive disease according to judgment of the investigator (e.g., neoplasm, insulin dependent diabetes, cardiac, renal or hepatic disease).
11. Individuals who have any malignancy or lymphoproliferative disorder.
12. Individuals with history of allergy to vaccine components.
13. Individuals participating in any clinical trial with another investigational product 90 days prior to first study visit or intent to participate in another clinical study at any time during the conduct of this study.
14. Individuals who received any other vaccines within 4 weeks prior to enrollment in this study or who are planning to receive any vaccine within the entire study duration except influenza vaccination, which is not allowed within the period included between 4 weeks before 1st vaccination and 4 weeks after 3rd vaccination
15. Individuals who have received blood, blood products and/or plasma derivatives including parenteral immunoglobulin preparations in the past 12 weeks before randomization.
16. Individuals who are part of study personnel or close family members to the personnel conducting this study or employees of the clinical trial site institution.
17. Individuals with body temperature > 38.0 degrees Celsius within 3 days of intended study vaccination
18. BMI > 30 kg/m2.
19. Individuals with history of substance or alcohol abuse within the past 2 years.
20. Women who are pregnant or breast-feeding or of childbearing age who have not used or do not plan to use acceptable birth control measures, for the duration of the study.
21. Females with history of stillbirth, neonatal loss, (history of planned abortion in not an exclusion), or previous infant with anomaly.
22. Individuals who have a previously ascertained or suspected disease caused by S. sonnei or positive *S. sonnei* serology at screening
23. Individuals who have had household contact with/and or intimate exposure to an individual with laboratory confirmed *S. sonnei*
24. Any condition, which, in the opinion of the investigator may pose an increased and unreasonable safety risk to the subject if participating to the present study

25. Individuals to be enrolled into Cohort C after 10th July 2014 (date of amendment 4) with an absolute neutrophil count lower than 1.8 x 10^9/L at screening.

There may be instances when individuals meet all entry criteria except one that relates to transient clinical circumstances (e.g., body temperature elevation or recent use of excluded medication or vaccine). Under these circumstances, a subject may be considered eligible for study enrollment if the appropriate window for delay has passed, inclusion/exclusion criteria have been rechecked, and if the subject is confirmed to be eligible.

4.3 Criteria for Delay of Vaccination and/or Blood Sampling

After enrollment, subjects may encounter clinical circumstances that warrant a delay in subsequent study vaccination. These situations are listed below. In the event that a subject meets a criterion for delay of vaccination, the subject may receive study vaccination once the window for delay has passed as long as the subject is otherwise eligible for study participation.

- Individuals with a body temperature >38.0°C (>100.4◦F) within 3 days of intended study vaccination.
- Individuals who have received blood, blood products and/or plasma derivatives or any parenteral immunoglobulin preparation in the past 12 weeks.
- Individuals with symptoms of upper respiratory tract infection within 3 days of intended study vaccination.

There are also circumstances under which repeat vaccination is a contraindication in this study. These circumstances include anaphylaxis or severe hypersensitivity reactions following vaccination. If these reactions are to occur, the subject must not receive additional vaccinations but is encouraged to continue in study participation.

4.4 Criteria for Repeat Vaccination in the Study

Prior to receipt of second/additional study vaccination, subjects must be evaluated to confirm that they are eligible for subsequent vaccination. If subjects meet any of the original exclusion criteria or the criteria listed below, they should not receive additional vaccinations.

- In case related/suspected related SAE will occur, further vaccinations will be on-hold while waiting for DSMB recommendation is received by the sponsor (see section 6.9).
- Subjects who develop any new condition which, in the opinion of the investigator, may pose additional risk to the subject if he/she continues to participate in the study.

Subjects who meet any of these criteria must not receive further study vaccinations. However, these subjects should be encouraged to continue study participation, as discussed in section 3.7.

5.0 TREATMENT OF SUBJECTS

All vaccines associated with this study are to be stored separately from other vaccines and medications in a secure location under appropriate storage conditions with temperature monitoring. **All vaccines associated with this study must be checked for expiration date prior to use. Expired vaccines must not be administered to subjects.**

5.1 Study Vaccine(s)

The term ‘study vaccine’ refers to those vaccines provided by the Sponsor, which will be evaluated as part of the study objectives. The study vaccines specific to this study are described below.

NVGH *S. sonnei* (1790GAHB) vaccine

The investigational agent is the NVGH *S. sonnei* vaccine. The vaccine consists of *S. sonnei* 1790-GMMA (200 µg/mL, measured by protein content) adsorbed to Alhydrogel, (0.7 mg $Al^{3+/}$mL) in Tris-buffered saline. The vaccine does not contain any preservative and is available as a liquid formulation in single dose vials with 0.7 mL of injectable solution containing approximately 140 µg of GMMA (as protein content), adsorbed onto 0.49 mg Al^{3+}.

It should be stored at +2/+8 C. Extractable volume is 0.6 mL.

The vaccine is going to be administered intramuscularly, intradermally and intranasally. Vaccinations will be administered at different antigen dosages obtained by bed-side mixing**.** Bed-side mixing instructions will be provided to the investigator and will be located in the investigator site file. Refer to Section 5.3 for additional instructions.

Intradermal administration

For the intradermal injections, a syringe will be used. The volume administered will be 0.05 mL.

ID 0.1 µg: Following dilution, each dose contains 0.1 µg of GMMA total protein and 0.035 mg of Al^{3+}.

ID 1 µg: Following dilution, each dose contains 1 µg of GMMA total protein and 0.035 mg of Al^{3+}.

ID 10 µg: Each dose contains 10 µg of GMMA total protein and 0.035 mg of Al^{3+}.

Intranasal administration

For the IN injections, a mucosal atomization device will be used for dose spraying (manufactured by Wolfe Tory Medical, Inc. Utah and distributed by Cavendish Scott Ltd, England). The total volume administered, preferentially half in each nostril, will be 0.4 mL.

IN 5 µg: Following dilution, each dose contains 5 µg of GMMA total protein and 0.28 mg of Al^{3+}.

IN 20 µg: Following dilution, each dose contains 20 µg of GMMA total protein and 0.28 mg of Al^{3+}.

IN 80 µg: Each dose contains 80 µg of GMMA total protein and 0.28 mg of Al^{3+}.

Intramuscular administration

IM 5 µg: Each dose contains 5 µg of GMMA total protein and 0.35 mg of Al^{3+}.

For each route, three vaccinations will be administered, 28 days apart. IM and ID vaccinations will be administered preferentially in the deltoid of the non-dominant arm. IN vaccination will be administered in both nostrils. Bed-side mixing instructions will be provided to the investigator and will be located in the investigator site file. Refer to Protocol Section 5.3 for additional instructions.

Control agent (Placebo)

The control treatment is a placebo. The placebo is going to be composed of Alhdyrogel (0.7 mg Al^{3+}/mL) in Tris–buffered saline. The placebo is available in single dose vials with 0.7 ml of injectable solution containing 0.49 mg of Al^{3+}. Extractable volume is 0.6 mL. Three placebo doses, 28 days apart, will be administered as follows:

1. 0.05 mL intradermally, containing 0.035 mg of Al^{3+}
2. 0.4 mL intranasally, containing 0.28 mg of Al^{3+} 0.5 mL intramuscularly, containing 0.35 mg of Al^{3+}
3. 0.5 mL intramuscularly, containing 0.35 mg of Al^{3+}

IM and ID vaccinations will be administered preferentially in the deltoid of the non-dominant arm. IN vaccination will be administered in both nostrils.

No other concomitant vaccines or treatments will be used as part of study procedures.

5.2 Non-Study Vaccines

The term 'non-study vaccine' refers to those vaccines which will be intentionally given to study subjects but not formally included in the analysis of study objectives.

Not applicable to this study.

5.3 Vaccines Preparation and Administration

The investigator or designee will be responsible for oversight of the administration of vaccine to subjects enrolled in the study according to the procedures stipulated in this study protocol. All vaccines will be administered only by personnel who are qualified to perform that function under applicable local laws and regulations for the specific study site.

The bedside mixing procedure to obtain the required dosage of 1790GAHB vaccine will be performed by a trained site staff qualified to perform that function under applicable local laws and regulations for the specific study site.

PRECAUTIONS TO BE OBSERVED IN ADMINISTERING STUDY VACCINE:

Prior to vaccination, subjects must be determined to be eligible for study vaccination and it must be clinically appropriate in the judgment of the investigator to vaccinate. Eligibility for vaccination prior to first study vaccine administration is determined by evaluating the entry criteria outlined in protocol section 4.1 through 4.2.

Eligibility for subsequent study vaccination is determined by following the criteria outlined in sections 4.3 and 4.4.

Study vaccines should not be administered to individuals with known hypersensitivity to any component of the vaccines.

Standard vaccinations practices are to be observed and care should be taken to administer the injections intramuscularly. Before administering vaccine, the vaccination site is to be disinfected with a skin disinfectant (e.g., 70% alcohol). Allow the skin to dry. **DO NOT inject intravascularly**.

As with all injectable vaccines, trained medical personnel and appropriate medical treatment should be readily available in case of anaphylactic reactions following vaccine administration. For example, epinephrine 1:1000, diphenhydramine, and/or other medications for treating anaphylaxis should be available.

5.4 Prior and Concomitant Medications and Vaccines

All medications, vaccines and blood products taken or received by the subject within 4 weeks prior to the start of the study are to be recorded on the Prior and Concomitant Medications eCRF. The use of antipyretics and/or analgesic medications within 24 hours prior to vaccination must be identified and the reason for their use (prophylaxis versus treatment) must be described in the source documents and Concomitant Medications eCRF. Medications taken for prophylaxis are those intended to prevent the onset of symptoms. Medications taken for treatment are intended to reduce or eliminate the presence of symptoms that are present.

Concomitant medications include all medications (including vaccines) taken by/administered to the subject at and after enrollment and must be documented on the Concomitant Medications eCRF.

When recording concomitant medications/vaccines, they should be checked against the study entry and continuation criteria in sections 4.1 through 4.4 to ensure that the subject should be enrolled/continue in the study.

5.5 Vaccine Supply, Labeling, Storage, and Tracking

The sponsor will ensure the following:

- supply the study vaccines and placebo
- appropriate labeling of study vaccines and placebo and provided that complies with the legal requirements of the country where the study is to be performed

The investigator must ensure the following:

- acknowledge receipt of the study vaccines and placebo by a designated staff member at the site, including confirmation that the vaccines:
  - were received in good condition
  - remained within the appropriate temperature range during shipment from the sponsor to the investigator's designated storage location
  - have been confirmed by the sponsor as authorized for use
- proper storage of the study vaccines and placebo, including:
  - storage in a secure, locked, temperature-controlled location
  - proper storage according to the instructions specified on the labels
  - appropriate record keeping and inventory of the study vaccines, including regular documentation of adequate storage temperature
- appropriate use of the study vaccines and placebo, including:
  - dilution in accordance to bed-side mixing procedure and documentation
  - use only in accordance with the approved protocol
  - proper handling, including confirmation that the vaccine has not expired prior to administration
  - appropriate documentation of administration of vaccines to study subjects including:
    - date, dosage, batch/serial numbers, expiration dates, unique identifying numbers assigned to subjects and study vaccines, and time of vaccine administration. This information will be maintained in an accountability log that will be reviewed by the site monitor.
    - proper reconciliation of all study vaccines received from the sponsor. Reconciliation is defined as maintaining records of which and how many vaccines were received, which vaccines (and volume thereof) were administered to subjects, which vaccines were destroyed at the site, and which vaccines were returned to the sponsor, as applicable.
- proper adherence to the local institutional policy with respect to destruction of study vaccines.
- complete record keeping of vaccine dilution, use, wastage, return or destruction, including documentation of:
  - copy of the site's procedure for destruction of hazardous material
  - number of doses destroyed, date of destruction, destruction code (if available), method of destruction, and name of individual performing destruction

Vaccines that have been stored differently from the manufacturer's indications **must not** be used unless the sponsor provides written authorization for use. In the event that the use

cannot be authorized, the sponsor will make every effort to replace the vaccine supply. All vaccines used in conjunction with this protocol must be stored separately from normal hospital/practice stocks to prevent unintentional use of study vaccines outside of the clinical trial setting.

Monitoring of vaccine accountability and dilution will be performed by the unblinded study monitor during site visits and at the completion of the trial.

At the conclusion of the study, and as appropriate during the course of the study, the investigator must return all unused study vaccines, packaging and supplementary labels to the sponsor or destroy at site, as required by local regulations.

6.0 MEASUREMENTS

6.1 Appropriateness of Measurements

The measures of immunogenicity used in this study i.e. IgG enzyme-linked immunosorbent assay (ELISA) in sera and IgA ELISA on stool samples extract, are standard, widely used and generally recognized as reliable, accurate, and relevant (able to describe the quality and extent of the immune response). The ELISA methodology used in this study has been adopted based on scientific consensus and has been deemed appropriate to describe the immune response against *Shigella sonnei* GMMA in this study.

The measures of safety used in this study are routine clinical and laboratory procedures. They include a close vigilance for, and stringent reporting of, selected local and systemic adverse events routinely monitored in vaccine clinical trials as indicators of reactogenicity in adults. Safety data will be documented in a Diary card and entered into an eCRF.

6.2 Demographics, Medical History and Physical Examination

Prior to study enrollment, demographic data will be collected from the subject, including: age, gender, race, body mass index. Race is collected as differences in response to medical products have been observed in racially and ethnically distinct subgroups. These differences may be attributable to intrinsic factors (e.g., genetics, metabolism, elimination), extrinsic factors (e.g., diet, environmental exposure, sociocultural issues), or interactions between these factors.

Medical history will also be collected, including but not limited to any medical history that may be relevant to subject eligibility for study participation such as prior vaccinations, concomitant medications, and previous and ongoing illnesses or injuries. Relevant medical history can also include any medical history that contributes to the understanding of an adverse event that occurs during study participation, if it represents an exacerbation of an underlying disease/preexisting problem.

A general physical examination is to be performed by a qualified health care practitioner and will include, at a minimum, evaluation of the following organ systems and collection of vital signs: respiratory system (respiratory rate) and cardiovascular system (systolic/diastolic blood pressure, heart rate, and pulse rate). “Qualified health care practitioner” refers to any licensed health care professional who is permitted by institutional policy to perform physical examinations and who is identified within the site’s roles and responsibilities log.

As cardiovascular and respiratory safety assessment should be considered for new vaccines in appropriate animal models [11], particular monitoring of these activities should be incorporated in the design of the clinical studies. Therefore the evaluation of the respiratory rate and systolic/diastolic blood pressureheart rate and pulse rate will be repeated before and during observation period after each vaccination (approximately 30 and 120 minutes).

These data will be written in the source documents and entered into an eCRF.

6.3 Immunogenicity Measurements

The measure of immunogenicity used in this study is an ELISA against *S. sonnei* OAg. The serologic assays will be conducted on serum samples and will be performed at Novartis Vaccines, Clinical Serology Laboratory, Marburg, (Germany) or a delegated laboratory.

Additional exploratory analyses aimed at characterizing the mucosal immunity induced by the vaccine by evaluating IgA on stool samples extract will be performed. Testing will be conducted by qualified and certified laboratories Please refer to the Laboratory Manual in the investigator site file for details.

For reference of visits the measurements are taken, refer to section 3 and to the Serology Manual.

Immunogenicity data will be provided by electronic data transfer to NVx (refer to section 9).

6.4 Efficacy Measurements

This study has no efficacy measurements.

6.5 Solicited Safety Measurements

These data will be entered into a Diary card, and following reconciliation, into eCRF. Please see sections 3.2.5.3, 3.4.1 and section 8.1 for more detail.

The term “reactogenicity” refers to selected signs and symptoms (“adverse events”) occurring in the hours and days following a vaccination, to be collected by the subject for 7 consecutive days, using a pre-defined checklist in a diary card (i.e., solicited adverse events).

Solicited local adverse events following IM vaccination: erythema, induration and pain at injection site.

Solicited local adverse events following ID vaccination: erythema, induration, pain and swelling at injection site.

Solicited local adverse events following IN vaccination: facial edema, nasal pain and rhinorrhea.

Solicited systemic adverse events: headache, arthralgia, chills, fatigue, malaise, myalgia, and fever measured orally.

Other solicited reactions: Use of analgesics/antipyretics, body temperature

The severity of solicited local and systemic adverse event will be graded as below:

Table 6.5-1: Grading of Solicited Local, Systemic Adverse Events and Other Indicator of Reactogenicity for All Subjects

Solicited adverse events	Grade 0 Absent	Present - Grading of Severity*		
		Grade 1 Mild	Grade 2 Moderate	Grade 3 Severe
**Injection site Erythema** (Captured as measurements in millimeters)	1-24 mm	25-50 mm	51-100 mm	> 100 mm
**Injection site Induration** (Captured as measurements in millimeters)	1-24 mm	25-50 mm	51-100 mm	> 100 mm
**Injection site Pain**	No pain	Present but does not interfere with activity	Interferes with activity	Prevents daily activity
**Injection site Swelling**	1-24 mm	25-50 mm	51-100 mm	> 100 mm
**Facial edema**	No facial edema	Present but does not interfere with activity	Interferes with activity	Prevents daily activity
**Nasal pain**	No nasal pain	Present but does not interfere with activity	Interferes with activity	Prevents daily activity
**Rhinorrhea**	No rhinorrhea	Present but does not interfere with activity	Interferes with activity	Prevents daily activity
**Headache**	No headache	Present but does not interfere with activity	Interferes with activity	Prevents daily activity
**Arthralgia**	No arthralgia	Present but does not interfere with activity	Interferes with activity	Prevents daily activity
**Chills**	None	Present but does not interfere with activity	Interferes with activity	Prevents daily activity
**Fatigue**	No fatigue	No interference with activity	Some interference with activity	Significant; prevents daily activity
**Malaise**	No malaise	No interference with activity	Some interference with activity	Significant; prevents daily activity
**Myalgia**	No myalgia	Present but does not interfere with activity	Interferes with activity	Prevents daily activity
**Fever as a**	≤ 37.9 °C	≥ 38.0 – 38.9°C	≥ 39.0 – 39.9°C	≥ 40.0°C
**Body temperature** (Captured as measurements in degrees Celsius)	Oral Temperature: <35.5 °C to ≥38.0 °C			
**Use of analgesics/antipyretics**	Categorized as "yes" or "no"			

*eCRF instructions will clarify what is meant for interference with activity. Investigators will be trained to explain to subjects

The study staff must review the diary card with the subject at the following visit (see section 3.2.5) and must directly record the solicited local and systemic adverse events, and other solicited reactions on the appropriate Local and Systemic Reactions eCRF. As described in Section 3.4.1, all solicited adverse events that are legible must be recorded verbatim in the eCRFs, even if the values do not appear to be plausible.

If a solicited local or systemic adverse event continues beyond day 7 after vaccination, it will also be recorded as an Adverse Event on the Adverse Events eCRF.

6.6 Unsolicited Safety Measurements

6.6.1 Adverse Events

An adverse event (AE) is defined as any untoward medical occurrence in a subject or clinical investigation subject administered a pharmaceutical product at any dose that does not necessarily have to have a causal relationship with this treatment. Therefore, an AE can be any unfavorable and unintended sign (including an abnormal laboratory finding, for example), symptom, or disease temporally associated with the use of an investigational product, whether or not considered related to the investigational product. This definition includes intercurrent illnesses or injuries and exacerbation of pre-existing conditions.

NOTE: Every effort should be made by the investigator to evaluate new safety information reported by a subject (solicited and unsolicited AEs) for an underlying diagnosis and to capture this diagnosis as the event in the AE page. In other words, the practice of reporting only symptoms (e.g., “cough” or “ear pain”) are better reported according to the underlying cause (e.g., “asthma exacerbation” or “otitis media”).

All AEs will be monitored until resolution or, if the AE becomes chronic, a cause identified. If an AE is unresolved at the conclusion of the study, a clinical assessment will be made by the investigator and medical monitor whether continued follow-up of the AE is warranted.

The severity of events reported on the Adverse Events eCRF will be determined by the investigator as:

Mild:	transient with no limitation in normal daily activity.
Moderate:	some limitation in normal daily activity.
Severe:	unable to perform normal daily activity.

The relationship of the study treatment to an AE will be determined by the investigator based on the following definitions:

<u>1. Not Related</u>

The AE is not related to an investigational vaccine if there is evidence that clearly indicates an alternative explanation. If the subject has not received the vaccine, the timing of the exposure to the vaccine and the onset of the AE are not reasonably related in time, or other facts, evidence or arguments exist that reasonably suggest an alternative explanation, then the AE is not related.

<u>2. Possibly Related</u>

The administration of the investigational vaccine and AE are considered reasonably related in time and the AE could be explained by exposure to the investigational vaccine or by other causes.

<u>3. Probably Related</u>

Exposure to the investigational vaccine and AE are reasonably related in time and no alternative explanation has been identified.

The relationship of the study treatment to an unsolicited AE will be determined by the investigator. Solicited AEs will not be evaluated for relationship to study vaccine and severity of solicited AEs is defined as described in section 6.5.

Adverse events will also be evaluated by the investigator for the co-existence of any of the other following conditions:

- "Medically attended adverse event": an adverse event that leads to an unscheduled visit to a healthcare practitioner.
- "New onset of chronic disease": an adverse event that represents a new diagnosis of a chronic medical condition that was not present or suspected in a subject prior to study enrollment.
- "New onset of autoimmune disorder": an adverse event that represents a new diagnosis of an autoimmune disease that was not present or suspected in a subject prior to study enrollment.

Please note: any solicited adverse event that meets any of the following criteria must also be entered as an adverse event on the Adverse Event eCRF:

- Solicited local or systemic adverse event leading to a "medically attended adverse event".
- Solicited local or systemic adverse event leading to the subject withdrawing from the study or the subject being withdrawn from the study by the investigator.
- Solicited local or systemic adverse event lasting beyond *7* days' duration.
- Solicited local or systemic adverse events that lead to subject withdrawal from study vaccination.
- Solicited local or systemic adverse event that otherwise meets the definition of a serious adverse event (see section 6.6.2).

6.6.1.1 Adverse Events of Special Interest

Adverse events of special interest (AESIs) are predefined adverse events that will be specifically highlighted to the investigator and will be summarized separately at the end of the study.

In this protocol, the reactive arthritis is an AESI. Reactive arthritis is defined as a non-purulent joint inflammation that develops in response to an infection in another party of the body. Since the inflammation is triggered by a previous condition, it is termed "reactive" [12]. If reactive arthritis is caused by an auto immune response, there is at least a possibility that it could be initiated by vaccination of susceptible people (i.e. HLA-B27 positive individuals) with 1790GAHB vaccine. Therefore as part of the screening, subjects will be tested for human leukocyte antigen (HLA) B27. Subjects positive for the gene coding for this protein, along with those with a history of reactive arthritis, will be excluded.

The reactive arthritis will not meet the definition of a SAE automatically and will be categorized as SAE only if meeting the criteria for seriousness through some feature.

6.6.2 Serious Adverse Events

A serious adverse event (SAE) is defined as any untoward medical occurrence that at any dose results in one or more of the following:

- Death.
- Is life-threatening (i.e., the subject was, in the opinion of the investigator, at immediate risk of death from the event as it occurred); it does not refer to an event which hypothetically might have caused death if it were more severe.
- Required or prolonged hospitalization.
- Persistent or significant disability/incapacity (i.e., the event causes a substantial disruption of a person's ability to conduct normal life functions).
- Congenital anomaly/or birth defect.
- An important and significant medical event that may not be immediately life threatening or resulting in death or hospitalization but, based upon appropriate medical judgment, may jeopardize the subject or may require intervention to prevent one of the other outcomes listed above.

Adverse events which do not fall into these categories are defined as **non-serious**.

It should be noted that a severe adverse event need not be serious in nature and that a serious adverse event need not, by definition, be severe.

Serious adverse events will be captured both on the VSAE form as well as on the AE eCRF. All SAEs will be evaluated by the investigator for relationship of the event to study vaccine. SAEs that are judged to be possibly or probably related to the study vaccine should be reported to the sponsor as related (i.e., suspected) events.

The relationship of the study treatment to an SAE will be determined by the investigator based on the following definitions:

<u>1. Related/suspected</u>

The SAE is judged by the investigator to be possibly or probably related to the study vaccine on the AE CRF page (see section 6.6.1).

<u>2. Not Related</u>

The SAE is not related if exposure to the study vaccine has not occurred, **or** the occurrence of the SAE is not reasonably related in time, **or** the SAE is considered unlikely to be related to use of the study vaccine, i.e., there are no facts (evidence) or arguments to suggest a causal relationship.

The relationship of the study vaccine to an SAE will be determined by the investigator.

In addition, SAEs will be evaluated by the sponsor or designee for "expectedness." An unexpected AE is one that is not listed in the current Summary of Product Characteristics or the Investigator's Brochure or an event that is by nature more specific or more severe than a listed event.

In addition, a pre-existing event or condition that results in hospitalization should be recorded on the Medical History eCRF. If the onset of an event occurred before the subject entered the trial (e.g., any pre-planned hospitalization for conditions like cosmetic treatments or for non-emergency routine visits for a pre-existing condition), the hospitalization would not lead to an AE being classified as serious unless, in the view of the investigator, hospitalization was prolonged as a result of participation in the clinical trial or was necessary due to a worsening of the pre-existing condition.

6.6.3 Methods for Assessing and Recording AEs and SAEs

The period of observation for AEs extends from the time the subject signs informed consent until he or she completes the specified 6-month safety follow-up period following 3rd vaccination or terminates the study early (whichever comes first). AEs occurring after the informed consent form is signed but prior to receiving study vaccine/product will be documented as an adverse event and recorded on the Adverse Events eCRF and within source documents. However, AEs occurring prior to receipt of any study vaccine will be analyzed separately from "treatment emergent" AEs (AEs occurring after administration of the first study vaccine).

All AEs meeting criteria for reporting, regardless of severity, will be monitored by the investigator until resolution or stabilization. All subjects experiencing AEs - whether considered associated with the use of the study vaccine or not - must be monitored until symptoms subside and any abnormal laboratory values have returned to baseline, or until there is a satisfactory explanation for the changes observed, or until death, in which case a full pathologist's report should be supplied, if possible. All findings must be reported on an Adverse Events CRF and on the VSAE form, if necessary, which is part of the Investigator Site File. All findings in subjects experiencing AEs must be reported also in the subject's medical records.

All SAEs which occur during the course of the trial, whether considered to be associated with the study vaccination or not, must be reported **within 24 hours of the site becoming aware of the event** by telephone or fax to Novartis. Contact details for submitting SAEs to Novartis or its designee and instructions for completion of documentation will be provided in a handout located in the Investigator Site File.

All SAEs are also to be documented on the Adverse Events eCRF. Any medication or other therapeutic measures used to treat the AE will be recorded on the appropriate eCRF(s) in addition to the outcome of the AE.

After receipt of the initial report, representatives of Novartis will contact the investigator if it is necessary to obtain further information for assessment of the event.

All SAEs must be reported by the sponsor to site corresponding IRB/EC and/or applicable regulatory authorities in accordance with institutional policy/regulatory requirements.

NVGH or its designee must also comply with the applicable regulatory requirement(s) related to the reporting of unexpected serious and non-serious adverse vaccine reactions (also referred to as “SUSARs”) to the regulatory authority(ies) and the IRB/EC. If a SUSAR or other safety signal relating to use of one of the study vaccines is reported to NVGH or its designee, the sponsor will communicate the information to the EC/IRB and other relevant authorities.

Post-Study Events

Any AE that occurs outside of the protocol-specified observation period or after the end of the study but considered to be caused by the study vaccine must be reported to NVGH. These AEs will be processed by the Novartis Pharmacovigilance group. Instructions for how to submit these AEs will be provided in a handout in the Investigator Site File.

6.6.4 Pregnancies

To ensure subjects’ safety, each pregnancy in a subject on study vaccine must be reported to NVGH within 24 hours of the site learning of its occurrence. If the subject agrees to submit this information, the pregnancy must be followed to determine outcome, including spontaneous or voluntary termination, details of the birth, and the presence or absence of any birth defects, congenital abnormalities, or maternal and/or newborn complications. This follow-up should occur even if intended duration of safety follow-up for the study has ended.

Pregnancy data must be recorded on a Pregnancy Report CRF (initial report) and Pregnancy Follow-Up CRF (outcome report) and reported to NVGH. Contact details for submitting the case report forms will be described in the Investigator Site File.

Any pregnancy outcome meeting the definition of a SAE (see section 6.6.2) must also be reported on the VSAE Report Form.

6.7 Safety Laboratory Measurements

For list of safety laboratory measurement, refer to Table 2.

Safety laboratory measurement will be evaluated at the screening, 7 days after 1st vaccination (at Visit 2), 28 days after 2nd vaccination (Visit 4) and 28 days after 3rd vaccination (Visit 5) (see section 3.5.4). To ensure that the measurement is adequate, fasting before testing will be required.

Further safety blood sample may be collected, should the investigator consider it necessary to better evaluate the subject conditions or a lab abnormality .

Safety laboratory assessments will be performed at the site laboratory, and results of these tests will be recorded in the source documents and in the e-CRFs.

6.8 Other Measurements

Not Applicable.

6.9 Data Safety and Monitoring Board

An independent, external Data Safety and Monitoring Board (DSMB) will be established to support the decision to proceed with the clinical testing of progressively higher IN and ID dosages. Enrollment of new ID and IN dosages will be on hold until the DSMB recommendation has been obtained.

The DSMB will receive a summary of all safety data (solicited local and systemic reactions, unsolicited adverse events and SAE) and listings of clinically significant modifications in hematology, blood chemistry and urinalysis test values obtained during one week follow-up post-first vaccination with the lower dose. Based on evaluation of the safety data, the DSMB will make a recommendation to the sponsor, as to whether the next cohort should be vaccinated with higher antigen concentration or not, as follows:

1. Independent dose escalation recommendations should be made for IN and ID vaccination routes.

2. In case no related/suspected SAE and no at least possibly related solicited and unsolicited severe/grade 3 AE occurred during the one week follow-up post-first vaccination with lower dose, DSMB will recommend that the second cohort will be vaccinated either with *S. sonnei* 1790GAHB next dose or placebo. With the same approach subsequent cohorts will be vaccinated with progressively higher antigen dosages.

3. In case of occurrence of related/suspected SAE and/or at least possibly related solicited and unsolicited severe/grade 3 AEs, DSMB will evaluate all safety data and will provide recommendation on whether the study (of the cohort) should be halted or continued as planned.

DSMB will receive related/suspected related SAE occurring throughout out the study. In case related/suspected related SAE will occur, enrollment of new subjects and further vaccinations will be on-hold while waiting for DSMB recommendation is received by the sponsor (see section 4.4).

The composition of DSMB and the details of all relevant procedures will be documented in the DSMB Charter.

7.0 ENDPOINTS AND STATISTICAL ANALYSES

7.1 Endpoints

7.1.1 Primary Endpoint(s)

The primary safety endpoints will include:

- Numbers of subjects with deviations from normal values of hematological, haematochemical blood tests and urinalysis after vaccination.
- Numbers of subjects with solicited local and systemic reactions during 7 days following each vaccination.
- Numbers of subjects with reported unsolicited adverse events during 28 days following each vaccination.
- Number of subjects with reported SAEs throughout the study duration.
- Number of subjects with reported reactive arthritis (AE of special interest (AESI).

7.1.2 Secondary Immunogenicity Endpoints

The immunogenicity endpoints will include:

a. IgG Geometric mean concentrations (GMCs) pre-vaccination (day 1), 28 days after 1st vaccination, 28 days after 2nd vaccination, 28 and 168 days after 3rd vaccination as determined by ELISA, and applicable geometric mean ratios between post- and pre-vaccination samples.

b. Number of subjects with seroresponse for anti- LPS *S. sonnei* at 28 days after 1st vaccination, 28 days after 2nd vaccination, and 28 and 168 days after 3rd vaccination

Seroresponse is aimed to define a significant increase in post vaccination samples based on the biological performance of this specific serology assay and it is defined as:

If half of the baseline value is greater than 25 EU then an increase of at least 50% in the post-vaccination sample as compared to baseline [i.e. ((Post-vac minus baseline)/baseline)100% ≥ 50%]

If half of the baseline value is less or equal to 25 EU then an increase of at least 25 EU in the post-vaccination sample as compared to baseline [i.e. (post-vac minus baseline) ≥ 25 EU]

c. Number of subjects with high seroresponse for anti-LPS *S. sonnei* at 28 days after 1st vaccination, 28 days after 2nd vaccination, and 28 and 168 days after 3rd vaccination

High seroresponse is defined as a post vaccination titer ≥ X anti-LPS serum IgG units in the Novartis ELISA that correspond to a titer of 1:800 in the ELISA method used by Cohen et al. (1989 J. Clin. Microbiol. 27:162). To determine the value for ‘X’ the Novartis anti-LPS ELISA will be calibrated against the Cohen ELISA.

Other assays might be performed to further characterize the immune response to the study vaccine.

7.1.3 Secondary Efficacy Endpoints

Not applicable.

7.1.4 Safety Endpoints

See section 7.1.1

7.1.5 Other Endpoints

Not applicable.

7.1.6 Exploratory Endpoints

The measures of the exploratory immunogenicity outcome, (i.e., the fecal anti-LPS sIgA), will include:

- Fecal sIgA GMCs pre-vaccination (day 1), 28 days after 1st vaccination, 28 days after 2nd vaccination, 28 and 168 days after 3rd vaccination, as determined by ELISA, and applicable geometric mean ratios between post- and pre-vaccination samples.

Fecal sIgA will be assessed in the stool specimens of at least one cohort.

Frequencies and fold increases of memory B cells specific for the OAg of the vaccine and its carrier (GMMA) and antibody secreting cells B cells (plasma blasts) at relevant timepoints.

7.2 Success Criteria

This is a Phase 1 trial and there is no pre-defined success criterion.

7.2.1 Success Criteria for Primary Objectives

7.2.2 Success Criteria for Secondary Immunogenicity Objectives

Not applicable.

7.2.3 Success Criteria for Secondary Efficacy Objectives

Not applicable.

7.2.4 Success Criteria for Safety Objectives

Not applicable.

7.3 Analysis Sets

7.3.1 All Enrolled Set

All screened subjects who provide informed consent and provide demographic and/or baseline screening assessments, regardless of the subject's randomization and treatment status in the trial and received a subject ID.

7.3.2 Exposed Set

All subjects in the Enrolled Population who receive a study vaccination.

7.3.3 Full Analysis Set (FAS) Efficacy/Immunogenicity Set

All subjects in the Enrolled Population who:

- receive a study vaccination AND provide at least one immunogenicity data at relevant time points.

FAS populations will be analyzed "as randomized" (i.e., according to the vaccine a subject was designated to receive, which may be different from the vaccine the subject actually received).

The FAS will be the primary analysis set for the immunogenicity objective.

7.3.4 Per Protocol (PP) Population, Efficacy/Immunogenicity Set

All subjects in the FAS Immunogenicity Population who:

- Are not excluded due to reasons (see section 7.3.8) defined prior to unblinding

PPS are subsets of FAS and should be always defined even if the objectives do not require it.

Examples for subjects excluded due to other reasons than major protocol deviations are:

- Subjects who withdrew informed consent

Exclusions need to be considered by objective/time point, i.e., sometimes not all data of a subject but only part of the subject's data will be removed from the PPS analysis.

7.3.5 Safety Set

Safety Set (solicited adverse events and other solicited reactions)

All subjects in the Exposed Population who:

- Provide post vaccination reactogenicity data

Safety Set (unsolicited adverse events)

- All subjects in the Exposed Population who:
- Have post-vaccination unsolicited adverse event records

Safety Set (overall)

All subjects in the Exposed Population who:

- Have either post-vaccination adverse event or reactogenicity records

Subjects will be analyzed as "treated" (i.e., according to the vaccine a subject received, rather than the vaccine to which the subject may have been randomized).

7.3.6 Other Analysis Sets

Not applicable.

7.3.7 Subgroups

Not applicable.

7.3.8 Protocol Deviations

A protocol deviation is any change, divergence, or departure from the study design or procedures of a study protocol. An exclusion refers to a protocol deviation that is used to remove data from an analysis population at the time of analysis. Relevant protocol deviations will be defined as exclusionary from the analysis according to protocol objectives and endpoints, which will be specified in the statistical analysis plan.

Any deviation that affects the subject's rights, safety, or well- being and/or the completeness, accuracy and reliability of the study data constitutes a major protocol deviation. Changes or alterations in the conduct of the trial which do not have a major impact on the subject's rights, safety or well-being, or the completeness, accuracy and reliability of the study data are considered minor protocol deviations. Major and minor deviations will be reviewed to determine the final list of deviations that will be used for exclusion from the analysis set(s). This will be defined in the statistical analysis plan prior to unblinding.

The following deviations are considered major:

- A subject received incorrect study vaccine or dose of study vaccine.
- A subject met withdrawal criteria during the study but was not withdrawn.
- A subject received an excluded medication or vaccine.
- A subject whose post-vaccination blood draw falls outside the window defined in the Time and Events Table.
- A subject received vaccine outside the window defined in the Time and Events Table.
- A subject was enrolled but does not meet the protocol's eligibility criteria.
- A subject with no safety data.
- Inadvertent loss of samples or data that support the analysis of primary or key objectives.

- Failure to obtain informed consent prior to initiation of study-related procedures.
- Falsifying research or medical records.

Subjects who terminate study participation prematurely for reasons such as withdrawal of consent, adverse event (including death), and administrative reason do not represent protocol deviations, nor are the missing assessments that should otherwise have been collected for that subject later in the study considered protocol deviations.

Pre specified reasons for delay or cancellation of study vaccination as reflected in sections 4.3 and 4.4 do not constitute protocol deviations.

All protocol deviations will be classified into major and minor. Major protocol deviations will be summarized by vaccine, center (overall) and grouped into the different categories as defined above. The site monitor will keep the investigator informed of minor and major protocol deviations, so that the investigator can comply with reporting these deviations to the local EC/IRB according to their institutional policy.

Prior to unblinding, designated staff at the Sponsor will develop a memo that describes the selected deviations that are identified as exclusions from analysis populations. This memo will be included in the trial master file.

7.4 Analysis Plan

7.4.1 Analysis of Demographic and Baseline Characteristics

Descriptive statistics (mean, standard deviation, median, minimum and maximum) for, age, height and weight at enrollment will be calculated by overall and by vaccine group.

Distributions of subjects by sex and race will be summarized.

7.4.2 Analysis of Primary Objectives

7.4.2.1 Statistical Hypotheses for Primary Objectives

This Phase 1 safety and immunogenicity trial is aimed to descriptively evaluate the safety and immunogenicity profiles of the study vaccines. No specific hypotheses are tested in this trial.

7.4.2.2 Analysis Populations for Primary Objectives

The FAS will be the primary analysis set for the primary immunogenicity objective.

7.4.2.3 Statistical Methods for Primary Objectives

Analysis of continuous variables

The ELISA concentrations will be logarithmically transformed (base10) (to fulfil the normal distribution assumption). GMC will be calculated, with their associated two-sided 95% CIs, by exponentiating the corresponding log-transformed means and their 95% CI.

Additionally, within-subject GMRs will be computed for GMTs/GMCs at one month after first, second and third vaccination and at 6 months after third vaccination versus baseline (day 1). The GMRs and 95% CIs will be constructed by exponentiating the mean within-subject differences in log-transformed titers and the corresponding 95% CIs.

Analysis of binary variables

The number and percentages of subjects with seroresponse and with high seroresponse in ELISA concentrations from baseline will be summarized. Two-sided 95% Clopper-Pearson CIs for the percentages will be computed.

Titers below the limit of detection will be set to half that limit for the purposes of analysis. Missing values of immunogenicity will be excluded from analyses (i.e. complete-case analysis) since they are considered missing completely at random, i.e. not informative and with no impact on inferences.

7.4.2.4 Sample Size and Power Considerations of Primary Objectives

No formal statistical sample size and power computations are performed since the objectives of the study are to descriptively assess the safety and immunogenicity of the NVGH 1790GAHB vaccine.

7.4.2.5 Analysis of Safety Objectives

7.4.2.5.1 Analysis of Extent of Exposure

The frequencies of subjects receiving vaccinations will be summarized, by visit and by vaccine group. Data will be tabulated for the overall Safety Set.

7.4.2.5.2 Analysis of Solicited Local and Systemic Adverse Events and Other Reactions

Solicited adverse events will be summarized according to defined severity grading scales.

Frequencies and percentages of subjects experiencing each adverse event will be presented for each symptom severity. Summary tables showing the occurrence of any local or systemic adverse event overall and at each time point will also be presented.

Post-vaccination solicited adverse events reported from day 1 to day 7 will be summarized for the intervals day 1-3, day 4-7, day 1-7 by maximal severity, by vaccine group and by vaccination, excluding the 120 minutes measurement, which will be summarized separately. The severity of solicited local adverse events, namely, injection-site erythema and induration will be summarized according to categories based on linear measurement as described in Table 6.5-1.

Injection site pain and systemic reactions occurring up to7 days after each vaccination will be summarized according to “mild”, “moderate” or “severe”.

Each solicited local and systemic adverse event will also be further summarized as “none” versus “any”.

Implausible measurements (for further definition see analysis plan) will be left out of the analysis.

Use of antipyretics and analgesics will be summarized by frequency of subjects reporting use.

Body temperature will be summarized by 0.5 °C increments from 36.0°C up to ≥40°C and will be broken down according by route of measurement.

7.4.2.5.3 Analysis of Spontaneously Reported Adverse Events

All the adverse events occurring during the study, judged either as probably related, possibly related, or not related to vaccination by the investigator, will be recorded.

The original verbatim terms used by investigators to identify adverse events in the CRFs will be mapped to preferred terms using the MedDRA dictionary. The adverse events will then be grouped by MedDRA preferred terms into frequency tables according to system organ class. All reported adverse events, as well as adverse events judged by the investigator as at least possibly related to study vaccine, will be summarized according to system organ class and preferred term within system organ class. These summaries will be presented by vaccination group and by interval of study observation. When an adverse event occurs more than once for a subject, the maximal severity and strongest relationship to the vaccine group will be counted.

Separate summaries will be produced for the following categories:

- serious adverse events
- adverse events that are possibly or probably related to vaccine
- adverse event leading to withdrawal from the study
- after any vaccination and by vaccination

Data listings of all adverse events will be provided by subject. In addition, adverse events in the categories above will be provided as listed data.

7.4.2.5.4 Analysis of Safety Laboratory Values

All laboratory safety data will be analyzed descriptively by vaccine group. Safety laboratory data will be shown in a 3 x 3 table by visit using categorization of laboratory values of hematological and haematochemical blood tests and urinalysis according to institutional normal reference range (below, within, above).

7.4.3 Analysis of Key Secondary Immunogenicity Objectives

Not applicable.

7.4.4 Analysis of Key Secondary Efficacy Objectives

Not applicable.

7.4.5 Analysis of Key Secondary Other Objectives

Not applicable.

7.4.6 Analysis of Non-Key Objectives

Not applicable.

7.5 Planned Interim Analysis

For each cohort, after all subjects have completed enrolment and all vaccinations and post vaccination results (one month after dose 1, 2 and 3) are available, a group- unblinded preliminary immunogenicity analysis and a blinded interim safety analysis may be performed. Individual subject results from preliminary / interim analyses will not be made available to site and sponsor personnel until the end of the study.

8.0 SOURCE DOCUMENTATION, STUDY MONITORING, AND AUDITING

Study monitoring and auditing will be standardized and performed in accordance with the sponsor's standard operating procedures and applicable regulatory requirements (e.g., FDA, EMA, and ICH guidelines).

Prior to enrollment of the first study subject, NVGH or delegate will train investigators and/or their study staff on the study protocol, all applicable study procedures, documentation practices (including signing of the source data agreement (SDA, see section 8.1) and all electronic systems. CRFs supplied by the sponsor must be completed for each enrolled subject (see section 7.3.1 for definition of enrolled subject). Documentation of screened but not enrolled subjects must be maintained at the site and made available for review by the site monitor. All data entries as well as study related documents will be checked by the sponsor and/or site monitor. In addition, the investigator and site staff will be made aware of the plans to monitor the data collected at the site.

8.1 Source Documentation

Prior to the start of the study, the site staff participating in the study conduct will be trained on what documents will be required for review as source documentation (i.e., original records, laboratory reports, medical records, subject diaries. The kinds of documents that will serve as source documents will be specified in the Source Document Agreement (SDA) that will be available prior to first subject, first visit (FSFV).

In addition, source documentation **must** include all of the following: subject identification (on each page), eligibility and participation, proper informed consent procedures, dates of visits, adherence to protocol procedures, adequate reporting and follow-up of adverse events, documentation of prior/concomitant medication/vaccines, study vaccine receipt/dispensing/return records, study vaccine administration information, any data collected by a telephone conversation with the subject and date of completion and reason.

The subject must also allow access to the subject's medical records. Each subject must be informed of this prior to the start of the study and consent for access to medical records may be required in accordance with local regulations.

All safety data reported by subjects must be written down in source documents prior to entry of the data into CRFs. If there are multiple sources of information (e.g., Diary card, verbal report of the subject, telephone contact details, medical chart) supporting the diagnosis of an adverse event, these sources must be identified in the source documents, discrepancies between sources clarified, the ultimate diagnosis must be justified and written in the source documents, and this diagnosis must be captured in the adverse event CRF (AE CRF). The AE CRF must also capture which source(s) of information were used to determine the adverse event (e.g., subject recall, medical chart, Diary Card, and/or other sources).

8.2 Study Monitoring and Source Data Verification

A contract research organization (CRO) may be involved in the monitoring of protocol conduct and data entry. If a CRO is involved in study oversight, the name and address of this CRO will be located in the investigator site file. Prior to enrollment of the first study subject, NVGH will develop a Clinical Monitoring Plan, or equivalent documentation, to specify how monitoring will be performed for the study.

Study progress will be monitored by NVGH or its representative (e.g., a CRO) as frequently as necessary to ensure:

- that the rights and well-being of human subjects are protected
- the reported trial data are accurate, complete, and verifiable from the source documents and
- the conduct of the trial is in compliance with the current approved protocol/amendment(s), GC and applicable regulatory requirements

Contact details for the team involved in study monitoring will be identified in a handout located in the Investigator Site File. Study data recorded on CRFs will be verified by checking the CRF entries against source documents in order to ensure data completeness and accuracy as required by study protocol. Additional documents such as the investigator site file, pharmacy records, and informed consent documentation must also be available for review if requested. Arrangements for monitoring visits will be made in advance in accordance with the monitoring plan, except in case of emergency.

The investigator and/or site staff must make source documents of subjects enrolled in this study available for inspection by NVGH or its representative at the time of each monitoring visit. These documents must also be available for inspection, verification and copying, as required by regulations, by officials of the regulatory health authorities (e.g., FDA, EMA and others) and/or ECs/IRBs. The investigator and study site staff must comply with applicable privacy, data protection and medical confidentiality laws for use and disclosure of information related to the study and enrolled subjects.

9.0 DATA MANAGEMENT

9.1 Data Entry and Management

In this study, all data will be entered onto electronic case report forms (eCRFs) in a timely fashion by the investigator and/or the investigator's dedicated site staff. Data entered onto eCRFs are stored on a secure website. The data collected on this secure website are assimilated into EDC system, which is compliant with 21 Part 11 policies of the Code of Federal Regulations. The EDC will be designed and validated by NVx BCDM prior to activation for data entry by sites. The investigator must review data entered and electronically sign the eCRFs to verify their accuracy.

Access to the EDC system for data entry or review will require training and distinct individual access code assignments to those site staff members who will be entering study data and those involved in study oversight who may review study data. Data are collected within EDC, to which the sponsor and site monitors have exclusively "read only" access. eCRF data will be reviewed routinely by study personnel from NVx BCDM and clinical monitors.

If paper CRF (including pregnancy case report forms) is used for data collection, Three-part "no carbon required" (NCR) paper CRFs will be provided for each subject by the sponsor. All appropriate subject data collected during the study will be recorded on these forms. One copy must be retained by the investigator, and all other copies (including the original copy) will be returned as directed by the sponsor. Instructions on how to complete these forms will be provided to the investigator**.**

All study data must be entered by the investigator or delegate who will sign and date the CRFs. If the investigator delegates and authorizes other persons in his/her staff to make entries on the CRF, the names, positions, signatures and initials must be documented in writing (e.g., site delegation log).

CRFs must be completed during/after each study visit. Arrangements will be made by the study monitor to collect the CRFs upon completion. No CRFs are to be mailed to the sponsor without specific authorization.

Data from the CRFs are entered into the study database by NVx BCDM staff using single data entry. Verification is performed manually by a separate member of the BCDM staff by comparing the CRF to the data entered into the database.

All serology results produced by Clinical Serology, NVx will be entered into the Seroad database by NVx's Clinical Serology Laboratory, Marburg. All results will be checked in the laboratory for validity and completeness.

Electronic Data Transfer (EDT) is one method used by NVx for collecting laboratory data. The full-service laboratory (i.e., central laboratory) will send data as electronic files by a secured method (e.g., via diskette, CD, as an encrypted file attachment on electronic mail, or as a direct transfer into a specified server directory) to NVx's BCDM department. The data file is pre-processed and loaded by a member of the BCDM team into the study database. The laboratory will submit a results file containing the tests and the results as

specified in the protocol. If the laboratory provides the service, it will also submit a Demography (DEMOG) file containing the subject's demographic information. If the file includes results of data blinded to personnel in clinical research, the source will provide a separate results file that will be loaded into a separate laboratory table.

For this protocol, antibody laboratory data and safety laboratory data will be transmitted via EDT.

9.2 Data Clarification

As part of the conduct of the trial, NVGH may have questions about the data entered by the site, referred to as queries. The monitors and the sponsor are the only parties that can generate a query.

For eCRF trials, all corrections and clarifications will be entered into the EDC and will be identified by the person entering the information, the reason for the change, as well as the time of the changes made. If changes are made to a previously and electronically signed CRF, the investigator must confirm and endorse the changes.

9.3 Data Coding Procedures

Coding of Adverse Events, Medical History, and Prior and Concomitant Medications will be performed using standard dictionaries as described in the Data Management Plan.

9.4 Data Protection

NVGH respects the subjects' rights to privacy and will ensure the confidentiality of their medical information in accordance with all applicable laws and regulations.

The sponsor as Data Controller according to the European Directive on the protection of individuals with regard to the processing of personal data and on the free movement of such data [95/46/EC] confirms herewith compliance to Directive 95/46/EC in all stages of Data Management.

10.0 RECORD RETENTION

Investigators must retain all study records required by NVGH and by the applicable regulations in a secure and safe facility. The investigator must consult a NVGH representative before disposal of any study records, and must notify the sponsor of any change in the location, disposition, or custody of the study files. Essential documents must be retained until at least 2 years after the last approval of a marketing application in an ICH region and until there are no pending or contemplated marketing applications in an ICH region or at least 2 years have elapsed since the formal discontinuation of clinical development of the investigational product. “Essential documents” are defined as documents that individually and collectively permit evaluation of the conduct of a trial and the quality of the data produced. These documents should be retained for a longer period, however, if required by the applicable regulatory requirements or by an agreement with the sponsor. The Committee for Human Medicinal Products for Human Use (CHMP) requires retention for the maximum period of time permitted by the institution, but not less than 15 years (ICH E6, 4.9.5). It is the responsibility of the sponsor to inform the investigator/institution as to when these documents no longer need to be retained (ICH E6, 5.5.12).

These principles of record retention will also be applied to the storage of laboratory samples, provided that the integrity of the stored sample permits testing.

11.0 USE OF INFORMATION AND PUBLICATION

NVGH assures that the key design elements of this protocol will be posted in a publicly accessible database such as clinicaltrials.gov, and in compliance with current regulations.

NVGH also assures that key results of this clinical trial will be posted in a publicly accessible database within the required time-frame from the last subject's last study visit as dictated by applicable regulations.

Further to legislated data disclosure, NVGH will ensure that as far as possible results of this study will be published as scientific/clinical papers in high-quality peer-reviewed journals. Preparation of such manuscripts will be made with full collaboration of principal investigators and in accordance with the current guidelines of Good Publication Practice[16].

NVGH must be notified of any intent to publish data collected from the study and prior approval from NVGH must be obtained prior to publication.

12.0 ETHICS

12.1 Regulatory and Ethical Compliance

This clinical study was designed and shall be implemented and reported in accordance with the ICH Harmonized Tripartite Guidelines for Good Clinical Practice, with applicable local regulations including European Directive 2001/20/EC[13], Novartis codes on protection of human rights, and with the ethical principles laid down in the Declaration of Helsinki (European Council 2001, US Code of Federal Regulations, ICH 1997)[14]

At the end of the study, each subject will receive a monetary indemnification to cover for the time spent and any inconvenience due to the study participation.

12.2 Informed Consent Procedures

Eligible subjects may only be included in the study after providing written informed consent or assent, as described in section 3.2.1. Before the start of the trial, the investigator will have the informed consent and any other materials that will be provided to the subjects reviewed and approved by the IRB/EC. This review and approval will be documented and stored with other study documents. The investigator or designee must fully inform the subject or legal guardian of all pertinent aspects of the trial. A copy of the written informed consent will be given to the subject or the designee. The subject/designee must be allowed ample time to ask about the details of the trial and to make a decision as to whether or not to participate in the study. The subject and/or legal guardian must sign the consent form indicating their agreement to participate in the study before any study-related procedures are conducted. If the subject and/or legal guardian is unable to read and write, a witness must be present during the informed consent discussion and at the time of informed consent signature.

Prior to the start of the study, NVGH will provide to investigators a separate document with a proposed informed consent form that complies with the ICH GCP guideline and regulatory requirements and is considered appropriate for this study. Any changes to the proposed consent form suggested by the investigator must be agreed to by NVGH before submission to the IRB/EC and a copy of the approved version must be provided to the NVGH monitor after IRB/EC approval.

Women of child bearing potential should be informed that taking the study medication may involve unknown risks to the fetus if pregnancy were to occur during the study and agree that in order to participate in the study they must adhere to the contraception requirements indicated in the protocol for the duration of the study. If case of doubts on the ability of a subject to adhere to these requirements, that subject should not be allowed in the study.

12.3 Responsibilities of the Investigator and IRB/EC

The protocol and the proposed informed consent form must be reviewed and approved by a properly constituted IRB/EC before study start. Properly constituted IRB/EC is defined in ICH Guideline for Good Clinical Practice E6 (R1), Section 3 (ICH 1997). A signed and

dated statement that the protocol and informed consent have been approved by the IRB/EC must be given to NVGH before study initiation. Prior to study start and at any time the protocol is amended during study conduct, the investigator is required to sign a protocol signature page confirming his/her agreement to conduct the study in accordance with these documents and all of the instructions and procedures found in this protocol and to give access to all relevant data and records to NVGH monitors, auditors, Novartis Clinical Quality Assurance representatives, designated agents of NVGH, IRBs/ECs, and regulatory authorities as required. If an inspection of the clinical site is requested by a regulatory authority, the investigator must inform NVGH immediately that this request has been made.

The investigator also responsible for the following:

- maintaining a list of appropriately qualified persons to whom the investigator has delegated significant trial-related duties
- demonstrating the capability of recruiting the required number of suitable subjects within the recruitment period
- demonstrating sufficient time and staffing to properly conduct and complete the study within the agreed trial period
- ensuring that all persons assisting with the study are adequately informed about the protocol, the investigational product(s), and their study-related duties and functions
- ensuring that appropriately trained health care professionals are responsible for all study-related medical decisions and for ensuring appropriate medical care of subjects experiencing any adverse event related to the study
- if permission to do so is given by the subject, ensuring that the subject's primary healthcare provider is informed of the subject's participation in the study.

The investigator should not implement any deviation from, or changes of the protocol without agreement by the sponsor and prior review and documented approval/favourable opinion from the IRB/IEC of an amendment, except where necessary to eliminate an immediate hazard(s) to trial subjects, or when the change(s) involves only logistical or administrative aspects of the trial (e.g., change in monitor(s), change of telephone number(s)). In addition, the investigator, or person designated by the investigator, should document and explain any deviation from the approved protocol.

The investigator may implement a deviation from, or a change of, the protocol to eliminate an immediate hazard(s) to trial subjects without prior IRB/IEC approval/favourable opinion. As soon as possible, the implemented deviation or change, the reasons for it, and, if appropriate, the proposed protocol amendment(s) should be submitted:

(a) to the IRB/IEC for review and approval/favourable opinion,

(b) to the sponsor for agreement and, if required,

(c) to the regulatory authority(ies).

12.4 Protocol Adherence

Investigators will apply due diligence to avoid protocol deviations. Under no circumstances should the investigator contact NVGH or its agents, if any, monitoring the trial to request approval of a protocol deviation, as no authorized deviations are permitted. If the investigator feels a change to the protocol would improve the conduct of the study this must be considered a protocol amendment, and unless such an amendment is agreed upon by NVGH and approved by the IRB/EC it cannot be implemented. All significant protocol deviations will be recorded and reported in the Clinical Study Report.

12.5 Protocol Amendments

An amendment is a written description of change(s) to or formal clarification of a study protocol which may impact on the conduct of the clinical study, potential benefit of the clinical study, or may affect subject safety, including changes of study objectives, study design, subject population, sample sizes, study procedures, or significant administrative aspects. An administrative change of a study protocol is a minor correction or clarification that has no significant impact on the way the clinical study is to be conducted and no effect on subject safety (e.g., change of telephone number(s), logistical changes). Protocol amendments must be approved by NVGH, Health Authorities where required, and the IRB/EC. In cases when the amendment is required in order to protect the subject safety, the amendment can be implemented prior to IRB/EC approval. Notwithstanding the need for formal approval of a protocol amendment, the investigator is expected to take any immediate action required for the safety of any subject included in this study, even if this action represents a deviation from the protocol. In such cases, NVGH should be notified of this action, the IRB/EC at the study site, and, if required by local regulations, the relevant health authority) should be informed within 10 working days.

13.0 REFERENCE LIST

[1] Bardhan P, Faruque AS, Naheed A, Sack DA (2010) Decrease in shigellosis-related deaths without Shigella spp.-specific interventions, Asia. Emerg Infect Dis 16: 1718-1723

[2] Kotloff KL, Winickoff JP, Ivanoff B, Clemens JD, Swerdlow DL, Sansonetti PJ, Adak GK, Levine MM (1999) Global burden of Shigella infections: implications for vaccine development and implementation of control strategies. Bull World Health Organ 77: 651-666.

[3] Zhang W, Luo Y, Li J, Lin L, Ma Y, Hu C, Jin S, Ran L, Cui S (2011) Wide dissemination of multidrug-resistant Shigella isolates in China. J AntimicrobChemother 66: 2527-2535.

[4] Levine MM, Kotloff KL, Barry EM, Pasetti MF, Sztein MB (2007) Clinical trials of Shigella vaccines: two steps forward and one step back on a long, hard road. Nat Rev Microbiol 5: 540-553

[5] Cohen D, Ashkenazi S, Green MS, Gdalevich M, Robin G, Slepon R, Yavzori M, Orr N, Block C, Ashkenazi I, Shemer J, Taylor DN, Hale TL, Sadoff JC, Pavliakova D, Schneerson R, Robbins JB (1997) Double-blind vaccine-controlled randomised efficacy trial of an investigational Shigella sonnei conjugate vaccine in young adults. Lancet 349: 155-159

[6] Passwell JH, Ashkenazi S, Harlev E, Miron D, Ramon R, Farzam N, Lerner-Geva L, Levi Y, Chu C, Shiloach J, Robbins JB, Schneerson R (2003) Safety and immunogenicity of Shigella sonnei-CRM9 and Shigella flexneri type 2a-rEPAsucc conjugate vaccines in one- to four-year-old children. Pediatr Infect Dis J 22: 701-706

[7] Passwell JH, Ashkenzi S, Banet-Levi Y, Ramon-Saraf R, Farzam N, Lerner-Geva L, Even-Nir H, Yerushalmi B, Chu C, Shiloach J, Robbins JB, Schneerson R (2010) Age-related efficacy of Shigella O-specific polysaccharide conjugates in 1-4-year-old Israeli children. Vaccine 28: 2231-2235.

[8] Beveridge TJ (1999) Structures of gram-negative cell walls and their derived membrane vesicles. J Bacteriol 181: 4725-4733

[9] Berlanda SF, Colucci AM, Maggiore L, Sanzone S, Rossi O, Ferlenghi I, Pesce I, Caboni M, Norais N, Di C, V, Saul A, Gerke C (2012) High yield production process for Shigella outer membrane particles. PLoS One 7: e35616. 10.1371

[10] EMEA/CHMP/VWP/164653/2005

[11] CHMP Note for Guidance on preclinical, pharmacological and toxicological testing of vaccines (CPMP/SWP/465/95).

[12] Gaston JS, Inman RD, Ryan ET, Venkatesan MM, Barry EM, Hale TL, Bourgeois AL, Walker RI (2009) Vaccination of children in low-resource countries against Shigella is unlikely to present an undue risk of reactive arthritis.Vaccine 27, 5432-5434

[13] European Parliament (2001): Directive 2001/20/EC of the European Parliament and of the Council of 4 April 2001. Official Journal of the European Communities. L 121/34-44

[14] 59thWorld Medical Association General Assembly (October 2008) Declaration of Helsinki - Ethical Principles for Medical Research Involving Human Subjects. Seoul, Korea

[15] Graf C, Battisti WP, Bridges D (2009). Good publication practice for communicating company sponsored medical research: the GPP2 guidelines. BMJ; 339: b4330

[16] Guidance for Industry: Toxicity Grading Scale for Healthy Adult and Adolescent Volunteers Enrolled in Preventive Vaccine Clinical Trials. U.S. Department of Health and Human Services, Food and Drug Administration, Center for Biologics Evaluation and Research - September 2007
