# Supplementary material for: Neutropenia as an Adverse Event following Vaccination: Results from Randomized Clinical Trials in Healthy Adults and Systematic Review
Source: PLoS One. 2016 Aug 4;11(8):e0157385. doi: 10.1371/journal.pone.0157385 (PMC4974007; doi:10.1371/journal.pone.0157385)
Supplement: S5 Protocol — (PDF) [file pone.0157385.s010.pdf]

A clinical study to generate a set of data characterising clinical events, physiological responses, and innate and adaptive immune responses following a single intramuscular immunisation with Fluad™ seasonal influenza vaccine or saline as placebo control in healthy adults.

EudraCT Number: **2014-003543-35**

Version Number: Version 3, Final

Date: **10 SEP 2014**

Confidentiality Statement

The information contained in this document is the property of the Centre for Vaccinology (CEVAC), Ghent University and Ghent University Hospital and is provided to you in confidence as an investigator, potential investigator, sponsor, or consultant, for review by you, your staff and an applicable Ethics Committee (EC). It is understood that this information will not be disclosed to others without written authorisation from CEVAC (UGent/UZGent), except to the extent necessary to obtain written informed consent from those persons to whom the drug may be administered.

CLINICAL STUDY PROTOCOL AGREEMENT FORM

Authorisation of final version

A clinical study to generate a set of data characterising clinical events, physiological responses, and innate and adaptive immune responses following a single intramuscular immunisation with Fluad^{TM} seasonal influenza vaccine or saline as placebo control in healthy adults.

On behalf of the Sponsor: Ghent University Hospital – Center for Vaccinology

CEO UZGent

Signature ……………………………. Date ……………………….

Name: Prof. Dr. E. MORTIER

Principal Investigator Agreement

I agree to conduct this study according to this protocol and to comply with its requirements, subject to ethical principles that have their origins in the Declaration of Helsinki, safety considerations and the applicable laws and regulations, I agree to conduct the study in accordance with local regulations and the International Conference on Harmonisation Topic E6: Guideline for Good Clinical Practice (ICH GCP).

I will promptly submit the protocol to applicable Ethical Review Board. I agree not to make any changes to the protocol without agreement from the sponsor and prior review and written approval from the local Ethical Review Board, except where necessary to halt an immediate threat to subject safety, or for administrative study details when such actions are permitted by local regulations.

I understand fully the appropriate use of Fluad^{TM} as described in this protocol, and any other relevant material made available by the sponsor.

I will make certain that all personnel assisting with the study will be adequately informed about the study medication and their study-related duties as described in the protocol.

I understand that, should the decision be made by the Sponsor to terminate prematurely or suspend the study, at any time and for whatever reason, such decision will be communicated to me in writing. Conversely, should I decide to withdraw from execution of the study, I will communicate immediately in writing to the Sponsor or their representatives.

Principal Investigator – Director CEVAC

Signature ……………………………. Date ……………………….

Name: Prof. Dr. Geert LEROUX-ROELS

TABLE OF CONTENTS

1 CLINICAL STUDY PROTOCOL AGREEMENT FORM 2
2 TABLE OF CONTENTS 3
3 LIST OF ABBREVIATIONS AND DEFINITIONS 5
4 CONTACT INFORMATION 6
5 PROTOCOL SYNOPSIS 8
6 STUDY FLOW CHARTS 12
6.1 Study Overview Flow Charts 12
7 SUMMARY OF STUDY DESIGN AND RATIONALE 13
7.1 Introduction and Study Rationale 13
7.2 Summary of Study Design 17
7.3 Profile of Study Drugs 17
7.4 Potential Risks and Benefits 17
7.5 Rationale For Study Design 18
7.5.1 Rationale for Doses 18
7.5.2 Rationale for Exploratory Study Endpoints 18
7.5.3 Blinding 18
8 STUDY OBJECTIVES AND ENDPOINTS 19
8.1 Exploratory Study Objectives 19
8.2 Study Endpoints (study variables measured) 19
9 SUBJECT SELECTION AND WITHDRAWAL CRITERIA 20
9.1 Population base 20
9.2 Inclusion criteria 20
9.3 Exclusion criteria 21
9.4 Discontinuation and Withdrawal of subjects from the study 22
10 CLINICAL STUDY PROTOCOL 22
10.1 Study Procedures 23
10.2 Duration of Study Participation 23
10.3 Study Restrictions: prohibited vaccination and medication 23
11 STUDY MEDICATION 23
11.1 Treatment Plan 23
11.2 Preparation, Administration and Dosage of Study Medication 24
11.2.1 Preparation Instructions 24
11.2.2 Route of administration 24
11.2.3 Dose and Dosing Schedule 24
11.3 Packaging and Labelling 24
11.4 Storage and Accountability of Drug Supplies 24
11.5 Destruction of Clinical Supplies 24
11.6 Responsibilities 24
11.7 Compliance 25
11.8 Treatment Assignment Procedures 25
11.8.1 Randomisation 25
11.8.2 Blinding/Unblinding 25
12 STUDY DATA PARAMETERS 25
12.1 Laboratory Assessments 25
12.1.1 Study Blood Volume 26

12.1.2 Urinalysis 27
12.1.3 Urine pregnancy test 27
12.2 Vital signs 27
12.3 Measurement of temperature and injection site reactions 27
12.4 Methods and Timing for Assessing and Recording Study Data Parameters27
13 SAFETY MONITORING 28
13.1 Specification of Safety Parameters 28
13.1.1 Laboratory and Other Safety Assessment Abnormalities Reported as AEs and SAEs 28
13.1.2 Definition of an AE 28
13.1.3 Definition of a SAE 29
13.2 Evaluating and Recording (Serious) Adverse Events 29
13.3 Intensity of Event 30
13.4 Relationship to study product 31
13.5 Expectedness of SAEs 32
13.6 Reporting of serious adverse events, pregnancies, and other events 32
14 STATISTICAL CONSIDERATIONS AND ANALYTICAL PLAN 33
14.1 Responsibility for Analysis 33
14.2 Justification of Sample Size 33
14.3 Definition of Study Completion 34
14.4 Definition of Criteria for Termination of the Study 34
14.5 Analysis of Clinical Events and Exploratory Endpoints after immunisation34
15 STUDY DOCUMENTATION ADMINISTRATION 34
15.1 Source documents and eCRF 34
15.2 Monitoring 35
15.3 Access to Source Data Documents 35
15.4 Data Handling and Record Retention 35
15.5 Subject Confidentiality and Data Protection 35
16 QUALITY CONTROL AND QUALITY ASSURANCE 36
17 CLINICAL STUDY PROTOCOL DEVIATIONS AND AMENDMENTS 36
18 CONDITIONS FOR TERMINATING THE STUDY 37
19 ETHICAL AND REGULATORY REQUIREMENTS 37
19.1 Informed Consent 37
19.2 Independent Ethics Committee (IEC) 37
19.3 Final Reports 38
19.4 Competent Authority 38
20 FINANCE AND INSURANCE 38
21 PUBLICATIONS 38
22 REFERENCES 38
23 Laboratory Safety Analysis. 39

LIST OF ABBREVIATIONS AND DEFINITIONS

AE	Adverse Event
Bimetra	Clinical Research Center Ghent
BMI	Body Mass Index
BP	Blood Pressure
CEVAC	Centre for Vaccinology Ghent
CMI	Cell Mediated Immunity
CRF	Case Report Form
EC	Ethics Committee
ESR	Erythrocyte Sedimentation Rate
FBC	Full Blood Count
GCP	Good Clinical Practice
HAI	Haemagglutination Inhibition
ICH	International Conference on Harmonisation
IEC	Independent Ethics Committee
IM	Intramuscular
PBMC	Peripheral Blood Mononuclear Cells
PI	Principal Investigator
SAE	Serious Adverse Event
SC	Subcutaneous
SmPC	Summary of Product Characteristics
SOPs	Standard Operating Procedures
Surrey CRC	Surrey Clinical Research Centre

CONTACT INFORMATION

PRINCIPAL INVESTIGATOR	Name: Geert Leroux-Roels, MD, PhD<br>Address: CEVAC, De Pintelaan 185, 9000 Gent, Belgium<br>Tel: ++32 9 332 21 23<br>Fax: ++32 9 332 22 85<br>Email: Geert.LerouxRoels@ugent.be
Principal Clinical Research Physicians	Name: Cathy Maes, MD<br>Address: CEVAC, De Pintelaan 185, 9000 Gent, Belgium<br>Tel: ++32 9 332 94 65<br>Fax: ++32 9 332 22 85<br>Email: Cathy.Maes@uzgent.be<br><br>Name: Annelies Aerssens, MD<br>Address: CEVAC, De Pintelaan 185, 9000 Gent, Belgium<br>Tel: ++32(0)93320072<br>Fax: ++32(0)93322285<br>Email: Annelies.Aerssens@uzgent.be
Clinical Research Coordinator	Name: Fien De Boever, MSc<br>Address: CEVAC, De Pintelaan 185, 9000 Gent, Belgium<br>Tel: ++32 9 332 34 93<br>Fax: ++32 9 332 22 85<br>Email: Fien.DeBoever@uzgent.be
COLLABORATING LABORATORIES	Laboratorium voor klinische biologie<br>Universitair Ziekenhuis Gent<br>De Pintelaan 185<br>9000 Gent – Belgium<br>Contact: Prof. G. Leroux-Roels<br>Tel: +32 9 332 21 23<br>Fax: +32 9 332 63 11<br>Email: Geert.LerouxRoels@ugent.be<br><br>CEVAC Core Lab<br>Universitair Ziekenhuis Gent<br>De Pintelaan 185<br>9000 Gent – Belgium<br>Contact: Frederic Clement<br>Tel: +32 9 332 36 54<br>Fax: +32 9 332 63 11<br>Email: Frederic.Clement@ugent.be

Max Planck Institute for
Infection Biology (MPIIB)
Department for Immunology
Chariteplatz 1
10117 Berlin
Germany
Contact person: Dr Gayle McEwen
Email: mcewen@mpiib-berlin.mpg.de

deCODE genetics
Sturlugata 8, Reykjavik
IS-101 Iceland
Contact: Ingileif Jónsdóttir
Email: Ingileif.Jonsdottir@decode.is

Novartis Vaccines and Diagnostics
Via Fiorentina 1
53100 Siena, Italy
Contact: Giuseppe Del Giudice, MD, PhD
Global Head Translational Medicine
Tel. +39 0577 243261 (direct) or 3050 (assistant)
Fax +39 0577 243564
Email giuseppe.del_giudice@novartis.com

VisMederi srl
Via Fiorentina 1
53100 SIENA-ITALY
Contact: Prof E. Montomoli
Tel: +39-0577231253
Fax: +39-057743444
Email: emanuele.montomoli@unisi.it
vismederi@pec.it

Other laboratories at Biovacsafe collaborators may be used for the analysis of other laboratory parameters.

PROTOCOL SYNOPSIS

**Title**	**A clinical study to generate a set of data characterising clinical events, physiological responses, and innate and adaptive immune responses following a single intramuscular immunisation with Fluad™ seasonal influenza vaccine or saline as placebo control in healthy adults.**
**Sponsor**	CEVAC – Ghent University and Ghent University Hospital
**Principal Investigator**<br>**Study Location**	Geert Leroux-Roels, MD, PhD<br>CEVAC - Ghent University and Ghent University Hospital
**Indication**	Biomarkers of vaccine safety and immunogenicity
**Study Objectives**	To generate data to undergo integrated systems biology analysis to validate biomarkers identified in the exploratory studies conducted previously or to identify new biomarkers of responses to immunisation<br><br>The data set will include data characterising:<br>1. Physiological responses at various time points after immunisation by measuring:<br>a. Local and systemic vaccine-related clinical events.<br>b. Physiological assessments: heart rate, temperature, blood pressure.<br>c. Haematology (blood counts and ESR) and biochemistry parameters.<br>2. Innate and adaptive immune responses including:<br>a. Innate immune activation detected by global gene expression in whole blood<br>b. Adaptive humoral immunity determined by serum HAI titre<br>c. Innate and adaptive immune activation detected by gene pathway activation in whole blood<br>d. Metabolic responses as detected by metabolic gene expression and pathway activation in whole blood<br>e. Immune activation detected by:<br>i. Concentration of selected inflammatory soluble mediators in serum including:<br>1. chemokines and cytokines<br>2. acute phase proteins<br>ii. PBMC cytokine secretion, proliferation or surface markers in response to *in vitro*

	antigen stimulation.<br>f. As an exploratory endpoint, the adaptive cellular immune response will be evaluated via counting HA-specific $CD4^+$ T cells expressing activation markers and/or cytokines following in vitro stimulation and analysis by flow cytometry.<br>3. Genetic testing of subjects when deemed necessary (genetic testing analysis may be SNP analysis or full genome analysis).<br>4. Correlations in changes in innate immune activation and metabolism with adverse events, haematology and biochemistry panels, genotype and physiological assessments
**Study Design**	Observer-blind (subject, investigator and laboratory blinded), randomised, placebo controlled exploratory "confirmatory study".
**Population**	Healthy adults
**Main Selection Criteria**	• Age: 18 – 45 years<br>• Male: Female ratio: no more than 2/3 of the population should be of either gender<br>• Healthy: no active disease process that could interfere with endpoints measured as determined by medical history<br>• Not taking regular medications that could interfere with endpoints measured<br>• No contraindications to Fluad™ vaccine
**Study Medicinal Products:**<br>Formulations<br>Route of Administration<br>Dose regimen	GROUP A<br>• Fluad™, seasonal trivalent inactivated influenza vaccine for season 2014-2015 (Northern hemisphere)<br>• Single 0.5 mL dose<br>• Intramuscular<br>• One injection on one occasion<br>• 228 subjects
**Concurrent Controls**	• GROUP B<br>• Saline placebo 0.5 mL<br>• Intramuscular<br>• One injection on one occasion<br>• 12 subjects
**Exploratory Study Endpoints (study variables measured)**	1. Frequency of local and systemic vaccine-related clinical events at all time points from vaccination up to last study visit.<br>2. Change from pre-immunisation baseline values in pulse, temperature, blood pressure at all time points from time

	of immunisation up to last study visit.<br>3. Change from pre-immunisation baseline values in haematology (blood counts and ESR), biochemistry (liver, renal and bone panels) parameters at selected time points from time of immunisation up to last study visit.<br>4. Change from pre-immunisation baseline values in global gene expression measured on whole blood samples at selected time points from time of immunisation up to last study visit<br>5. Change from pre-immunisation baseline values and fold increase in serum HAI titre in serum samples at selected time points from time of immunisation up to last study visit.<br>6. Change from pre-immunisation values of adaptive cellular immune response will be evaluated at Day 7 in all subjects via enumeration of HA-specific $CD4^+$ T cells expressing activation markers and/or cytokines following in vitro stimulation and analysis by flow cytometry.<br>7. Change from pre-immunisation baseline values in metabolic gene expression and pathway activation measured on whole blood samples at selected time points from time of immunisation up to last study visit<br>8. Change from pre-immunisation baseline values in concentration of selected cytokines and acute phase proteins in serum samples at selected time points from time of immunisation up to last study visit<br>9. Change from pre-immunisation baseline values in PBMC cytokine secretion in response to *in vitro* antigen stimulation at selected time points from time of immunisation up to last study visit<br>10. Genetic analysis of subject<br>11. Paxgene™ tubes will be drawn at all time points in order to be enable the evaluation of gene expression changes at other time points than those selected for item 4 (if deemed necessary).
**Assessment Schedule**	**Screening and Immunisation Visit (Day 0)**<br>• Informed Consent<br>• Demography<br>• Medical history and concomitant medication<br>• Vital signs (blood pressure, heart rate, oral temperature)<br>• Assessment of inclusion/exclusion criteria<br>• Height, weight, BMI<br>• Physical exam

	• Urine pregnancy test<br>• Randomisation<br>• Clinical samples as per Table 2<br>• Blood sample for DNA genotype<br>• Blood sample for CMI testing<br>• Vaccine administration<br>• (S)AEs recorded<br>• Vital signs (blood pressure, heart rate, oral temperature)<br><br>**Follow Up Visits (for the subgroup with 5 visits: Day 1, Day 3, Day 7 and Day 21 and for the subgroup with 3 visits: Day 1 and Day 7)**<br>• Vital signs (blood pressure, heart rate, temperature)<br>• Continued eligibility check<br>• Concomitant medications<br>• Clinical samples as per Table 2<br>• Blood sample for CMI testing on day 7 (Visit 4) only<br>• (S)AEs recorded<br><br>**Phone Call Day 21 (for the subgroup with 3 visits):**<br>• Concomitant medications<br>• (S)AEs recorded
**Data Analysis**	Exploratory endpoints will be analysed by a Systems Biology approach to identify exploratory biomarkers, the statistical analysis plan will be developed by the BioVacSafe consortium.
**Duration of Study Period (per subject)**	21 days

STUDY FLOW CHARTS

Study Overview Flow Charts

Table 1: Study Visits Overview

Visit Name	Visit 1<br>Day 0	Visit 2<br>Day 1	Visit 3<br>Day 3	Visit 4<br>Day 7	Visit 5<br>Day 21	Phone Call<br>Day 21
**Group A (Fluad)**						
• Subgroup with 3 visits (n = 114)	X	X		X		X
• Subgroup with 5 visits (n = 114)	X	X	X	X	X	
**Group B (Placebo)**						
• Subgroup with 3 visits (n = 6)	X	X		X		X
• Subgroup with 5 visits (n = 6)	X	X	X	X	X	

Table 2: Study Procedures Flow Chart

Visit Name	Visit 1<br>Day 0	Visit 2<br>Day 1	Visit 3<br>Day 3	Visit 4<br>Day 7	Visit 5<br>Day 21	Phone Call<br>Day 21
**Procedures**						
Written informed consent	X					
Demographic data	X					
Physical exam	X					
Medical history	X					
Concomitant medication	X	X	X	X	X	X
Vital signs [a]	X	X	X	X	X	
Laboratory safety [b]	X	X	X	X	X	
Screening serology (hepatitis B and C, HIV)	X					
Pregnancy Test: Urine [c]	X					
Vaccine administration	X					
Serum immunology [e]	X	X	X	X	X	
Whole blood for PBMC isolation [d,e]	X			X		
Paxgene tube	X	X	X	X	X	
Recording of (serious) adverse events after immunisation	X	X	X	X	X	X

[a] Heart rate, blood pressure, oral temperature
[b] Laboratory safety as detailed in section 23
[c] Female subjects of childbearing potential only
[d] On day 0 and day 7, the subgroup with 3 visits will donate 120 ml of blood (90 ml for PBMC + 30 ml for cell fractionation) whereas subgroup with 5 visits will donate 90 ml of blood (for PBMC preparation only)
[e] Serum and Isolated PBMC will be bio-banked and made available for further read-outs.

SUMMARY OF STUDY DESIGN AND RATIONALE

Introduction and Study Rationale

Currently licensed vaccines are widely accepted to be safe and to have an acceptable reactogenicity profile. Vaccine development lead-times are extremely long and expensive due to the requirements for the extensive safety and efficacy testing required prior to Market Authorization. Rarely, significant adverse reactions have been detected post-licensure that were not detected during the development of the vaccine and only became apparent during large-scale Phase IV post marketing surveillance (e.g. intussusception, Bell's palsy). Such events have led to the withdrawal of vaccines from the market, resulting in financial impact, loss of confidence in vaccines, and individual harm to those affected and potentially to those exposed to vaccine-preventable diseases by decreases in immunization rates.
Clinical trials during the early stage pre-marketing assessment of safety and efficacy are not designed or powered to study immunopathological responses intensively enough to detect *transient* or *infrequent* phenomena (e.g. auto-reactive B and T cells, inflammation), or in sufficient *depth* (frequency of sampling, application of advanced technology) to be able to predict rare or subtle events which, in susceptible recipients, may subsequently trigger or lead to exacerbation of autoimmune or inflammatory diseases.

Vaccines are thought to trigger innate inflammatory responses to induce antigen-specific adaptive immunity (the desired effect of a vaccine), but excessive inflammation may lead to serious inflammatory complications or unwanted side effects. A lack of reliable biomarkers predicting severe inflammation has halted several exploratory vaccines, and withdrawn some licensed vaccines, some of which were associated with inflammatory complications, albeit low frequency, in some individuals. The BIOVACSAFE project, a 5-year €30M project funded by the Innovative Medicine Initiative, will undertake a **series of correlated clinical studies** that will apply and develop technologies to generate *clinical* data on inflammation with licensed vaccines as benchmarks, and identify biomarkers to predict acceptable reactogenicity, for correlation with standardized clinical readouts and inflammatory markers assessed in natural infections.

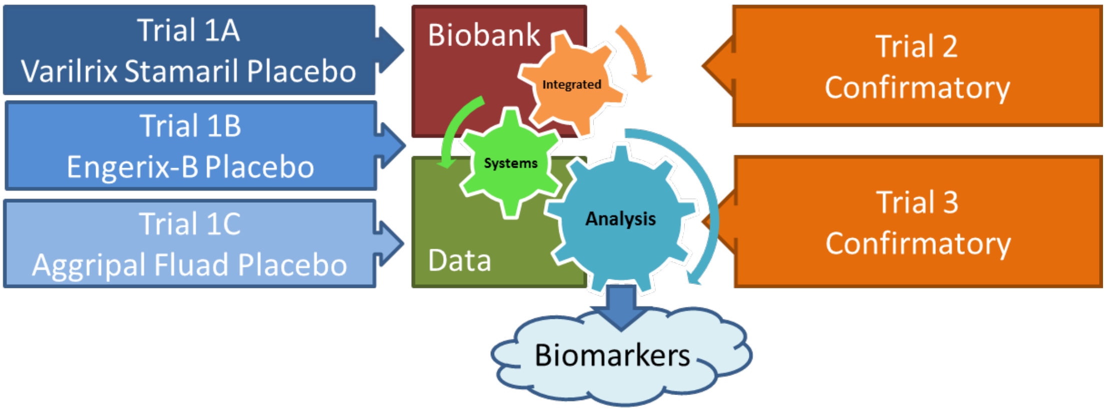

The overall project will follow the general structure of three studies employing five different vaccines plus placebo groups that will generate a biobank of samples and a set of data (clinical responses, gene responses, proteins and immunology responses).

Apart from using different vaccines, each study will follow the same protocol to allow the data to be combined into one dataset and biobank. Integrated systems biology analysis of the data and iterative access to the biobank, will identify putative "biomarkers" of inflammation.

Following these studies, two larger clinical trials will be conducted in CEVAC (Ghent University and Ghent University Hospital) to confirm or refute the validity of these putative biomarkers. These "confirmatory trials" will also add to the database and biobank to allow further rounds of integrated systems biology analysis to generate hypotheses and putative biomarkers for future research projects. The scheme is illustrated in the diagram above. The study described herein is the first of these larger clinical trials.

The combination of studying basic responses at the protein, gene and metabolic level is often termed "biomics". Biomics can be exploited to discover ***biomarkers*** (a measurable biological response that predicts something), which may be useful for monitoring of vaccine trials, and ideally can predict occurrence of beneficial and detrimental effects that are directly correlated with side effects and efficacy of vaccines undergoing clinical testing in humans. The different biomics include transcriptomics (genes), metabolomics (lipids and metabolites) and proteomics (proteins). In this project transcriptomics and metabolomics, together with profiling of cytokines and chemokines (molecules produced in immune responses) will be harnessed for assessment of vaccine responses with an emphasis on immunosafety and immunogenicity. Global gene expression profiling by transcriptomics has led to the definition of biosignatures, which can be used to discriminate diseased from healthy individuals. More recently, such studies have also been exploited for monitoring of vaccine effects in vaccinees (Pulendran et al., 2010).

Blood transcriptional profiles reflect the immune status of the host and expression patterns change upon infection and early inflammation. Cytokine profiles are linked to the blood transcriptome because they are, in part, influenced by leukocyte gene expression, but contain additional information resulting from processes occurring in extra-vascular tissues. Metabolomics identifies and quantifies small molecules to provide insights into changes in general metabolomic processes, while multiplex cytokine/chemokine profiling provides a "batch immune answer" of inflammatory processes. Both of these platforms complement transcriptomics and are known to be good predictors for disease and/or inflammation, underlining their relevance to vaccine safety. The technologies used in this clinical study are integrated with other technologies to deliver state of the art biomics and systems biology analysis for an integrated set of Clinical Studies (parallel studies using the same vaccines in animal models), and related population-based studies of infections and allergy. The clinical studies of which this is one will closely integrate with other activities within the BIOVACSAFE project to translate biomarker discovery into practical tools for vaccine development and regulation.

The characterization of early innate immune system events following immunization (days 0-3, 7-30) by gene expression and multiplex cytokine/cellular response analysis (systems biology approach) have been used to successfully identify biomarkers of inflammation and immune response/efficacy. This has been done in a predictive and reproducible way by immunizing relatively small numbers of healthy subjects, for example with live viral Yellow Fever vaccine (Querec et al., 2009) or adjuvanted

subunit malaria vaccines (Vahey et al., 2010). This opens-up the novel possibility to conduct intensive and highly focused, but relatively small clinical trials early-on in the development of novel vaccine technologies to identify biomarkers that may be predictive of safety signals that may only become apparent in subsequent larger scale clinical testing or during post marketing surveillance.

We propose to apply a systems biology approach to identify predictive biomarkers of vaccine immunosafety in the context of an initial "Training Study" that incorporate intensive clinical monitoring of vaccine recipients, standardized Adverse Event definitions, accepted measurements of immune responses and in which the analytic plan will strive to correlate biomarker activity with observed physiological and immunological responses to vaccination. In addition, while studies of transcriptomics or other biomics technologies have been used in previous studies, we will for the first time bring together metabolomics, transcriptomics and other biomics technologies, together with readouts of immune-efficacy and standardized definitions of adverse reactions, to the simultaneous evaluation relevant licensed vaccines studied under a highly standardized clinical setting to assess the relationships between these biomarkers and the short term reactogenicity of, and immune responses to, the selected vaccines. We will, in particular, use detailed metabolic models to link transcription data with metabolomics data and hereby potentially improve the statistical power in terms of biomarker identification. If successful, these biomarkers could be used in early stage clinical trials to optimize selection of vaccine candidates with a profile that will be unlikely to generate worrisome safety signals once they are in generalized use.

Following this intense series of studies in small numbers of subjects a very large dataset of clinical, and biological data will be generated together with a biobank of samples. Integrated systems biology analysis will then be used where the biobank can be accessed to study relevant time points, a model of responses built with biomarkers associated with clinical events identified, and then the biobank re-accessed to further extend the analysis in an iterative way. Based on the preliminary findings we will then design the second and third large outpatient clinical trials to confirm the results of the systems biology analysis. This is illustrated in the figure:

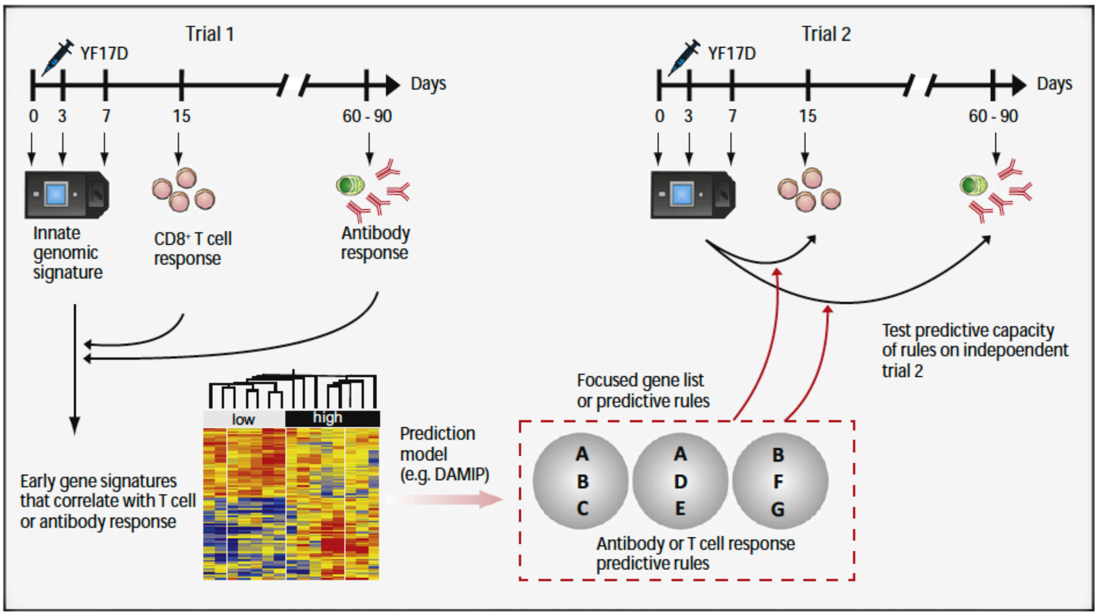

An important feature of the BIOVACSAFE project is the fact that we will restrict to the study of licensed vaccines which are accepted by regulatory authorities as being safe, well tolerated, and sufficiently efficacious to warrant their recommendation for general use. We do not have access to vaccines that have been shown in pre- or post-marketing studies to be unsafe or non-efficacious. Thus, the conclusions that can be drawn from these studies must be understood in the context of the null hypothesis that vaccines having the biomarker profiles identified in these studies do not present a potential safety risk for the general population. This does not mean that in certain genetically predisposed individuals, or in persons with active underlying autoimmune disease, these vaccines could potentially trigger or exacerbate autoimmune disease.

In the past year (2013-2014) intense training studies have been conducted, involving groups of 20 subjects randomized per vaccine treatment group, with matched male: female ratio. Subjects in the placebo groups of each of these studies have been pooled to give a placebo group size of at least 20. Consideration will be given in the analysis to the different routes of immunisation (SC and IM). In addition, each subject acts as their own control for kinetics with comparison from the baseline pre-immunisation levels of biomarkers measured.

Due to the large numbers of individual samples generated across the proposed time course of monitoring, and restrictions on budget, we will biobank all samples at all time points measured. Subsequently, in collaboration with parallel studies in animal models and based on the observed clinical signs and symptoms for each vaccine, analysis of selected time points will be undertaken for all subjects to identify biomarkers. In a subset of subjects the full time course will be analysed to generate kinetics, based on the reactogenicity observed. All selection processes will involve the External Advisory Board of the BIOVACSAFE project who are international experts in the field of Systems Biology and vaccine safety. Where certain post immunization events are recorded (e.g. fever, injection site reaction) a full time course analysis across the event will be analysed within those subjects, acting as their own controls. Biomarkers that are significant according to previously published criteria for gene expression (e.g. Pulendran et al., 2010) will be selected for advancing into the animal models WP2, and into later stage clinical confirmatory trials.

Clinical observations (made during the 7-day stay in the ward and at the subsequent outpatient clinic visits), haematology and chemistry data obtained on numerous points in time as well as gene-expression and cytokine/chemokine analyses on selected moments, have meanwhile been analysed and are guiding the selection of the study vaccines as well as the design of two planned **Confirmatory Phase IV clinical trials of which this trial is the first one.**

If successful, the biomarkers discovered during the exploratory and confirmatory trials can be used in early stage clinical trials to optimize selection of vaccine candidates with a profile that will be unlikely to generate worrisome safety signals once they are in generalized use.

Summary of Study Design

Based on the data derived from the exploratory studies, this first confirmatory study will use Fluad^{TM} as the study vaccine. Fluad^{TM} is an **adjuvanted subunit** influenza vaccine, and it will be given to a population of healthy adults, aged 18 to 45 years, that has been **previously primed** to influenza antigens (haemagglutinin (H) and neuraminidase (N)) either through prior vaccination and/or exposure to natural infection.

The study is single centre, observer-blinded, placebo-controlled and randomised.

Subjects will receive a single dose of the vaccine at the recommended dose level according to the SmPC. The following will be measured - clinical events (recorded adverse events), physiological responses (heart rate, blood pressure, temperature, injection site), metabolic responses via metabolic gene expression in whole blood, innate immune responses (cytokine levels and whole blood gene expression) and adaptive immune responses (serum antibody and antigen-specific cellular responses) at various time points after immunisation.

At each study visit, full physiological parameters (including temperature, heart rate, BP) will be obtained and the injection site will be examined with any redness or swelling measured and recorded. Standardized diary cards will be used to collect solicited and unsolicited clinical event data at each blood draw time point, the diary cards will be examined at each visit and any relevant clinical events will be entered into the relevant clinical event form. Participants will be asked to monitor oral temperature from day 0 until day 7 at the following time points: 8:00 am; noon: 4:00 pm and at 10:00 pm (or when going to bed). The results of these measurements will be monitored in a diary card. Any skin reactions at the site of injection will be evaluated; largest diameter of redness and swelling will be measured with a ruler and data reported on the diary card. Samples of blood (plasma, serum and PBMCs) will be collected for analysis and processing using protocols already in place. Subjects will also have blood obtained for standard pathology markers (haematology, biochemistry) as well as acute phase proteins.

In this study we will seek to corroborate the biomarkers associated with reactogenicity, or lack of reactogenicity that have been defined in the "training" studies or identify new ones that were not identified in these previous studies.

Profile of Study Drugs

FLUAD^{TM} – MF59C.1-adjuvanted, subunit influenza vaccine as produced and distributed for the 2014/2015 Northern Hemisphere influenza season.

Potential Risks and Benefits

Please refer to the SmPCs. The Fluad^{TM} vaccine will be administered as an intramuscular injection of 0.5 mL in the upper arm (deltoid region of the non-dominant arm) according to the SmPCs with the following exceptions:
Fluad^{TM} will be administered here to a population of 18 – 45 year olds, not the indicated population of 65 years and older.

FLUAD™

The SmPC details that FLUAD™ is a trivalent, surface antigen, inactivated influenza virus vaccine, adjuvanted with MF59C.1. It is indicated for active immunization against influenza in the elderly (≥ 65 years). Fluad™ is contraindicated in individuals with a known hypersensitivity to the active components, any of the excipients, eggs, chicken proteins, kanamycin and neomycin sulphate, formaldehyde, and cetyltrimetholammonium bromide or those who have had a previous life-threatening reaction to previous influenza vaccinations. The most common local adverse drug reactions are pain and temperature at injection site and erythema.

Rationale For Study Design

Rationale for Doses

The study will use the dose of Fluad indicated in the SmPC as the purpose of this study is to characterise clinical events, physiological and metabolic responses, and innate and adaptive immune responses following immunisation with products used in accordance with the SmPC. The placebo will be the same volume of physiological saline administered by the same route.

Rationale for Exploratory Study Endpoints

The characterisation of early immune response events following immunization (days 0-3, 7-21 by gene expression and multiplex cytokine/cellular response analysis systems biology approach) have been used to successfully identify biomarkers of inflammation and immune response/efficacy. This has been done in a predictive and reproducible way by immunizing relatively small numbers of healthy subjects, for example with live viral Yellow Fever vaccine (Querec et al. 2009 Jan) or adjuvanted subunit malaria vaccines (Vahey et al., 2010). This opens up the novel possibility to conduct intensive and highly focused, but relatively small clinical trials early-on in the development of novel vaccine technologies to identify biomarkers that may be predictive of safety signals that may only become apparent in subsequent larger scale clinical testing or during post marketing surveillance.

At this stage it is not possible to define which genes will be analysed, and which metabolites, cytokine and acute phase proteins will be measured in serum as these analysis sets are continuously evolving as technology and knowledge advances.

Isolated PBMC will be available for genetic testing. Should specific biomarkers or clinical events be recorded, we may analyse the DNA for genetic markers to identify new variants associated with responses to vaccines, for validation in future clinical trials with larger numbers. All samples will be link-anonymised and no genetic data will be made available to subjects or their medical attendants. It is unlikely that even a sample size of 200 subjects will be able to identify rare variants, but as vaccine reactogenicity is not infrequent (up to 30% may experience some side effects) we may identify common traits. Also our biobank will provide a highly characterised set of samples to undertake future research should larger studies identify genetic traits associated with reactogenicity.

Blinding

This confirmatory study will be observer-blinded. For more details, see section 11.8.

STUDY OBJECTIVES AND ENDPOINTS

Exploratory Study Objectives

The purpose of this protocol is to generate a set of data that will be analysed by integrated systems biology approach, for validation in subsequent clinical trials or in animal models. The dataset will broadly characterise:

1. Physiological responses at various time points after immunisation by measuring:
    a. Local and systemic vaccine-related clinical events.
    b. Physiological assessments: heart rate, temperature, blood pressure.
    c. Haematology (blood counts and ESR), biochemistry parameters.
2. Metabolic, innate and adaptive immune responses including:
    a. Innate immune activation detected by global gene expression in whole blood
    b. Metabolic responses detected by metabolic gene expression and pathway activation in whole blood
    c. Adaptive immunity determined by:
        i. Humoral immune response via serum anti-influenza HAI titre
        ii. Cellular immune response via enumeration of HA-specific $CD4^+$ T cells expressing activation markers and/or cytokines following in vitro stimulation and analysis by flow cytometry.
    d. Innate and adaptive immune activation detected by gene pathway activation in whole blood
    e. Immune activation detected by concentration of selected inflammatory soluble mediators in serum including:
        i. chemokines and cytokines
        ii. acute phase proteins
3. Genetic testing of subjects when deemed necessary (genetic testing analysis may be SNIP analysis or full genome analysis).
4. Correlations in changes in innate and adaptive immune activation and metabolism with adverse events, haematology and biochemistry panels, genotype and physiological assessments

We will biobank all samples for the duration of the BIOVACSAFE programme so that we can selectively analyse different samples and different time points depending on the results generated, principally from the gene expression analysis of whole blood.

Study Endpoints (study variables measured)

The data set will include numerous study variables that will be analysed by integrated systems biology approach. Not all samples and not all time points will be initially analysed: an iterative approach is followed whereby putative biomarkers that are identified (principally in the gene expression data) will be further studied by measuring correlated variables such as immune responses, clinical events or serum / cellular responses. Samples will be biobanked during the project duration to allow further analyses that may be initiated by observations from the linked clinical studies.

Once the study objectives have been achieved samples will either be destroyed, entered into a registered biobank, or ethical approval sought for subsequent use.

The study variables will include:

1. Frequency of local and systemic vaccine-related clinical events at all time points from vaccination up to last study visit.
2. Change from pre-immunisation baseline values in pulse, temperature, blood-pressure at all time points from time of immunisation up to last study visit.
3. Change from pre-immunisation baseline values in haematology (blood counts and ESR), biochemistry parameters at selected time points from time of immunisation up to last study visit.
4. Change from pre-immunisation baseline values in global gene expression measured on whole blood samples at selected time points from time of immunisation up to last study visit
5. Change from pre-immunisation baseline values in metabolic gene expression and pathway activation measured on whole blood samples at selected time points from time of immunisation up to last study visit
6. Change from pre-immunisation baseline values in serum HAI titre in serum samples at selected time points from time of immunisation up to last study visit
7. Change from pre-immunisation values (Day 0) of adaptive cellular immune response will be evaluated at Day 7 in all subjects via enumeration of HA-specific $CD4^+$ T cells expressing activation markers and/or cytokines following in vitro stimulation and analysis by flow cytometry.
8. Change from pre-immunisation baseline values in concentration of selected cytokines and acute phase proteins in serum samples at selected time points from time of immunisation up to last study visit
9. Change from pre-immunisation baseline values in PBMC cytokine secretion, proliferation or surface markers in response to in vitro antigen stimulation at selected time points from time of immunisation up to last study visit
10. Genetic testing of subject

SUBJECT SELECTION AND WITHDRAWAL CRITERIA

Population base

Healthy male and female volunteers aged between 18 and 45 years.
There will be 240 subjects randomised to one of two groups:
A) Fluad Group, n = 228
B) Saline Placebo Group, n = 12

Inclusion criteria

- Healthy male or female subjects aged 18-45 years inclusive.
- Male: Female ratio - Screening will ensure that no more than 2/3 of the population should be of either male or female
- The subject is, in the opinion of the investigator:

1. Healthy based on medical history and clinical exam, with no active disease process that could interfere with the study endpoints.
2. Has a body Mass Index ≥18 and ≤30

3. Is able to read and understand the Informed Consent Form (ICF), and understand study procedures.
4. The subject has signed the ICF.
5. The subject is available for follow-up for the duration of the study.
6. The subject agrees to abstain from donating blood during their participation in the study, or longer if necessary.
7. If the subject is a heterosexually active female, she is willing to use an effective method of contraception with partner (oral contraceptive pill; intrauterine device; injectable or implanted contraceptive; condoms incorporating spermicide if using these; physiological or anatomical sterility) from 30 days prior to, and 3 months after, vaccination. Willing to undergo urine pregnancy tests prior to vaccination at screening.
8. The subject has venous access sufficient to allow blood sampling as per the protocol.

Exclusion criteria

1. Pregnant or lactating at any point during the study from screening to final follow up.
2. Hypersensitivity to the active components of FLUAD, any of the excipients, eggs, chicken proteins, kanamycin and neomycin sulphate, formaldehyde, and cetyltrimetholammonium bromide or those who have had a previous life-threatening reaction to previous influenza vaccinations.
3. Presence of primary or acquired immunodeficiency states with a total lymphocyte count less than 1,200 per mm^3 or presenting other evidence of lack of cellular immune competence e.g. leukaemias, lymphomas, blood dyscrasias, or patients receiving immunosuppressive therapy (including regular use of oral or parenteral corticosteroids).
4. Use of any immune suppressing or immunomodulating drugs within 6 months of Visit 1.
5. Regular use of non-steroidal anti-inflammatory drugs (oral or parenteral route) within 6 months of Visit 1 considered by the study physician as likely to interfere with immune responses.
6. Current intake of excessive amounts of alcohol and/or caffeine (as evaluated by the investigator) and not willing to adapt this use during the study period.
7. Currently performing extreme physical activities (as evaluated by the investigator) and not willing to adapt this use during the study period.
8. Receipt of a vaccine within 30 days of visit 1, or requirement to receive another vaccine within the study period.
9. Vaccination with the 2014/2015 seasonal influenza vaccine and/or any other seasonal influenza vaccine within the last 6 months before the first study visit.
10. Presence of an acute severe febrile illness at time of immunisation.
11. History of alcohol, narcotic, benzodiazepine, rilatine, or other substance abuse or dependence within the 12 months preceding Visit 1.

12. Currently participating in another clinical study with an investigational or non-investigational drug or device, or has participated in a clinical trial within the 3 months preceding Visit 1.
13. Any condition that, in the investigator's opinion, compromises the subject's ability to meet protocol requirements or to complete the study.
14. Receipt of blood products or immunoglobin, or blood donation, within 3 months of screening.
15. Unable to read and speak Dutch or English to a fluency level adequate for the full comprehension of procedures required in participation and consent.

No waivers from the Protocol will be allowed.

Discontinuation and Withdrawal of subjects from the study

Any subject may voluntarily discontinue participation in this study at any time.

The investigator may also, at his or her discretion, discontinue the subject from participating in this study at any time, and must do so if any of the following criteria are met:

- Positive pregnancy test at any visit.
- Concomitant use of any medication that may interfere with study outcome and study medication throughout the study.

The reason for termination will be recorded in the source notes and electronic case report form (eCRF). A subject may withdraw (or be withdrawn) from the study prematurely for the following reasons:

- Withdrawal of consent
- Adverse event (AE section must be completed)
- Protocol deviation
- Lost to follow-up
- Other (must be specified)

CLINICAL STUDY PROTOCOL

There will be two subgroups in this study: one half of the subjects (114 Fluad recipients and 6 placebo recipients) will undergo five outpatient visits (Days 0, 1, 3, 7 and 21) and will donate less blood for cell preparation at days 0 and 7 (90 ml instead of 120 ml) and the other half of the subjects (114 Fluad recipients and 6 placebo recipients) will undergo three outpatient visits (Days 0, 1, 7) and will donate more blood for cell preparation at days 0 and 7 (120 ml instead of 90 ml) Visit 1 will be screening and immunisation visit, visits 2 to 5 will be follow-up visits.

The study will start after a favourable opinion has been obtained from the Ethics Committee. Written informed consent will be obtained after a subject is informed of the nature, significance, implications and risks of the study and prior to the commencement of any study specific procedures.

Study Procedures

Visit 1: Screening and immunisation Visit (Day 0)

Subjects will come to CEVAC for VISIT 1 and the procedures listed in Table 2 will be performed.

After immunisation, the subject will be observed for 30 minutes. After this period, vital signs will be recorded and the subject will then be free to leave the center.

Visits 2, 3, 4 and 5: Follow Up Visits and phone contact (Days 1, 3, 7 and 21)

These follow-up visits will occur on specific days relative to immunisation.

Subject should attend visits 2 and 3 on the scheduled moment, i.e. day 1 and 3, respectively

A visit window of ±1 day is acceptable for visits 4 and 5 on days 7 and 21, respectively. However deviations from the proposed schedule should be avoided wherever possible and used only in a situation where a sample would otherwise be lost. Outside of this the visit procedures should still be undertaken and all samples collected, but will be recorded as a protocol deviation. The procedures listed in Table 2 will be performed.

Duration of Study Participation

Each subject will be participating in the study for 21 days. For subjects of the 5-visit subgroup Day 21 is evidently the last visit. Subjects of the 3-visit subgroup will be contacted by telephone call on Day 21 for a final check of their health status.

Study Restrictions: prohibited vaccination and medication

Subjects will be required to comply with the following for the duration of the study:

- Subjects must not receive another vaccine within 30 days of visit 1, or at any time during the study period. If a subject receives a non-study vaccine before the end of the study, the subject can continue the study for safety follow-up, but no more blood samples will be collected.
- If a subject uses oral, inhaled, topical or injectable non-steroidal anti-inflammatory drugs or any immunosuppressing or immunomodulating drugs during the course of the study, the subject can continue the study for safety follow-up, but no more blood samples will be collected

STUDY MEDICATION

11.1 Treatment Plan

GROUP A: The study medication will be FLUAD – Adjuvanted subunit vaccine. The study medication will be supplied as a 0.5 ml pre-filled syringe containing 15µg of influenza virus haemagglutinin surface antigens from each of the three virus strains (A/H1N1, A/H3N2 and B).

GROUP B: The placebo control will be 0.5 mL physiological saline administered in a 1 mL syringe with a needle gauge for administration equivalent to the vaccines.

For both groups, the vaccination will be administered intramuscularly into the deltoid muscle, according to the SmPC.

11.2 Preparation, Administration and Dosage of Study Medication

11.2.1 Preparation Instructions

The FLUAD vaccine is supplied as pre-filled syringe ready for use.

PLACEBO control: 0.5 mL physiological saline must be drawn into a 1 mL syringe with a needle gauge for administration equivalent to the vaccine.

11.2.2 Route of administration

The vaccine will be administered intra-muscularly into the deltoid muscle of the non-dominant arm, according to the SmPC. Alcohol and other disinfecting agents must be allowed to evaporate from the skin before injection of the vaccine since they may inactivate the virus.

11.2.3 Dose and Dosing Schedule

A single 0.5 mL vaccine dose will be administered intramuscularly.

The dose and route selected is that described in the SmPC.

11.3 Packaging and Labelling

The following particulars will be added to the original container, but will not obscure the original labelling:
i) CEVAC
ii) Study reference code, investigator and study participant.
iii) Vaccine number (Fluad vaccines will be numbered starting from 500, saline will be numbered starting from 800)

11.4 Storage and Accountability of Drug Supplies

The vaccines should be stored at +2°C to +8°C (in a refrigerator).

Simplified accountability records will be kept, to capture the batch number of the product dispensed on a study-specific dispensing form, filed in a study folder to permit retrospective verification if this was necessary.

11.5 Destruction of Clinical Supplies

Any unused vaccines will remain in their original packaging. After study medication accountability has been completed they will be disposed of by discard into the standard clinical waste system.

11.6 Responsibilities

- Named trained study nurses will be responsible for ensuring that the vaccine is securely maintained.
- Named trained study nurses will be responsible for ensuring that an accurate record of vaccine issued and returned is maintained.
- Vaccine quality issues will be reported to the manufacturer by the PI.

11.7 Compliance

Vaccines will be administered at the clinical site by trained study nurses.

11.8 Treatment Assignment Procedures

Once the informed consent form has been signed and the subject has been found eligible for participation, he/she will be allocated the lowest available subject number. The subject numbers available will be 001 to 240. Each number will be linked to one of both treatment groups, Fluad or placebo. The corresponding treatment group will be read from a randomisation list, which will only be accessible to the unblinded team.

If a replacement subject is needed, the unblinded vaccine administrator, can refer to the parallel replacement randomisation list (subject numbers from 1001 to 1240). The replacement subject will be allocated the corresponding number on the replacement list (for example, 081 would be replaced by 1081) and both would be in the same treatment group.

All vaccines will be numbered. The vaccine administrator will take the lowest available vaccine number (from the assigned treatment group) out of the fridge.

11.8.1 Randomisation

A randomisation list will be provided in a sealed envelope by Surrey CRC, which can only be opened by an unblinded team member. A program to generate a randomisation list linking subject numbers to treatment group will be produced by an independent statistician using a computer-generated algorithm. An independent member of staff will then use this program to produce the randomisation list. All eligible subjects that pass screening will be assigned a subject number, which corresponds to a certain treatment group. Sealed emergency code break envelopes will be available.

11.8.2 Blinding/Unblinding

As this is an observer blind study, the treatment allocation, vaccine preparation and vaccine administration will be handled by (an) unblinded team member(s). The randomisation list will be kept in a sealed envelope. At the end of each day, after vaccination, the list will be resealed, until needed.

The unblinded team member(s) will not be involved in any other trial activities. All other staff involved in the trial will be blinded (blinded team at the investigational site, laboratories). Also subjects will be blinded.

The treatment allocation will be unblinded after the final visit on day 21 of the last subject enrolled and after all clinical events have been assessed, in order for the placebo recipients to receive a seasonal influenza vaccination in time.

<u>STUDY DATA PARAMETERS</u>

12.1 Laboratory Assessments

Blood samples will be collected at the time points specified in Table 1. Standard laboratory tests, including chemistry, haematology, and urinalysis panels, will be performed. Pregnancy tests will be performed for female subjects at screening, prior to vaccine/placebo administration. Blood samples for transcriptomics (Paxgene),

proteomics (serum), humoral (serum) and cellular immune responses (heparinized blood) will be collected at time points specified in the study flow charts.

‘Serum Save’
Several of the parameters being investigated will be analysed in serum. A ‘serum save’ strategy will be used to ensure that enough serum is collected at each time point for each of the required parameters to be measured. The ‘serum save’ strategy applies to the following sample types: acute phase proteins, chemokines and cytokines, serum immunology samples, and ‘biobank’ samples. In practice, when a blood sample is collected the extracted serum will be separated into serum aliquots. These will be stored and made available for analysis within the 5-year Biovacsafe project time period.

‘Cell Save’
Several of the parameters being investigated will be analysed using PBMC. A ‘cell save’ strategy will be used to ensure that enough cells are collected at selected time points for each of the required parameters to be measured. The ‘cell save’ strategy applies to the following applications: mRNA expression in selected leukocyte populations, antigen-specific cell-mediated immune responses, regulatory T cells, and ‘biobank’ samples. In practice, when a blood sample is collected the extracted PBMC will be separated and divided into several aliquots. These will be stored and made available for analysis within the 5-year Biovacsafe project time period.

Biobank
At every time point a serum sample is required. Enough blood will be collected to extract the required volume of serum for the parameter(s) being measured plus 1ml of additional serum, which will be entered into a biobank. PBMC will be harvested from heparinized blood to examine different aspects of cell-mediated immunity and remaining cells will be entered in the biobank. The biobank will be used to analyse other parameters which become of interest during the study and which would contribute to the aims of the Biovacsafe project. Every effort will be made to collect only the minimum volume of blood from participants. However, on occasion it is possible that slightly more serum and cells will be extracted from the blood samples than is required. Should this occur, the remaining serum and cells will also be added to the biobank.

Genetic testing
One blood sample for DNA genotyping will be collected for each subject prior to vaccine/placebo administration, for the purposes described in section 7.5. The blood sample will be anonymised so that it will not be possible to link the results of the genotyping back to the subject that provided the sample, other than through the investigator site. Genetic analysis (SNP, sequence analysis, ..) will only be executed when deemed necessary by the consortium

12.1.1 Study Blood Volume

The total volume taken during the study is detailed in Table 3 and Table 4, for the 5-visit and the 3-visit study groups, respectively. The total volume taken will be approximately 327,5 ml (5-visit) and 328,5 ml (3-visit).

The study blood volumes already include the blood that will be drawn to evaluate the antigen-specific cellular immune responses (CMI) as well as the mRNA expression profiles in defined white blood cell subsets (granulocytes, lymphocytes and monocytes) that will be isolated from the whole blood in the 3-visit subset of the study.

12.1.2 Urinalysis

Urinalysis will be performed by dipstick to evaluate protein, glucose, pH, white and red blood cells. Microscopy may be performed if the dipstick is clinically significant.

12.1.3 Urine pregnancy test

A urine pregnancy test will be done at visit 1 for all female subjects of childbearing potential.

12.2 Vital signs

Vital signs include: diastolic and systolic blood pressure measurement heart rate and oral temperature. These will be obtained at all study visits.

12.3 Measurement of temperature and injection site reactions

Subjects will be provided with a thermometer to measure the oral temperature on day 0 to day 7 at approximately 08:00 am, approximately noon; approximately 04:00 pm and at 10:00 pm (or bed time). A ruler will be given to measure the largest diameter of local adverse events, namely redness, swelling and induration at injection site. Subjects will also need to register if they experience any local pain. These symptoms will be actively registered from Day 0 to Day 7, in the subject diary cards.

Next to the local symptoms, subjects will need to register the intensity of a number of general symptoms in the period Day 0 to Day 7, namely headache, fatigue, gastro-intestinal symptoms (nausea, vomiting, diarrhea and/or abdominal pain) and generalised myalgia. This will be recorded in the subject diary cards.

If the subjects experience any other symptoms, they will be instructed to write this down in their diary cards.

The intensity of the symptoms will be graded as following:
0: None
1: Mild: Does not interfere with or prevent normal every day activities.
2: Moderate: Interferes with every day activities, but doesn't prevent the subject from doing them.
3: Severe: Prevents normal every day activities

12.4 Methods and Timing for Assessing and Recording Study Data Parameters

Laboratory safety tests (biochemistry, haematology, urinalysis) will be performed at all study visits (3-visit or 5-visit subgroups). The detailed outline of the study procedures can be found in Table 2.

Table 3: Volumes of blood collected in the 5-visit group

Parameter	**Day 0** Volume ml	**Day 1** Volume ml	**Day 3** Volume ml	**Day 7** Volume ml	**Day 21** Volume ml	**Total Volume ml**
Laboratory safety	8*	8	8	8	8	40
Paxgene (RNA)	7,5	7,5	7,5	7,5	7,5	37.5
Whole blood for PBMC isolation	90			90		180
Serum (immunology)	14	14	14	14	14	70
**TOTAL (ml)**	**119,5**	**29,5**	**29,5**	**119,5**	**29,5**	**327,5**

*** Screening serology (hepatitis B and C, HIV) inclusive**

Table 4: Volumes of blood collected in the 3-visit group

Parameter	**Day 0** Volume ml	**Day 1** Volume ml	**Day 7** Volume ml	**Total Volume ml**
Laboratory safety	8*	8	8	24
Paxgene (RNA)	7,5	7,5	7,5	22,5
Whole blood for PBMC isolation	120		120	240
Serum (immunology)	14	14	14	42
**TOTAL (ml)**	**149,5**	**29,5**	**149,5**	**328,5**

*** Screening serology (hepatitis B and C, HIV) inclusive**

SAFETY MONITORING

All subjects will receive a study information card that indicates their participation in the study and contact information for the study site.

13.1 Specification of Safety Parameters

13.1.1 Laboratory and Other Safety Assessment Abnormalities Reported as AEs and SAEs

Any abnormal laboratory test results (haematology, biochemistry or urinalysis) or other safety assessments (e.g. vital signs measurements), including those that worsen from baseline, and are felt to be clinically significant in the medical and scientific judgement of the investigator, are to be recorded as AEs or SAEs.

However, any clinically significant safety assessments that are associated with the underlying disease, unless judged by the investigator to be more severe than expected for the subject's condition, are **not** to be reported as AEs or SAEs.

13.1.2 Definition of an AE

- Any untoward medical occurrence in a patient or clinical study subject, to whom a medicinal product has been administered and which does not necessarily have a causal relationship with this treatment.

Note: An AE can therefore be any unfavourable and unintended sign (including an abnormal laboratory finding), symptom, or disease (new or exacerbated) temporally associated with the use of a study medication whether or not related to the study medication. For marketed medicinal products, this also includes failure to produce expected benefits (i.e. lack of efficacy), abuse or misuse.

Events meeting the definition of an AE include:

• Exacerbation of a chronic or intermittent pre-existing condition including either an increase in frequency and/or intensity of the condition

• New conditions detected or diagnosed after study medication administration even though it may have been present prior to the start of the study

• Signs, symptoms, or the clinical sequelae of a suspected interaction

• Signs, symptoms, or the clinical sequelae of a suspected overdose of either study medication or a concomitant medication (overdose *per se* will not be reported as an AE/SAE).

Events that **do not** meet the definition of an AE include:

• Medical or surgical procedure (e.g. endoscopy, appendectomy); the condition that leads to the procedure is an AE.

• Situations where an untoward medical occurrence did not occur (social and/or convenience admission to a hospital).

• Anticipated day-to-day fluctuations of pre-existing disease(s) or condition(s) present or detected at the start of the study that do not worsen.

• The disease/disorder being studied or expected progression, signs, or symptoms of the disease/disorder being studied, unless more severe than expected for the subject's condition.

13.1.3 Definition of a SAE

An SAE based on ICH and EU Guidelines on Pharmacovigilance for Medicinal Products for Human Use is any untoward medical occurrence that at any dose:

(a) results in death;
(b) is life-threatening;
(c) requires hospitalisation or prolongation of existing hospitalisation;
(d) results in persistent or significant disability or incapacity;
(e) consists of a congenital anomaly or birth defect; or
(f) is otherwise considered medically significant by the investigator.

13.2 Evaluating and Recording (Serious) Adverse Events

The investigator or designee is responsible for detecting, documenting and reporting events that meet the definition of an AE or SAE. These must be recorded in the source adverse event form and CRF.

For all adverse events, the following must be assessed and recorded on the adverse events page of the CRF:

a) Description of AE
b) Start date and time
c) End date and time

d) Severity i.e. mild, moderate, severe (see below)
e) Relationship to study medication(s) – completed by clinical research physician
f) Outcome
g) Seriousness (see section on Serious Adverse Events)

Subjects will be kept under observation for 30 minutes after vaccination to ensure their safety.
All AEs starting within 21 days following vaccination must be recorded into the appropriate section of the CRF, irrespective of intensity or whether or not they are considered vaccination-related.
The time period for collecting and recording SAEs will begin at the receipt of study vaccine and will end at Day 21 (study end).
An overview of the protocol-required reporting periods for AEs, SAEs, and pregnancies is given in Table 5.

SAEs that are spontaneously reported by a subject to the investigator after study completion and considered by the investigator to be caused by the study medication with a reasonable possibility should be handled in the same manner as for SAEs reported during the study.

In the event of unexplained clinically abnormal laboratory test values, the tests should be repeated immediately and followed up until the results have returned to within the range of normal and/or an adequate explanation of the abnormality is given.

Table 5: Reporting periods for adverse events, serious adverse events and pregnancies

**Study activity**	**Vaccination (Visit 1)**	**(Visit 2)**	**(Visit 3)**	**(Visit 4)**	**Study conclusion (Visit 5/Phone contact)**
**Timing of reporting**	**Day 0**	**Day 1**	**Day 3**	**Day 7**	**Day 21**
**Reporting of solicited AEs**					
**Reporting of Unsolicited AEs**					
**All SAEs**					
**Reporting of pregnancies**					

13.3 Intensity of Event

The intensity of an adverse event is defined as follows:

Mild Transient symptoms, requiring no treatment, no interference with subject's daily activities, easily tolerated.

Moderate Marked symptoms, moderate interference with the subject's daily activities, usually ameliorated by simple therapeutic measures.

Severe Considerable interference with the subject's daily activities, requires intensive therapeutic intervention, incapacitating.

The term **severe** is a measure of **intensity**: thus a severe AE is not necessarily **serious.** For example, nausea of several hours duration may be rated as severe, but may not be clinically serious.

13.4 Relationship to study product

The relationship of each adverse event to the study medication must be recorded by a medically qualified member of staff as one of the following scale:

Definitely Not related The AE is judged to be clearly and incontrovertibly due only to extraneous causes (for example, disease, environment) definitely not associated with the test drug being given and does not meet the criteria for any other drug relationship listed.

Probably Not related In general, this category is applicable to an AE which meets the following criteria (it certainly must meet the first two criteria):

1. It does not follow a reasonable temporal sequence from the drug administration.
2. It may readily have been produced by the subject's clinical state, environmental or toxic factors, or other modes of therapy administered to the subject.
3. It does not follow a known pattern of response to the suspected drug.
4. It does not reappear or worsen when the drug is re-administered.

Possibly Related This category applies to those AEs in which the connection with the test drug administration appears unlikely but cannot be ruled out with certainty. An AE may be considered as possibly drug related if, or when:

1. It follows a reasonable temporal sequence from administration of the drug.
2. It may have been produced by the subject's clinical state, environmental or toxic factors or other modes of therapy administered to the subject.
3. It follows a known pattern of response to the suspected drug.

Probably Related This category applies to those AEs which are considered, with a high degree of certainty, to be related to the test drug. An AE may be considered as probably drug related if:

1. It follows a reasonable temporal sequence from administration of the drug.
2. It cannot be reasonably explained by the known characteristics of the subject's clinical state, environmental or toxic factors, or other modes.
3. It disappears or decreases on cessation or reduction in dose (there are important exceptions when an AE does not disappear upon discontinuation of the drug, yet drug-relatedness clearly exists).
4. It follows a known pattern of response to the suspected drug.
5. It reappears upon re-challenge.

Definitely Related This category applies to those AEs which are considered to be definitely related to the test drug. An AE may be considered as Definitely related if:

1. There is evidence of exposure to the test drug.
2. It follows a reasonable temporal sequence from administration of the drug.
3. It cannot be reasonably explained by the known characteristics of the subject's clinical state, environmental or toxic factors, or other modes.
4. The AE is more likely explained by the test drug than by any other cause.
5. De-challenge is positive.
6. Re-challenge (if feasible) is positive.
7. The AE shows a pattern consistent with previous knowledge of the test drug or test drug class.

13.5 Expectedness of SAEs

For this study, an adverse reaction is 'unexpected' if its nature and severity are not consistent with the information about the study medication in question, set out in the SmPC. A medically qualified member of staff must assign expectedness.

13.6 Reporting of serious adverse events, pregnancies, and other events

All SAEs and pregnancies occurring during clinical trials must be reported by the Investigator within 2 working days after becoming aware of the SAE to:

- The local Ethic Committee (EC)
- Bimetra Clinics of the University Hospital Ghent

This reporting is done by using the appropriate SAE form. For the contact details, see below.
In case the investigator decides the SAE is a Suspected Unexpected Serious Adverse Reaction (SUSAR), Bimetra Clinics will report the SUSAR to the Central EC and the competent authorities (CA) within the timelines as defined in national legislation.
In case of a life-threatening SUSAR the entire reporting process must be completed within 7 calendar days. In case of a non life-threatening SUSAR the reporting process must completed within 15 calendar days.
The first report of a SAE may be made by telephone, e-mail or facsimile (FAX).
Contact details of Bimetra Clinics:

e-mail: bimetra.clinics@uzgent.be
tel.: 09/332 05 00
fax: 09/332 05 20

The investigator must provide the minimal information: i.e. trial number, subject's initials and date of birth, medication code number, period of intake, nature of the AE and investigator's attribution.
This report of a SAE by telephone must always be confirmed by a written, more detailed report. For this purpose the appropriate SAE form will be used. Pregnancies occurring during clinical trials are considered immediately reportable events. They must be reported as soon as possible using the same SAE form. The outcome of the pregnancy must also be reported.

STATISTICAL CONSIDERATIONS AND ANALYTICAL PLAN

14.1 Responsibility for Analysis

Data management for this study will be carried out by Surrey CRC. The analysis of the data obtained from this study will be the responsibility of the BioVacSafe consortium. Additional integrated systems biology analysis may be undertaken by specialist laboratories and collaborators.

14.2 Justification of Sample Size

This study will utilize an integrated systems biology approach to assess acute and longitudinal responses of all measured variables to immunization to identify exploratory putative biomarkers. These may then be further characterised and confirmed in subsequent trials and animal models. As such, a variety of –omic technologies will be used to identify novel biomarkers in an iterative fashion where the biobank of samples is repeatedly accessed; as the identity of these markers and the magnitude of response are unclear, it is not possible to use existing data to perform power calculations and determine an appropriate sample size. In addition the samples and data from all the clinical training trials (involving 5 different vaccines) will be combined into the integrated analysis, altogether with the data from the two larger trials that will follow (around 600 subjects) and the various animal models.

A previous study utilized a systems biology approach to detect early gene 'signatures' to predict immune responses in individuals immunized with the yellow fever vaccine YF-17D (Querec et al., 2009). With a sample size of 15 it was possible to detect distinct signatures that predicted the neutralizing antibody response with up to 100% accuracy.

For this first confirmatory study a sample size of n = 240 (228 active compound and 12 placebo controls) has been selected as this should allow for significant changes in –omic profiles to be detected. The placebo groups from the two confirmatory studies will be pooled to produce a comparable group of n = 24.

14.3 Definition of Study Completion

Study completion is defined as the date the last subject completes the final visit in the study.

14.4 Definition of Criteria for Termination of the Study

Study termination is defined as a permanent discontinuation of the study due to unanticipated concerns of safety to the study subjects arising from AEs recorded during the study that are definitely related to the protocol, and are not present in the SmPC; or availability of other new data (pharmacokinetic, pharmacodynamic, efficacy, biologic etc.) arising from clinical or preclinical studies with this study drug. A study may be paused during review of newly available preclinical/clinical safety, pharmacokinetic, pharmacodynamic, efficacy, or biologic data, or other issues of interest or potential concern prior to a final decision for continuation or termination of the study.

14.5 Analysis of Clinical Events and Exploratory Endpoints after immunisation

All subjects who receive a dose of study medication will be included.

All AEs will be coded using MedDRA prior to database lock. Adverse events will be analysed, irrespective of their causal relationship, by treatment group. The terms mentioned in the tabulations will be the MedDRA organ system and preferred terms.

For Serious Adverse Events meeting the definition of section 13.1, tabulations will provide by treatment group, the number of subjects exposed, the number of subjects with at least one Serious Adverse Event, the number of subjects with at least one Serious Adverse Event by organ system and preferred term. Separate tables will be provided, if relevant, for SAEs leading to withdrawal from study.

STUDY DOCUMENTATION ADMINISTRATION

15.1 Source documents and eCRF

The eCRF/database will be provided/maintained by Surrey CRC.

All evaluations that are reported in the eCRF must be supported by appropriately signed identified source documentation.

Paper workbooks are provided for each subject, and all data related to the study will be recorded in these workbooks. These source documents are to be completed at the time of the subject's visit so that they always reflect the latest observations on the subjects.

The investigator must verify that all data entries in the workbooks are accurate and correct by signing the relevant pages. If certain information is not available, not applicable, not done or unknown, the clinical staff or investigator will enter the relevant abbreviation, i.e. NA to confirm that the data field has not been overlooked.

This also applies to subjects who fail to complete the study. If a subject withdraws from the study, the reason must be noted on the workbook. If a subject is withdrawn from the study because of a treatment-limiting adverse event, thorough efforts should be made clearly to document the outcome.

All forms should be typed or filled out using a black ball-point pen, and must be legible. All entries, corrections and alterations are to be made by the responsible investigator or her/his designee. With the exception of obvious mistakes, the corrections need to be commented. Corrections should be made in such a way that the original entry is not obscured. The corrected data should be entered, dated, and initialled by the investigator or his designee.

The information recorded in the paper workbooks will be transcribed into the eCRF following the subjects visit and within 3 working days.

15.2 Monitoring

Monitoring will be performed by Bimetra Clinics in accordance with applicable regulations and standards of GCP.
When reviewing data collection procedures, discussion will include identification, agreement and documentation of data items for which the workbook will serve as the source document.
The monitor will review the workbooks/eCRFs, evaluate them for accuracy (including source data verification) and completeness, and return all forms with missing information and/or errors to the clinical staff or investigator for correction.
The monitor will supervise the study to ensure that the:

- Data are authentic, accurate, and complete
- Safety and rights of subjects are being protected
- Study is conducted in accordance with the currently approved protocol and any other study agreements, GCP and all applicable regulatory requirements

15.3 Access to Source Data Documents

The investigator will permit trial–related monitoring, audits, IRB/IEC review, and regulatory inspections by providing direct access to source data/documents.

15.4 Data Handling and Record Retention

The investigator must maintain adequate records to enable the conduct of the study to be fully documented. The investigator should arrange for retention of the essential documents in the investigator's Trial Master File for at least twenty years after the final study report has been signed.

15.5 Subject Confidentiality and Data Protection

The investigator must ensure that subject's anonymity will be maintained on eCRFs or other documents and blood/urine samples (lab reports, etc). Subjects should **not** be identified by their names, but by a subject number. The investigator should keep a

separate enrolment log showing subject numbers, names and date of birth. Documents such as subjects' separate written consent forms should be maintained by the investigator in strict confidence.

QUALITY CONTROL AND QUALITY ASSURANCE

To ensure compliance with GCP and all applicable regulatory requirements, the sponsor may conduct a quality assurance audit of the site records, and the regulatory agencies may conduct a regulatory inspection at any time during or after completion of the study. In the event of an audit or inspection, the investigator (and institution) must agree to grant the auditor(s) and inspector(s) direct access to all relevant documents and to allocate their time and the time of their staff to discuss any findings/relevant issues.

A study monitor from Bimetra Clinics is responsible for visiting the institution at regular intervals throughout the study to verify adherence to the protocol; completeness, accuracy, and consistency of the data; and adherence to ICH GCP and local regulations on the conduct of clinical research. The monitor is responsible for inspecting the workbooks/eCRFs and ensuring completeness of the study essential documents. The monitor should have access to subject medical records and other study-related records needed to verify the entries on the workbooks/eCRFs. The monitor will communicate deviations from the protocol, SOPs, GCP and applicable regulations to the investigator and will ensure that appropriate action designed to prevent recurrence of the detected deviations is taken and documented. The investigator agrees to cooperate with the monitor to ensure that any problems detected in the course of these monitoring visits are addressed and documented.

CLINICAL STUDY PROTOCOL DEVIATIONS AND AMENDMENTS

Any 'substantial' protocol amendment(s) (meaning that it could have a significant impact on the safety or physical or mental integrity of the subjects, the scientific value of the study, the conduct or management of the study, the quality or the safety of any study vaccines used in the study) must be submitted to the Independent Ethics Committee (IEC) and, if applicable, the competent authority prior to its implementation.

Amendments to exploratory endpoints, objectives, parameters and variables will not be considered substantial.

For non-substantial changes that do not affect safety or study validity e.g. an administrative change, the competent authority is not required to be notified. Non-substantial amendments will be reported to the competent authority at the time of a subsequent substantial amendment. Non-substantial amendments will be reported to the IEC.

In the case of changes consisting of urgent safety measures to protect the study subjects, the sponsor should inform the IEC as soon as possible after these measures have been implemented.

CONDITIONS FOR TERMINATING THE STUDY

Study completion is defined as the date the last subject completes the final visit in the study. Study termination is defined in section 10.2.

If, in the opinion of the investigator, the clinical observations or pharmacokinetic profiles in the study suggest that it may be unwise to continue, the investigator may terminate part of, or the entire study, after consultation with the sponsor, or the sponsor may terminate part of, or the entire study, for safety or administrative reasons. A written statement fully documenting the reasons for such termination will be provided to the IEC.

ETHICAL AND REGULATORY REQUIREMENTS

The trial will be conducted in compliance with the protocol, principles of GCP, Data Protection Act and other regulatory requirements, as appropriate, and will abide by the principles of the 2013 revision of the Declaration of Helsinki.

19.1 Informed Consent

It is the responsibility of the investigator to obtain written informed consent from each subject participating in this study, after adequate explanation of the aims, methods, anticipated benefits, and potential hazards of the study. This includes obtaining the appropriate signatures and dates on the informed consent document prior to the performance of any study specific procedures. Subjects will be given written information outlining the study details given approval by the IEC. Any changes to the approved version of the information sheet/consent form must be approved by the IEC prior to its implementation, unless it is for urgent safety measures. A copy of the signed consent form will need to be given to the subject.

The investigator, or person under his responsibility, must also explain that the subject is completely free to refuse to enter the study or to withdraw from it at any time. The CRF for this study contains a section for documenting informed consent, and the investigator, or person under his responsibility, must complete it appropriately.

19.2 Independent Ethics Committee (IEC)

This protocol and any accompanying material provided to the subjects (such as the information sheet and consent form) will be submitted by the investigator, or person under his responsibility, to the appropriate IEC. Approval from the committee must be obtained in writing before starting the study and the approval letter must reference which documents were reviewed and approved.

Any required changes will be forwarded to the IEC for their approval. Written approval of the revised documents should also be obtained from the IEC. Depending upon the exact changes, written approval of the revised documents may not be required prior to the commencement of the screening process.

The IEC must provide a copy of their membership list, and a list of names of those members reviewing the study. All documents related to IEC correspondence will be kept at CEVAC.

19.3 Final Reports

The sponsor will notify the IEC within 90 days of the end of the study. If the study is terminated prematurely, this reporting timeframe will be reduced to 15 days from the termination of the study.

The study report will be provided to the IEC within one year of completion of the study.

19.4 Competent Authority

Before initiating this clinical trial, the sponsor, with help from Bimetra, will submit any required applications to the appropriate authority for review and acceptance. Bimetra will forward documents related to CA correspondence, so (at least) a copy can be kept at CEVAC.

FINANCE AND INSURANCE

The sponsor, UZ Gent: CEVAC, maintains an insurance to cover its liability or the liability of any person involved in the conduct of the study, in accordance with the requirements of the Belgian law dated 07 May 2004.
All staff working for CEVAC is insured by the University Hospital Insurance policy (Insurance company: KBC and AMLIN).

PUBLICATIONS

Before recruitment the trial will be registered with clinicaltrials.gov (http://clinicaltrials.gov/) website.

All publications and communications arising from this trial will comply with the Project and Grant Agreements for the Innovative Medicines Initiative project BIOVACSAFE proposal number 115308.

REFERENCES

Pulendran, B, Li, S and Nakaya,, HI (2010). Systems vaccinology. *Immunity*, 33, 516 – 29.

Querec, TD, Akondy, RS, Lee, EK, Cao, W, Nakaya, HI, Teuwen, D, Pirani, A, Gernert, K, Deng, J, Marzolf, B, Kennedy, K, Wu, H, Bennouna, S, Oluoch, H, Miller, J, Vencio, RZ, Mulligan, M, Aderem A, Ahmed, R, Pulendran, B (2009). Systems biology approach predicts immunogenicity of the yellow fever vaccine in humans. *Nat Immunol*, 10, 116 – 25.

Vahey, MT, Wang, Z, Kester, KE, Cummings, J, Heppner, DG Jr, Nau, ME, Ofori-Anyinam, O, Cohen, J, Coche, T, Ballou, WR, Ockenhouse, CF (2010). Expression of genes associated with immunoproteasome processing of major histocompatibility complex peptides is indicative of protection with adjuvanted RTS, S malaria vaccine. *J Infect Dis*, 15, 580 – 9.

23 Laboratory Safety Analysis.

BLOOD	ESR<br>White blood cell count<br>Haemoglobin<br>Platelets<br>Red blood cell<br>Haematocrit<br>MCV<br>MCH<br>MCHC<br>RDW<br>neutrophils<br>lymphocytes<br>monocytes<br>eosinophils<br>basophils<br>creatinine<br>eGFR<br>albumin<br>CRP (standard method – lower sensitivity)<br>high sensitivity CRP<br>ALT/AST<br>GGT<br>total protein
URINE	Urine dipstick analysis:<br>glucose<br>protein<br>WBC<br>bilirubin<br>bacteria<br>RBC<br>pH
